# Supplementary material for: EphA2 and phosphoantigen-mediated selective killing of medulloblastoma by γδT cells preserves neuronal and stem cell integrity
Source: Oncoimmunology. 2025 Apr 7;14(1):2485535. doi: 10.1080/2162402X.2025.2485535 (PMC11980450; doi:10.1080/2162402X.2025.2485535)
Supplement: Boutin et al_Table_S4.pdf [file KONI_A_2485535_SM7966.pdf]

Table S4

Boutin et al.

Ephrin-A2 and Phosphoantigen-Mediated Selective Killing of Medulloblastoma by γδT Cells Preserves Neuronal and Stem Cell Integrity

**CDR3 analysis**										
**Barcode**	**cell_type**	**V_chain1**	**V_chain2**	**J_chain1**	**J_chain2**	**C_chain1**	**C_chain2**	**CDR3_chain1**	**CDR3_chain2**	**Note**
1066_GTGAGGACACAAGGTG	abT	TRBV7-9	*	TRBJ2-7	*	*	*	CASSSKAGGFYEQYF	*	
1195_AGAAATGGTGGTGATG	abT	TRBV6-5	*	TRBJ2-3	*	*	*	CASQRAGAADTQYF	*	
1224_CGAAGTTGTAGCACAG	abT	TRBV7-6	*	TRBJ2-2	*	*	*	CASGYASGELFF	*	
1224_GGGATCCCACAGAGCA	abT	TRBV7-2	*	TRBJ2-1	*	TRBC2	*	CASAIEAGAPNEQFF	*	
1238_AATGGCTGTCACTCTC	abT	TRBV23-1	*	TRBJ1-5	*	TRBC1	*	CASSPHLQGPYQPQHF	*	
1238_ACCAACAGTATTTCTC	abT	TRBV6-7	*	TRBJ2-7	*	*	*	CNIVGTVSYEQYF	*	
1238_AGGAATAGTCACTTCC	abT	TRBV23-1	*	TRBJ1-5	*	*	*	CASSPHLQGPYQPQHF	*	
1238_GATCACATCCACCTCA	abT	TRBV20-1	*	TRBJ2-7	*	*	*	CSAISPVSVGEQYF	*	
1238_TCCATGCGTAGCTGTT	abT	TRBV2	*	TRBJ2-5	*	TRBC2	*	CASSYGQGAYETQYF	*	
1416_CAACCAAAGGACATCG	abT	TRBV20-1	*	TRBJ2-3	*	TRBC2	*	CSARDPGGLAYTDTQYF	*	
925_CTTGATTGTTCTCCAC	abT	TRBV6-2	*	TRBJ2-5	*	*	*	CASSLAGGSWTQYF	*	
925_GCCGTGACAAGTGGTG	abT	TRBV20-1	*	TRBJ1-1	*	*	*	CSAWDSTEAFF	*	
925_TATTTCGTCGTGTCAA	abT	TRBV20-1	*	TRBJ2-7	*	*	*	CSARVTAGGSSYEQYF	*	
943_AGTAACCCACCGCTGA	abT	TRBV15	*	TRBJ2-7	*	*	*	CATSRDGTGDSYEQYF	*	
943_CCTCCAAGTGACTATC	abT	TRBV7-9	*	TRBJ1-2	*	*	*	CASSRLLTHYGYTF	*	
898_CATCCGTTCTCATTTG	abT	*	TRAV10	*	TRAJ7	*	TRAC	*	CVTVGPGNNRLAF	
996_TACAACGAGCGCTTCG	abT	*	TRAV12-1	*	TRAJ11	*	*	*	CVVNGGSGYSTLTF	
1238_GGTAATCCATAATGCC	abT	*	TRAV12-1	*	TRAJ37	*	*	*	CVVGSSNTGKLIF	
925_GCCATGGCACAGTGAG	abT	*	TRAV12-1	*	TRAJ39	*	*	*	CVVNINNAGNMLTF	
1224_TTTCAGTAGGGACCAT	abT	TRBV28	TRAV12-1	TRBJ2-3	TRAJ9	*	TRAC	CASSFPDTQYF	CVVPSDTGGFKTIF	
1238_GAGACTTCAACACAGG	abT	*	TRAV12-2	*	*	*	TRAC	*	CAVNQAGTALIF	
1238_GTAGAGGAGTGAGCCA	abT	*	TRAV12-2	*	*	*	*	*	CAVNQAGTALIF	
831_TACAACGTCAGCTCTC	abT	*	TRAV12-2	*	TRAJ24	*	TRAC	*	CAVNSRTDSWGKLQF	
1238_GCAGCCATCAGACATC	abT	*	TRAV12-2	*	TRAJ3	*	TRAC	*	CAVIPRGYSSASKIIF	
934_GATGGAGGTAACGGTG	abT	*	TRAV12-3	*	TRAJ17	*	TRAC	*	CAMLKAAGNKLTF	
898_TACGCTCCAATAGGGC	abT	*	TRAV12-3	*	TRAJ8	*	*	*	CAMSRTGFQKLVF	
1130_AACCCAATCACTTTGT	abT	*	TRAV13-1	*	TRAJ13	*	*	*	CAATLSGGYQKVTF	
925_GCCCGAAGTTGTCCCT	abT	*	TRAV13-1	*	TRAJ31	*	TRAC	*	CAASNGNNARLMF	
1238_GTGAGCCTCGGTAACT	abT	*	TRAV13-1	*	TRAJ48	*	*	*	CAALYEKLTF	
1224_GTCTGTCTCGTAGGAG	abT	*	TRAV13-2	*	TRAJ13	*	*	*	CAETGGYQKVTF	
1195_AAGGTAACACACGGTC	abT	*	TRAV16	*	TRAJ22	*	*	*	CALTAGSARQLTF	
1224_CCTCAGTAGGTCGACA	abT	*	TRAV17	*	TRAJ30	*	*	*	CATDIALNRDDKIIF	
1066_CTGCATCCAATAGAGT	abT	*	TRAV17	*	TRAJ42	*	*	*	CAPYGGSQGNLIF	
1224_GCAGGCTCATGAGATA	abT	*	TRAV19	*	TRAJ17	*	*	*	CALSEALGKAAGNKLTF	
1235_GTACAACAGGAGAATG	abT	*	TRAV19	*	TRAJ39	*	*	*	CALSEVPNNAGNMLTF	
1416_TTTATGCGTGCCCTTT	abT	*	TRAV19	*	TRAJ50	*	TRAC	*	CALKTSYDKVIF	
898_CATCCACCAGACTCTA	abT	*	TRAV19	*	TRAJ7	*	*	*	CALSEPYGNNRLAF	
801_GAAACCTTCGTGAGAG	abT	*	TRAV20	*	TRAJ42	*	*	*	CAVEGGSQGNLIF	
1028_CTAGACAAGGTAGTAT	abT	*	TRAV22	*	TRAJ57	*	*	*	CAAEGGSEKLVF	
1195_CTGATCCCAAGGTCTT	abT	*	TRAV23DV6	*	TRAJ31	*	*	*	CAASGNARLMF	
1238_CAGGCCACAAAGACTA	abT	*	TRAV26-2	*	TRAJ53	*	TRAC	*	CILLSGSNYKLTF	
996_TGAGTCAAGGTCGTAG	abT	*	TRAV29DV5	*	TRAJ33	*	*	*	CAADSNYQLIW	
1355_ACCCACTAGTATGACA	abT	*	TRAV29DV5	*	TRAJ47	*	*	*	CAPKREYGNKLVF	
925_AAACCCAGTGTTCCAA	abT	*	TRAV3	*	TRAJ22	*	*	*	CAVRVPSGSARQLTF	
925_CCTAAGATCGAGATGG	abT	*	TRAV34	*	TRAJ47	*	*	*	CGASLEEYGNKLVF	
801_AAACGCTTCGGCCCAA	abT	*	TRAV36DV7	*	TRAJ54	*	*	*	CAVEAF	
996_GTGATGTAGGTGATAT	abT	*	TRAV38-2DV8	*	TRAJ22	*	TRAC	*	CAYRRTFSGSARQLTF	
1224_CTAACTTGTCACTGAT	abT	*	TRAV38-2DV8	*	TRAJ47	*	*	*	CAYIWEYGNKLVF	
1416_GTGTGATCACACGCCA	abT	*	TRAV38-2DV8	*	TRAJ49	*	*	*	CAYSGNQFYF	
925_GGCTTGGGTTTCACTT	abT	*	TRAV38-2DV8	*	TRAJ57	*	*	*	CAYRSDPQGGSEKLVF	
1130_ATTCTACCACCAGCTG	abT	*	TRAV39	*	TRAJ30	*	*	*	CAVGMNRDDKIIF	
925_GTCATCCCAGCACCCA	abT	*	TRAV39	*	TRAJ54	*	*	*	CAVEKGGAQKLVF	
943_CCCTTAGGTTCTGACA	abT	*	TRAV41	*	TRAJ42	*	*	*	CALNYGGSQGNLIF	
966_AACAAAGAGCTTTCTT	abT	*	TRAV5	*	TRAJ8	*	*	*	CAEIPDTGFQKLVF	
1238_CCCTCAATCGGCATAT	abT	*	TRAV9-2	*	TRAJ37	*	*	*	CALSLSSSNTGKLIF	
1238_CTGTATTGTCCGGACT	abT	*	TRAV9-2	*	TRAJ42	*	*	*	CALSPFGGSQGNLIF	
1195_CCTCACAAGGATATGT	gdT	TRDV2	*	TRDJ3	*	TRDC	*	CACDTLGDTSWDTRQMFF	*	
1238_GGGCTACAGTAGTGCG	gdT	TRDV2	*	TRDJ1	*	TRDC	*	CACDNLGGSTDKLIF	*	
1397_TGCGATACAGAACGCA	gdT	TRDV1	*	TRDJ1	*	TRDC	*	CALGDRQAFLRSSKPPYWGPHTDKLIF	*	
1416_CGACAGCAGATGGGCT	gdT	TRDV2	*	TRDJ1	*	TRDC	*	CACDTVVKDTTDKLIF	*	
1416_GTCTTTAGTTTCAGAC	gdT	TRDV2	*	TRDJ1	*	*	*	CACDRLPSGGYTDKLIF	*	
943_GTTATGGCAGCCTATA	gdT	TRDV2	*	TRDJ3	*	TRDC	*	CDTVLGDLSSWDTRQMFF	*	
1125_GGTAGAGGTTTCCCAC	gdT	TRDV2	*	TRDJ3	TRDC	*	*	CACDGLGDIPPRDSWDTRQMFF	*	
1066_GGTAGAGCACGACTAT	gdT	*	TRGV10	*	TRGJ2	*	TRGC1	*	CAAWVCWNYYKKLF	
1167_AAGTTCGGTCCTTTGC	gdT	*	TRGV10	*	TRGJ2	*	*	*	CAASGNYKKLF	
1195_TAGAGTCAGCGGTAAC	gdT	*	TRGV10	*	TRGJ2	*	TRGC2	*	CAAFYYYKKLF	
1224_AGTCATGCAATTGTGC	gdT	*	TRGV10	*	TRGJ2	*	*	*	CAAWDYWPKLF	
1224_CATACAGGTGGACCAA	gdT	*	TRGV10	*	TRGJ2	*	TRGC2	*	CAAWDYNYKKLF	
1238_AATCACGTCCATAGGT	gdT	*	TRGV10	*	TRGJ2	*	*	*	CAAWDANYYKKLF	
1238_GACGCTGTCCGCTTAC	gdT	*	TRGV10	*	TRGJ2	*	TRGC2	*	CAAWEHYKKLF	
1355_TGGCCAGTCTTCGGTC	gdT	*	TRGV10	*	TRGJ2	*	*	*	CAASGIEKLF	
1130_CCTCCAATCCTACCGT	gdT	*	TRGV10	*	TRGJP1	*	*	*	CAAWEYYTRRDTTGWFKIF	
1416_AATGCCAGTACAGCGA	gdT	TRDV2	TRGV10	TRDJ3	TRGJP1	TRDC	TRGC1	CACDKMLGDNSWDTRQMFF	CATFRLRATTGWFKIF	
1224_GTCTCACTCACACCCT	gdT	*	TRGV2	*	TRGJ2	*	*	*	CATWDGPEGDYKKLF	
1224_GTGAGCCGTAGTAAGT	gdT	*	TRGV2	*	TRGJ2	*	*	*	CATWDDFSYYKKLF	
1238_ACGATGTTCACGAACT	gdT	*	TRGV2	*	TRGJ2	*	TRGC2	*	CATWDGLYYKKLF	
1238_TGTTACTGTTCCTAAG	gdT	*	TRGV2	*	TRGJ2	*	TRGC1	*	CATWEGGYKKLF	
1238_TGTTACTTCTGAACGT	gdT	*	TRGV2	*	TRGJ2	*	TRGC2	*	CATWDGQEKLF	
925_AGGCCACGTACTGACT	gdT	*	TRGV2	*	TRGJ2	*	*	*	CATWDVPPWKKLF	
925_TAGGTACAGACATATG	gdT	*	TRGV2	*	TRGJ2	*	*	*	CATWDIYYKKLF	
925_TGCTTCGAGATCGCCC	gdT	*	TRGV2	*	TRGJ2	*	*	*	CATWDELGKKLF	
945_GAGCTGCGTTATGGTC	gdT	*	TRGV2	*	TRGJ2	*	TRGC2	*	CATWDGPTQESYYMKLF	
1167_ATTTACCCAAATGATG	gdT	*	TRGV3	*	TRGJ2	*	TRGC2	*	CATWDSLYKKLF	
1235_ATGAAAGCAGAGGTAC	gdT	*	TRGV3	*	TRGJ2	*	*	*	CATLHYYKKLF	
1238_AGAGCCCTCACAATGC	gdT	*	TRGV3	*	TRGJ2	*	*	*	CHLGQGPLIFYYKKLF	
966-2_CCGTGAGAGAGGGTAA	gdT	*	TRGV3	*	TRGJ2	*	TRGC1	*	CATWDRPRYYKKLF	
1235_TCAAGCAGTAGACTGG	gdT	*	TRGV3	*	TRGJP2	*	*	*	CATWDRLPDWIKTF	
1125_CTAAGTGTCTACGCAA	gdT	*	TRGV4	*	TRGJ2	*	*	*	CATPNNKLF	
1224_ACGTCCTTCATTATCC	gdT	*	TRGV4	*	TRGJ2	*	TRGC2	*	CATWDPSRNYYKKLF	
925_GTCCTCATCCATGATG	gdT	*	TRGV4	*	TRGJ2	*	*	*	CATRYYYKKLF	
934_GTGACGCCAATGAACA	gdT	*	TRGV4	*	TRGJ2	*	*	*	CAPPVKKLF	
966_TCAGTTTTCGCCAGAC	gdT	*	TRGV5	*	TRGJ2	*	*	*	CATWDRRYYKKLF	
1224_AGCATCAGTGGCTTAT	gdT	*	TRGV7	*	TRGJ2	*	*	*	CATWDKAKLF	
1224_CATGGTAAGCTGAAAT	gdT	*	TRGV8	*	TRGJ2	*	TRGC2	*	CATWDRWYKKLF	
1235_TCAGTGATCCTTATAC	gdT	*	TRGV8	*	TRGJ2	*	*	*	CATWAMHPNFYYKKLF	
1238_CGGGCATAGCACCGTC	gdT	*	TRGV8	*	TRGJ2	*	*	*	CATWDMRGYKKLF	
1066_GCCGTGACAATCGTCA	gdT	*	TRGV9	*	TRGJ2	*	TRGC2	*	CALWGRWDKKLF	
1195_ACATTTCAGGGAGGAC	gdT	*	TRGV9	*	TRGJ2	*	*	*	CALWEHQNYYKKLF	
1235_TATACCTTCTCACCCA	gdT	*	TRGV9	*	TRGJ2	*	*	*	CALWTMNYYKKLF	
1238_AGTTAGCTCTTAGCTT	gdT	*	TRGV9	*	TRGJ2	*	*	*	CALWEDYYKKLF	
801_GCTGGGTCAAAGACTA	gdT	*	TRGV9	*	TRGJ2	*	*	*	CALWEFSNYYKKLF	
945_GAAACCTTCACACCCT	gdT	TRDV1	TRGV9	TRDJ1	TRGJ2	TRDC	TRGC2	CALGGGSVGGYGDKLIF	CAFQPNTSYYKKLF	
996_CTCATGCGTGGACAGT	gdT	*	TRGV9	*	TRGJ2	*	TRGC2	*	CALSISASYYKKLF	
898_GCAACATCAAACCACT	gdT	TRDV2	TRGV9	TRDJ3	TRGJP	TRDC	TRGC1	CACDTLLGDTWDTRQMFF	CALWEVQELGKKIKVF	blood clonotype
996_TATTCCAAGGACGCTA	gdT	*	TRGV9	*	TRGJP	*	TRGC1	*	CALWGNKSWAKKIKVF	

Normalized gene expression

Barcode	cell_type	XCL1	SELL	CCL20	PDCD1	CXCR6	CCR2	CCR5	TIGIT	LEF1	IL7R	ITGA1	GZMK	GZMA	TCF7	HAVCR2	CXCR3	TOX	PRF1	ENTPD1	MKI67	LAG3	CD69	KLRC1	IFNG	GZMB	ITGAE	CCL5	CCL3	CCR7	TNFSF9
1066_GTGAGGACACAAGGTG	abT	0	0	0,546758	0,898243	1,157802	0,546758	0	2,197677	0	0	1,363684	0	5,989014	0	1,157802	1,363684	0,546758	2,113412	1,920027	0	3,677947	1,680056	0	1,807223	0,546758	0,546758	3,404715	4,376832	0	0,546758
1195_AGAAATGGTGGTGATG	abT	0	0	1,312228	0,857483	0	0	0	1,860801	0	1,860801	0	0	0	0	0	0	0	0	0,857483	0	0	0	0	0	0	0,857483	0	0	0	0
1224_CGAAGTTGTAGCACAG	abT	0	0	0	1,970125	1,632056	0	1,117496	1,117496	0	0	0	3,162478	3,834238	0	0	1,117496	0	0	0	0	0	1,632056	0	0	1,632056	0	3,959474	0	0	0
1224_GGGATCCCACAGAGCA	abT	0	1,142606	0	0	1,142606	0	0	0	0	0	0	2,002126	3,006392	0	0	0	0	1,142606	0	0	0	2,255463	0	0	0	0	1,662003	0	0	0
1238_AATGGCTGTCACTCTC	abT	0	0	0	1,400946	0	0	0	0	0	1,400946	0	2,962911	2,582952	1,400946	0	0	1,400946	0	0	0	0	1,400946	0	0	0	0	3,237592	0	1,400946	0
1238_ACCAACAGTATTTCTC	abT	0	1,366017	0	1,366017	0	0	0	0	0	4,033788	1,366017	0	1,922702	0	0	0	0	0	0	0	0	2,278203	0	0	0	0	1,366017	0	0	0
1238_AGGAATAGTCACTTCC	abT	0	0	0	0	0	1,48534	0	0	0	0	0	3,560042	2,420315	0	0	0	0	1,48534	0	0	0	1,48534	0	2,058337	0	0	2,894936	0	1,48534	1,48534
1238_GATCACATCCACCTCA	abT	1,093307	0	0	0	1,093307	0	0	1,093307	0	0	1,603068	2,700615	3,603166	0	0	1,093307	1,093307	1,603068	0	1,093307	1,603068	1,093307	0	0	0	1,093307	4,664936	0	0	0
1238_TCCATGCGTAGCTGTT	abT	0	0	0	1,655461	0	0	0	0	0	0	0	2,887143	3,273855	0	0	2,248233	0	0	0	0	0	0	0	0	0	0	3,862632	0	0	0
1416_CAACCAAAGGACATCG	abT	0	0	0	0	2,937568	1,024962	0	0	0	0	0	0	0	1,024962	0	0	0	1,024962	0	0	0	1,850181	0	0	0	0	0	0	0	0
925_CTTGATTGTTCTCCAC	abT	0	0	0	0	0	0	1,874918	1,324457	1,324457	0	0	2,865673	2,488282	1,324457	0	1,874918	1,324457	0	0	0	0	2,694676	0	0	0	1,324457	3,529934	1,324457	0	0
925_GCCGTGACAAGTGGTG	abT	0	0	0	0	0	0	0	0	1,134671	1,652555	0	0	0	0	0	0	0	0	0	0	0	0	0	0	0	0	0	0	0	0
925_TATTTCGTCGTGTCAA	abT	0	0	0	0	1,381886	0	0	0	0	3,085428	0	1,94087	0	0	0	1,381886	0	0	0	0	0	1,381886	0	0	0	1,381886	3,755455	0	0	0
943_AGTAACCCACCGCTGA	abT	0	0	0	0	1,311435	0	0	0	0	0	0	2,471989	1,311435	0	0	1,311435	0	0	0	0	0	1,859884	0	1,311435	0	0	0	0	0	0
943_CCTCCAAGTGACTATC	abT	0	0	0	0	0	0	0	0	0	0	0	1,49627	1,49627	0	0	0	0	1,49627	0	0	0	3,081452	0	0	0	0	2,070654	0	0	0
898_CATCCGTTCTCATTTG	abT	0	0	0	0	0	0	0	0	0	0	0	2,9218	3,737851	0	1,925449	1,925449	1,368414	2,28109	0	0	0	2,750188	2,28109	0	2,28109	0	4,4537	0	0	0
996_TACAACGAGCGCTTCG	abT	0	1,484284	0	0	0	0	0	0	2,419071	3,342665	0	0	0	0	0	0	0	0	0	0	0	1,484284	0	0	0	0	0	0	2,419071	0
1238_GGTAATCCATAATGCC	abT	0	0	0	0	0	0	0	0	0	0	0	3,082246	3,752198	0	1,64122	0	0	1,64122	0	0	0	0	0	1,64122	0	1,64122	4,081943	0	0	0
925_GCCATGGCACAGTGAG	abT	0	0	0	0	0	0	0	0	0	4,221184	0	2,500262	0	0	0	0	0	0	0	0	2,500262	2,500262	0	0	0	0	0	0	0	0
1224_TTTCAGTAGGGACCAT	abT	0	0	0	1,272575	0	0	0	0	0	0	0	0	2,943925	0	0	1,272575	0	1,814836	0	0	1,272575	0	0	0	2,798512	1,272575	3,958919	1,272575	0	0
1238_GAGACTTCAACACAGG	abT	0	0	0	1,563098	2,989107	0	0	0	0	0	2,1456	3,311094	2,1456	0	0	1,563098	1,563098	0	0	0	0	0	0	1,563098	0	0	3,913154	1,563098	0	0
1238_GTAGAGGAGTGAGCCA	abT	0	0	0	0	0	0	0	0	0	0	1,836234	3,095192	3,485454	0	0	0	0	0	0	0	1,291004	1,291004	0	0	0	1,291004	4,535555	1,291004	1,291004	0
831_TACAACGTCAGCTCTC	abT	0	0	0	0	1,124465	1,124465	0	0	1,124465	0	2,74406	2,231549	1,979028	1,124465	0	1,124465	0	1,640382	0	0	1,640382	0	0	0	1,640382	1,124465	3,5928	0	0	0
1238_GCAGCCATCAGACATC	abT	0	1,318611	0	0	2,480974	0	1,318611	0	0	0	0	2,858156	3,244311	0	0	0	1,318611	0	0	0	1,318611	1,318611	0	0	0	1,318611	3,739415	0	1,868173	0
934_GATGGAGGTAACGGTG	abT	0	0	0	0	0	0	0	0	0	3,636502	0	2,188267	2,823727	1,837343	1,29196	1,29196	0	0	0	0	0	1,29196	0	0	0	0	2,653212	0	0	0
898_TACGCTCCAATAGGGC	abT	0	0	0	0	0	0	0	0	0	3,213516	0	0	1,841126	0	1,295225	0	0	0	0	0	0	0	0	0	0	0	1,295225	0	0	0
1130_AACCCAATCACTTTGT	abT	0	0	0	0	0	0	1,528817	0	0	0	2,107231	2,107231	2,737665	0	0	1,528817	0	0	0	0	0	0	0	0	0	0	4,388006	0	0	0
925_GCCCGAAGTTGTCCCT	abT	0	0	0	0	0	0	0	0	0	3,954978	0	0	0	2,254699	0	0	0	0	0	0	0	2,254699	0	0	0	0	0	0	0	0
1238_GTGAGCCTCGGTAACT	abT	0	0	0	0	0	0	0	0	0	0	2,236201	3,849454	3,538953	0	0	0	0	0	0	0	0	0	0	0	0	0	4,333616	0	0	0
1224_GTCTGTCTCGTAGGAG	abT	0	1,400025	0	0,92729	0	0	0	0	1,719718	1,961587	0	0	0	1,400025	0	0	1,400025	0	0	0	0	1,961587	0	0	0	0	0	0	0,92729	0
1195_AAGGTAACACACGGTC	abT	0	0	0	0	0	0	0	0	0	0	0	0	1,994592	0	0	0	0	0	0	0	0	0	0	0	0	1,429019	1,429019	0	0	0
1224_CCTCAGTAGGTCGACA	abT	0	0	0	0	0	0	0	0	0	0	2,938584	0	0	2,297024	0	0	0	0	0	0	0	0	0	0	0	0	2,297024	0	0	0
1066_CTGCATCCAATAGAGT	abT	0	1,443925	0	0	0	0	0	0	0	2,63542	0	1,443925	2,512078	1,767457	0	0,962658	0	0	0	0	0	0,962658	0	0	0	0	0	0	0	0
1224_GCAGGCTCATGAGATA	abT	0	0	0	0	1,827496	0	0	0	1,007751	2,812704	0	2,271834	2,912042	0	0	1,827496	0	0	0	0	0	2,578399	0	0	0	0	3,085262	0	0	0
1235_GTACAACAGGAGAATG	abT	0	0	0	0	0	0	0	0	0	0	0	3,459691	0	0	0	0	0	1,96871	0	0	0	0	0	0	0	0	4,31665	0	0	0
1416_TTTATGCGTGCCCTTT	abT	0	0	0	0	2,206283	0	1,306701	0	0	0	1,85441	1,306701	3,330047	0	0	0	0	0	0	0	0	1,306701	0	0	0	0	2,466052	0	0	0
898_CATCCACCAGACTCTA	abT	0	0,970123	0	0	0	1,453144	0,970123	0	0	2,021955	0	1,453144	3,028163	0,970123	0	0	0,970123	0	0	0	0	0	0	0	0	0	3,028163	0	0	0,970123
801_GAAACCTTCGTGAGAG	abT	0	0,660161	1,556125	0	0	0,660161	0	0	0	2,709819	0	0	2,020981	0	0,660161	0	0	0	0	0	0	2,503176	0	0	0	0,660161	0	0	0,660161	0
1028_CTAGACAAGGTAGTAT	abT	0	0	0	0	0	0	0	0	0	3,848309	0	0	3,848309	0	0	0	0	0	0	0	0	2,320694	0	0	0	0	2,963492	0	0	0
1195_CTGATCCCAAGGTCTT	abT	0	0	0	0	1,774471	0	0	0	0	2,583462	0	0	2,753118	1,237962	0	0	0	1,237962	0	0	0	1,774471	0	0	0	0	2,583462	0	0	0
1238_CAGGCCACAAAGACTA	abT	0	0	0	0	0	0	0	1,22355	0	1,757593	0	0	0	0	0	0	0	0	0	0	0	0	0	0	0	0	0	0	0	0
996_TGAGTCAAGGTCGTAG	abT	0	0	0	0,99158	0	0	0	1,806068	0	0	0	2,413495	0,99158	0	1,479552	0	0	0,99158	0	0	0	1,479552	0	0	0,99158	0	3,33676	0	0	0
1355_ACCCACTAGTATGACA	abT	0	0	0	0	0	0	0	0	0	0	0	0	0	0	0	0	0	0	0	0	0	0	0	0	0	0	4,03386	0	0	0
925_AAACCCAGTGTTCCAA	abT	0	0	0	0	1,415411	0	0	0	0	2,600667	1,415411	2,981079	1,979118	0	1,979118	0	0	0	0	0	0	0	0	0	0	0	3,563882	0	0	0
925_CCTAAGATCGAGATGG	abT	0	0	0	0	0	0	0	0	0	0	0	3,643784	3,399914	0	0	0	0	2,227293	0	0	1,636539	2,227293	0	0	0	1,636539	4,076232	0	0	0
801_AAACGCTTCGGCCCAA	abT	0	0	0	1,347603	0	0	0	0	0	0	1,347603	2,517113	3,475999	0	0	0	1,347603	0	0	0	0	0	0	1,901567	0	1,347603	4,355441	0	1,347603	0
996_GTGATGTAGGTGATAT	abT	0	1,053167	0	0	0	0	0	1,053167	1,053167	1,554651	0	0	0	1,053167	0	0	0	1,053167	1,053167	0	0	0	0	0	0	1,053167	0	0	0	0
1224_CTAACTTGTCACTGAT	abT	0	2,051513	0	0	0	0	0	0	0	2,788354	0	0	0	1,805785	0	0	0	0	0	0	0	1,805785	0	0	0	0,991367	0	0	2,554464	0
1416_GTGTGATCACACGCCA	abT	0	0	0	0	1,546603	1,046528	1,046528	0	0	0	0	1,546603	3,219703	0	0	1,046528	0	0	0	0	0	1,046528	0	0	0	1,046528	3,142986	0	0	0
925_GGCTTGGGTTTCACTT	abT	0	0	0	0	0	0	0	0	0	0	0	0	0	0	0	0	0	0	0	0	0	3,105571	0	3,105571	0	0	0	0	0	0
1130_ATTCTACCACCAGCTG	abT	1,588336	0	0	1,588336	0	0	0	2,173751	0	0	0	3,341772	1,588336	0	0	0	0	1,588336	0	0	0	2,540562	0	0	0	0	3,585195	2,173751	0	1,588336
925_GTCATCCCAGCACCCA	abT	0	0	0	1,173145	0	0	0	0	0	2,498211	0	0	2,040756	1,698233	1,173145	2,040756	0	0	0	0	0	2,810908	0	0	0	1,173145	2,498211	0	0	0
943_CCCTTAGGTTCTGACA	abT	0	0	0	0	0	0	0	0	0	2,433413	0	0	0	1,280775	0	0	0	0	0	0	0	2,174557	0	0	0	0	0	0	0	0
966_AACAAAGAGCTTTCTT	abT	0	0	0	0	0	0	0	0	0	0	0	3,01824	3,904759	0	0	0	0	1,445163	0	0	0	0	0	0	1,445163	2,012912	4,235459	0	0	0
1238_CCCTCAATCGGCATAT	abT	0	0	0	0	1,985324	0	1,985324	0	0	0	0	1,985324	0	0	0	0	0	0	1,985324	0	0	0	0	0	0	0	1,985324	0	1,985324	0
1238_CTGTATTGTCCGGACT	abT	0	0	0	0	0	1,455408	0	0	0	0	0	3,777828	1,455408	0	0	1,455408	1,455408	0	0	0	0	0	0	0	0	0	3,777828	0	0	0
1195_CCTCACAAGGATATGT	gdT	0	0	0	0	0,967587	0	0	0	0	3,843411	0	2,214621	2,214621	1,774064	0	0	0	1,774064	0	0	0	2,378576	0	0	0	0	3,171458	0	0	0
1238_GGGCTACAGTAGTGCG	gdT	0	0	0	0	1,431438	0	1,431438	0	0	2,356518	0	3,148153	1,431438	0	0	1,431438	0	0	0	0	0	2,356518	1,431438	0	0	2,356518	3,148153	0	0	0
1397_TGCGATACAGAACGCA	gdT	0	0	0	0,831026	0	0	0	1,278527	0	0	0	2,430572	3,339645	0	0	0,831026	0,831026	1,278527	0	0	0,831026	0	0	0	0,831026	0,831026	3,685628	0	0	0
1416_CGACAGCAGATGGGCT	gdT	1,250624	0	0	0	2,914937	0	1,250624	0	0	1,789264	0	0	3,255216	0	0	1,789264	1,250624	0	0	0	0	0	2,137424	0	1,250624	0	3,431088	0	0	0
1416_GTCTTTAGTTTCAGAC	gdT	0	0	0	0	1,997523	0	1,138984	0	0	1,138984	0	0	2,764125	0	0	0	0	1,657692	0	0	0	1,138984	0	0	0	1,138984	2,250699	0	0	0
943_GTTATGGCAGCCTATA	gdT	0	0	0	0	0	0	0	0	0	1,920131	0	0	2,370532	0	0	0	0	1,078619	0	0	1,078619	0	1,585398	0	1,078619	0	3,777201	0	0	0
1125_GGTAGAGGTTTCCCAC	gdT	0	0	0	0	0	0	0	0	0	4,119866	1,953751	3,227654	1,393158	0	0	1,393158	0	2,310816	0	0	0	2,310816	0	0	1,393158	1,393158	2,781162	0	0	0
1066_GGTAGAGCACGACTAT	gdT	0	0	0	0	0	0	0	0	0	2,323805	1,195018	1,724057	2,966763	0	0	0	0	2,068235	0	0	1,195018	2,840558	1,195018	0	0	0	2,323805	0	0	0
1167_AAGTTCGGTCCTTTGC	gdT	0	0	0	0	0	0	1,844929	0	0	0	0	2,455761	3,105062	0	0	0	1,844929	0	0	0	0	0	0	0	0	0	1,844929	0	0	0
1195_TAGAGTCAGCGGTAAC	gdT	0	0	0	0	1,428537	0	0	0	0	0	1,428537	2,825119	2,616692	0	1,428537	1,994044	0	1,994044	0	0	1,428537	1,994044	0	0	2,616692	1,428537	3,580695	0	0	0
1224_AGTCATGCAATTGTGC	gdT	0	0	0	0	0	0	0	0	0	0	1,341104	3,769283	2,88701	0	0	0	1,341104	0	0	0	1,341104	3,375286	0	0	0	1,341104	4,099142	0	0	0
1224_CATACAGGTGGACCAA	gdT	0	0	0	1,199373	0	0	0	0	0	2,846434	0	2,701912	3,438315	1,199373	0	0	0	1,199373	0	0	0	0	0	0	1,729187	0	3,951181	0	0	0
1238_AATCACGTCCATAGGT	gdT	0	0	0	1,823276	1,823276	0	1,823276	0	0	0	2,432228	2,432228	2,432228	0	0	0	0	1,823276	0	0	3,080461	1,823276	0	0	0	0	4,491619	0	0	0
1238_GACGCTGTCCGCTTAC	gdT	0	0	0	0	0	0	0	0	0	0	0	3,481501	2,818651	0	0	0	1,597073	0	0	0	0	2,550669	0	0	0	0	3,791529	1,597073	0	0
1355_TGGCCAGTCTTCGGTC	gdT	0	0	0	0	2,085326	0	0	0	0	0	2,085326	2,714326	3,373787	0	0	0	0	2,085326	0	0	0	2,714326	2,085326	0	0	0	3,918612	0	0	0
1130_CCTCCAATCCTACCGT	gdT	1,336329	1,336329	0	0	0	0	0	0	1,336329	3,885388	0	1,888599	2,50309	0	1,336329	1,336329	0	1,888599	0	0	0	1,336329	0	0	2,50309	0	3,154461	0	0	0
1416_AATGCCAGTACAGCGA	gdT	1,18879	0	0	0	2,31574	0	0	0	0	0	0	0	3,562368	0	0	0	0	0	0	0	1,18879	0	2,060426	0	0	0	2,958282	0	0	0
1224_GTCTCACTCACACCCT	gdT	0	0,941269	0	0	0	0	0	0	0	0	0	0	2,480114	0	0	0,941269	0	0,941269	0	0	0	0,941269	0	0	0	0	4,447517	0	0	0
1224_GTGAGCCGTAGTAAGT	gdT	0	0	0	0	0	0	0	0	0	0	0	0	1,381886	0	0	0	0	1,381886	0	0	0	0	0	0	2,55952	0	3,428295	0	0	0
1238_ACGATGTTCACGAACT	gdT	0	0	0	0	1,65529	0	0	0	0	1,136967	1,65529	2,248044	2,248044	1,136967	0	1,136967	0	0	0	0	0	0	0	0	0	0	3,551821	1,65529	0	0
1238_TGTTACTGTTCCTAAG	gdT	0	0	0	0	0	0	0	0	0	0	0	1,606564	3,493023	0	0	0	0	1,606564	0	0	0	1,606564	0	0	0	0	4,286915	0	0	0
1238_TGTTACTTCTGAACGT	gdT	0	0	0	1,524025	1,524025	0	0	1,524025	0	0	0	2,465723	2,941979	0	0	0	1,524025	2,465723	0	0	0	0	0	1,524025	0	0	3,506037	0	0	0
925_AGGCCACGTACTGACT	gdT	0	0	0	1,015435	1,015435	0	0	1,015435	1,015435	1,508761	2,084709	2,28267	1,508761	0	0	1,015435	0	0	0	0	0	1,015435	1,837639	0	0	1,015435	3,310851	0	0	0
925_TAGGTACAGACATATG	gdT	1,227629	0	0	0	0	0	0	0	0	2,884348	1,227629	2,569986	0	0	0	0	0	0	0	0	1,762374	0	0	0	0	1,762374	2,884348	0	0	0
925_TGCTTCGAGATCGCCC	gdT	0	0	0	0	0	0	0	1,105729	0	1,105729	1,105729	3,14569	3,054674	0	0	1,105729	0	1,617972	0	0	0	1,955051	1,617972	0	0	0	3,565702	1,105729	0	0
945_GAGCTGCGTTATGGTC	gdT	0	0	0	0	0	0	0	0	0	3,992089	0	2,659698	1,692061	0	0	0	0	1,692061	0	0	1,692061	1,692061	0	0	0	0	0	0	0	0
1167_ATTTACCCAAATGATG	gdT	0	0	0	0	0	0	1,566387	0	0	0	0	2,149274	2,515108	0	0	0	0	1,566387	0	0	0	2,149274	0	0	0	0	2,515108	0	0	0
1235_ATGAAAGCAGAGGTAC	gdT	0	0	0	1,673868	0	0	0	0	0	0	0	3,295708	2,268564	0	0	0	0	0	0	0	0	0	0	0	0	0	4,189523	0	0	0
1238_AGAGCCCTCACAATGC	gdT	0	0	0	0	0	0	0	0	0	2,897845	0	2,061024	0	0	0	0	0	0	0	0	0	0	0	0	0	1,487723	3,460819	1,487723	0	0
966-2_CCGTGAGAGAGGGTAA	gdT	0	1,604655	0	0	0	0	0	0	2,191909	3,605098	0	0	0	2,191909	0	0	0	0	0	0	0	0	0	0	0	0	2,191909	0	0	0
1235_TCAAGCAGTAGACTGG	gdT	0	0	0	0	0	0	0	1,009078	1,009078	2,438725	0	0	0	1,82925	0	1,009078	0	0	0	0	0	1,500993	0	0	0	1,500993	3,163783	0	1,500993	0
1125_CTAAGTGTCTACGCAA	gdT	0	0	0	0	0	0	0	0	0	3,348498	0	0	1,71859	1,71859	0	0	0	2,317801	0	0	0	1,71859	0	0	1,71859	0	2,689885	0	0	0
1224_ACGTCCTTCATTATCC	gdT	1,049655	0	0	1,049655	0	0	0	1,550395	1,049655	0	0	3,224327	2,973827	0	0	1,049655	0	0	0	0	0	1,049655	0	0	0	0	4,217151	0	0	0
925_GTCCTCATCCATGATG	gdT	0	0	0	0	0	0	0	0	0	2,564211	0	0	0	0	0	0	0	0	0	0	0	2,564211	0	0	0	2,564211	2,564211	0	0	0
934_GTGACGCCAATGAACA	gdT	0	1,328524	0	0	0	0	1,328524	1,328524	0	1,328524	0	1,328524	2,493359	0	0	0	1,328524	0	0	0	0	1,328524	1,328524	0	2,493359	1,328524	3,984043	0	0	0
966_TCAGTTTTCGCCAGAC	gdT	2,843817	0	0	0	0	0	0	0	0	3,229691	0	0	0	2,207242	0	0	0	0	0	0	0	0	0	0	0	0	0	0	0	0
1224_AGCATCAGTGGCTTAT	gdT	0	0	0	0	0	0	0	0	0	0	0	0	2,089348	0	1,512888	0	0	2,718614	0	0	0	0	0	0	3,492208	0	3,59452	0	0	0
1224_CATGGTAAGCTGAAAT	gdT	0	0	0	0	0	0	0	0	0	0	0	0	2,604621	0	0	0	0	1,9828	0	0	0	1,127421	0	0	2,235455	0	2,873647	0	0	0
1235_TCAGTGATCCTTATAC	gdT	0	0	0	0	0	0	0	0	2,007035	3,96138	0	0	0	1,43998	0	1,43998	0	0	0	0	0	1,43998	0	0	0	0	2,630625	0	0	0
1238_CGGGCATAGCACCGTC	gdT	0	0	0	0	0	0	0	0	0	0	0	3,023436	2,177557	1,591755	0	0	1,591755	1,591755	0	0	0	2,544518	0	0	0	0	3,197619	0	0	0
1066_GCCGTGACAATCGTCA	gdT	0	0	0	0	0	0	0	0	0	3,517249	0	0	0	1,533075	0	1,533075	0	0	0	0	0	1,533075	0	0	0	0	2,952929	0	1,533075	0
1195_ACATTTCAGGGAGGAC	gdT	0	0	0	0	0	0	1,469679	2,401844	0	0	0	2,875777	2,875777	0	0	0	0	0	0	0	0	2,040659	0	0	0	0	4,058947	1,469679	0	0
1235_TATACCTTCTCACCCA	gdT	0	0	0	0	0	0	0	0	1,726712	3,435403	0	2,8436	0	1,726712	0	1,726712	0	0	0	0	0	1,197271	0	0	0	0	2,699105	0	1,197271	0
1238_AGTTAGCTCTTAGCTT	gdT	0	0	0	0	1,629578	0	0	0	0	0	1,629578	3,391553	3,242996	0	0	0	1,629578	0	0	0	0	0	0	2,219578	0	0	3,916427	0	0	0
801_GCTGGGTCAAAGACTA	gdT	0	0	0	0	0	0	0	0	1,617162	1,617162	0	0	0	0	0	0	0	0	0	0	0	0	0	0	0	0	0	0	0	0
945_GAAACCTTCACACCCT	gdT	0	0	0	2,024613	0	1,683083	0	0	0	0	2,024613	2,649457	3,383841	0	0	0	1,160353	0	0	0	0	1,160353	0	0	0	0	3,522409	0	0	0
996_CTCATGCGTGGACAGT	gdT	0	0	0	0	0	0	1,5014	0	0	0	2,439203	1,5014	3,580171	0	0	0	0	1,5014	0	0	0	2,439203	0	0	0	0	4,518382	0	0	0
898_GCAACATCAAACCACT	gdT	0	0	0	0	0	0	0	0	0	0	0	2,188736	3,209706	0	0	1,601802	0	0	0	0	0	2,188736	0	0	0	0	3,704252	0	0	0
996_TATTCCAAGGACGCTA	gdT	1,718715	0	0	0,926552	0	0	0	0	0	1,960537	0	0,926552	2,457921	0,926552	0	0,926552	0	0	0	0	0	0	0	0	0	0	3,667264	0	0	0

**Score**						
**Barcode**	**cell_type**	**Exhausted**	**pre-exhausted**	**Naive**	**effector-memory**	**Resident**
1066_GTGAGGACACAAGGTG	abT	1,334852413	-0,122349977	-0,535360187	1,087109179	0,368755254
1195_AGAAATGGTGGTGATG	abT	-0,077947844	-0,455787645	-0,50754744	-0,224078057	-0,312617886
1224_CGAAGTTGTAGCACAG	abT	0,241103127	-0,272733524	-0,354436711	0,835285504	0,311628506
1224_GGGATCCCACAGAGCA	abT	-0,259803944	-0,257092772	-0,082070968	0,343240804	-0,17177495
1238_AATGGCTGTCACTCTC	abT	-0,147811718	0,452933281	0,423195909	0,660618037	-0,279127702
1238_ACCAACAGTATTTCTC	abT	-0,212463374	-0,280450205	0,006204369	0,449249549	-0,108392291
1238_AGGAATAGTCACTTCC	abT	0,079724165	-0,223588732	0,045513061	0,79061959	-0,04214554
1238_GATCACATCCACCTCA	abT	0,040639343	0,752838881	-0,478491569	0,613925907	0,381181693
1238_TCCATGCGTAGCTGTT	abT	-0,049071853	-0,156650597	-0,234194993	0,554547457	0,157645823
1416_CAACCAAAGGACATCG	abT	-0,372028979	-0,359980675	-0,213541372	-0,345183055	0,252777885
925_CTTGATTGTTCTCCAC	abT	-0,186337718	0,462869554	0,396032974	0,766763166	0,558313482
925_GCCGTGACAAGTGGTG	abT	-0,353551432	-0,215606148	-0,001528039	-0,203026562	-0,261332527
925_TATTTCGTCGTGTCAA	abT	-0,159366401	-0,109278881	-0,124232469	0,643039356	0,523289582
943_AGTAACCCACCGCTGA	abT	-0,157705265	-0,166997318	-0,25818439	0,218129264	0,167776862
943_CCTCCAAGTGACTATC	abT	-0,10261222	-0,206833621	-0,229500465	0,325565428	-0,180029558
898_CATCCGTTCTCATTTG	abT	0,435410669	0,392391161	-0,36108976	1,174434174	0,00939746
996_TACAACGAGCGCTTCG	abT	-0,490557241	-0,272139263	1,215353137	0,024837037	-0,370158479
1238_GGTAATCCATAATGCC	abT	0,341791465	-0,219489341	-0,259576969	0,752049371	0,015602975
925_GCCATGGCACAGTGAG	abT	0,065795149	-0,104090255	-0,139377025	0,576374089	-0,12166068
1224_TTTCAGTAGGGACCAT	abT	0,564584583	-0,218726746	-0,316635827	0,586461861	0,134560474
1238_GAGACTTCAACACAGG	abT	0,024595688	0,578015431	-0,272975256	0,697392744	0,889047706
1238_GTAGAGGAGTGAGCCA	abT	-0,245328693	-0,228046622	-0,046071253	0,758432245	0,182055757
831_TACAACGTCAGCTCTC	abT	-0,072801485	-0,548543872	-0,141742385	0,147767569	0,571742968
1238_GCAGCCATCAGACATC	abT	-0,232270705	0,445626621	0,493717148	0,549330341	0,531326216
934_GATGGAGGTAACGGTG	abT	-0,071672131	-0,135789917	0,273321542	0,780172513	0,049387116
898_TACGCTCCAATAGGGC	abT	-0,064637169	-0,158331262	-0,210018861	0,252633033	-0,193685441
1130_AACCCAATCACTTTGT	abT	-0,343826277	-0,36021691	-0,399630373	0,387247751	0,541689174
925_GCCCGAAGTTGTCCCT	abT	-0,179879794	-0,121155528	0,407269009	0,406936563	-0,136254915
1238_GTGAGCCTCGGTAACT	abT	-0,305704338	-0,129573046	-0,260266353	0,676814988	0,184803858
1224_GTCTGTCTCGTAGGAG	abT	-0,326538858	0,381586997	0,981222606	-0,074879492	-0,346238526
1195_AAGGTAACACACGGTC	abT	-0,232793302	-0,19860204	-0,231954965	-0,009227872	0,046236249
1224_CCTCAGTAGGTCGACA	abT	-0,467845316	-0,337466111	0,164779392	-0,165787612	0,106837183
1066_CTGCATCCAATAGAGT	abT	-0,270560037	-0,158775761	0,591550799	0,314572148	-0,034611481
1224_GCAGGCTCATGAGATA	abT	-0,371272644	-0,231861782	-0,054827818	0,726687069	0,351805938
1235_GTACAACAGGAGAATG	abT	-0,016604617	-0,143207291	-0,20431776	0,403547435	-0,20916989
1416_TTTATGCGTGCCCTTT	abT	-0,339678596	-0,171945666	-0,269414561	0,331546499	0,64944993
898_CATCCACCAGACTCTA	abT	-0,507712512	0,093203261	0,004316436	0,381821424	-0,019950126
801_GAAACCTTCGTGAGAG	abT	-0,39215973	-0,324985398	-0,062752674	0,27527618	-0,124065734
1028_CTAGACAAGGTAGTAT	abT	-0,127706667	-0,166810844	-0,145416951	0,909204961	-0,140402877
1195_CTGATCCCAAGGTCTT	abT	-0,185052796	-0,250236614	-0,014903632	0,436470048	-0,012302691
1238_CAGGCCACAAAGACTA	abT	-0,254349656	-0,331220061	-0,39328093	-0,257118531	-0,400794672
996_TGAGTCAAGGTCGTAG	abT	0,440836395	-0,255952401	-0,361812774	0,437901673	-0,350855104
1355_ACCCACTAGTATGACA	abT	-0,07355189	-0,020712597	-0,057473391	0,182946713	-0,057473391
925_AAACCCAGTGTTCCAA	abT	0,064740901	-0,133239396	-0,168216719	0,671532649	0,321008706
925_CCTAAGATCGAGATGG	abT	0,219983564	-0,189989828	-0,244495587	0,851737696	0,061087145
801_AAACGCTTCGGCCCAA	abT	0,05220043	0,457643751	0,032921349	0,657613912	0,171392017
996_GTGATGTAGGTGATAT	abT	0,040402139	-0,279284725	0,468529327	-0,245529831	-0,128883991
1224_CTAACTTGTCACTGAT	abT	-0,199477838	-0,159238742	1,409375419	0,171394336	-0,024981004
1416_GTGTGATCACACGCCA	abT	-0,327690389	-0,222848683	-0,322270845	0,358399673	0,653236017
925_GGCTTGGGTTTCACTT	abT	0,267928013	-0,073687449	-0,106387608	0,386738006	-0,098816286
1130_ATTCTACCACCAGCTG	abT	0,402826584	0,568600486	-0,232252274	0,935616086	-0,204960672
925_GTCATCCCAGCACCCA	abT	-0,00460847	-0,206175377	0,22145806	0,545285746	0,350912126
943_CCCTTAGGTTCTGACA	abT	-0,235994376	-0,143260874	0,122955603	0,147270537	-0,165664091
966_AACAAAGAGCTTTCTT	abT	0,183140667	-0,087560179	-0,161601133	0,810310769	0,16807599
1238_CCCTCAATCGGCATAT	abT	-0,422477933	-0,392141761	-0,001594113	-0,175730703	0,222685621
1238_CTGTATTGTCCGGACT	abT	-0,399754369	0,395285055	-0,353453621	0,392245401	0,195161154
1195_CCTCACAAGGATATGT	gdT	-0,100696731	-0,19890153	0,189342914	0,812325977	-0,08186083
1238_GGGCTACAGTAGTGCG	gdT	-0,289659514	-0,162087502	-0,240636916	0,903715723	0,897917554
1397_TGCGATACAGAACGCA	gdT	0,193006795	0,128662079	-0,36646232	0,464853109	-0,060039997
1416_CGACAGCAGATGGGCT	gdT	-0,358659544	0,861341852	-0,493025058	0,517727474	0,533904417
1416_GTCTTTAGTTTCAGAC	gdT	-0,035447665	-0,161566439	-0,229408294	0,297387793	0,50502288
943_GTTATGGCAGCCTATA	gdT	0,179858477	-0,208577282	-0,187683616	0,629620594	-0,155204106
1125_GGTAGAGGTTTCCCAC	gdT	0,214206544	-0,115582916	-0,174195097	1,002333661	0,636594512
1066_GGTAGAGCACGACTAT	gdT	0,09696155	-0,22737036	-0,241297318	0,801448039	-0,02512646
1167_AAGTTCGGTCCTTTGC	gdT	-0,232768802	0,721187503	-0,215813364	0,355100533	0,123663065
1195_TAGAGTCAGCGGTAAC	gdT	0,633321265	-0,192456759	-0,249438014	0,86743684	0,812244007
1224_AGTCATGCAATTGTGC	gdT	-0,119798578	0,482586345	-0,251693549	0,864626338	0,233015833
1224_CATACAGGTGGACCAA	gdT	0,263037947	-0,224814139	0,090482578	0,907111219	-0,175446539
1238_AATCACGTCCATAGGT	gdT	0,533344219	-0,18763242	-0,266762606	0,60288517	0,724827886
1238_GACGCTGTCCGCTTAC	gdT	-0,258787834	0,676268675	-0,24024041	0,907209309	-0,208298941
1355_TGGCCAGTCTTCGGTC	gdT	0,189150419	-0,041706524	-0,056632447	1,098020619	0,610671935
1130_CCTCCAATCCTACCGT	gdT	0,380319025	0,382248198	0,381465704	0,935985738	-0,042372747
1416_AATGCCAGTACAGCGA	gdT	-0,189729729	0,321518155	-0,359838286	0,3646926	0,010276802
1224_GTCTCACTCACACCCT	gdT	-0,359447169	-0,368615832	-0,209643387	0,214447583	-0,277844291
1224_GTGAGCCGTAGTAAGT	gdT	0,242216074	-0,187563117	-0,224997131	0,312104379	-0,227003284
1238_ACGATGTTCACGAACT	gdT	-0,320423357	-0,252402859	-0,031041967	0,551018023	0,477171384
1238_TGTTACTGTTCCTAAG	gdT	-0,052222765	-0,221586376	-0,225737302	0,621194583	-0,184618184
1238_TGTTACTTCTGAACGT	gdT	0,6793636	0,503985544	-0,256818713	0,609854471	0,051838431
925_AGGCCACGTACTGACT	gdT	-0,03945058	-0,287008068	-0,080801397	0,633099918	0,594019729
925_TAGGTACAGACATATG	gdT	-0,080169142	0,428242811	-0,193280096	0,387621761	0,333171344
925_TGCTTCGAGATCGCCC	gdT	0,013233551	-0,212908389	-0,330906159	0,991844852	0,089492261
945_GAGCTGCGTTATGGTC	gdT	0,212894758	-0,106865655	-0,161665327	0,612824121	-0,128779445
1167_ATTTACCCAAATGATG	gdT	-0,047700719	-0,152096604	-0,248174218	0,525968378	0,035783652
1235_ATGAAAGCAGAGGTAC	gdT	-0,027403302	-0,083119734	-0,155458409	0,564939293	-0,163410691
1238_AGAGCCCTCACAATGC	gdT	-0,293796504	-0,263924743	-0,230165922	0,597915843	0,056247255
966-2_CCGTGAGAGAGGGTAA	gdT	-0,168356819	-0,123160188	1,343570712	0,266851926	-0,164245194
1235_TCAAGCAGTAGACTGG	gdT	-0,221531974	-0,255563252	0,743381022	0,264059275	0,067926699
1125_CTAAGTGTCTACGCAA	gdT	0,115687393	-0,12680039	0,218855344	0,632921213	-0,233706664
1224_ACGTCCTTCATTATCC	gdT	-0,115763664	0,181737699	-0,208893326	0,518824437	-0,259838894
925_GTCCTCATCCATGATG	gdT	-0,242720295	-0,191310207	-0,246119535	0,372667914	0,215438468
934_GTGACGCCAATGAACA	gdT	0,274841428	0,403809988	0,05839005	0,906107999	0,227412644
966_TCAGTTTTCGCCAGAC	gdT	-0,580757728	1,045533888	0,153152121	-0,192723871	-0,438137739
1224_AGCATCAGTGGCTTAT	gdT	0,734452119	-0,248866605	-0,29379883	0,508953955	-0,225751127
1224_CATGGTAAGCTGAAAT	gdT	0,194859912	-0,244412016	-0,305787956	0,386110004	-0,271774275
1235_TCAGTGATCCTTATAC	gdT	-0,368957054	-0,205557822	0,61773694	0,401646605	-0,014720555
1238_CGGGCATAGCACCGTC	gdT	-0,009246411	0,639778568	0,175812455	0,658573415	-0,162830867
1066_GCCGTGACAATCGTCA	gdT	-0,311108257	-0,229775201	0,515647497	0,389880679	0,081186914
1195_ACATTTCAGGGAGGAC	gdT	-0,01782335	-0,17720558	-0,231556043	0,84417351	0,017197049
1235_TATACCTTCTCACCCA	gdT	-0,387965359	-0,194549868	0,890187699	0,529561629	0,017419154
1238_AGTTAGCTCTTAGCTT	gdT	-0,02250084	0,649080819	-0,220007804	0,803891212	0,337982127
801_GCTGGGTCAAAGACTA	gdT	-0,432255887	-0,404092752	-0,056270479	-0,228623622	-0,394165641
945_GAAACCTTCACACCCT	gdT	-0,167163151	0,303487175	-0,410312269	0,470886858	0,238227829
996_CTCATGCGTGGACAGT	gdT	-0,172931637	-0,289343515	-0,347628644	0,633701643	0,323601884
898_GCAACATCAAACCACT	gdT	-0,196388199	-0,172196122	-0,201915488	0,690989003	0,120126922
996_TATTCCAAGGACGCTA	gdT	-0,274473897	0,607195706	-0,081942352	0,376119211	-0,145917697
**average abT**		**-0,07929738**	**-0,086101106**	**-0,027771141**	**0,422973835**	**0,082723578**
**average gdT**		**-0,027456151**	**0,033000903**	**-0,064245409**	**0,575303815**	**0,088393642**
