# Supplementary material for: EphA2 and phosphoantigen-mediated selective killing of medulloblastoma by γδT cells preserves neuronal and stem cell integrity
Source: Oncoimmunology. 2025 Apr 7;14(1):2485535. doi: 10.1080/2162402X.2025.2485535 (PMC11980450; doi:10.1080/2162402X.2025.2485535)
Supplement: Boutin et al_Table_S2.pdf [file KONI_A_2485535_SM7965.pdf]

Table S2

Boutin et al.

Ephrin-A2 and Phosphoantigen-Mediated Selective Killing of Medulloblastoma by γδT Cells Preserves Neuronal and Stem Cell Integrity

CIBERSORTx_GSE44971

Sample	Bcells	TCD4	TCD8	Tgd	NK	MoMaDC	granulocytes	P-value	Correlation	RMSE	HG-U133_Plus_2	age group
GSM1094863	0	0	0	0,296381	0	0,652177	0,05144213	0	0,709788221	0,703862	GSE44971-CBM-07	fetal
GSM1094864	0	0,042458	0	0,311428	0	0,646114	0	0	0,552661778	0,843202	GSE44971-CBM-08	fetal
GSM1094865	0	0,02244	0,007153	0,353972	0	0,616435	0	0	0,50876315	0,876936	GSE44971-CBM-09	fetal
GSM1094866	0	0,079161	0	0,273212	0	0,647627	0	0	0,53941579	0,851896	GSE44971-CBM-10	fetal
GSM1094867	0	0,043656	0	0,432214	0	0,524131	0	0	0,413776482	0,940446	GSE44971-CBM-11	fetal
GSM1094868	0	0,036438	0	0,341741	0	0,592212	0,02960967	0	0,440667092	0,924395	GSE44971-CBM-12	adult
GSM1094869	0	0,08187	0	0,381771	0	0,536359	0	0	0,461304163	0,9031	GSE44971-CBM-13	adult
GSM1094870	0	0,010311	0,051036	0,289743	0	0,548367	0,10054307	0	0,50784149	0,871125	GSE44971-CBM-14	adult
GSM1094871	0,012108	0,080714	0	0,259827	0,002582	0,644769	0	0	0,520897658	0,865392	GSE44971-CBM-15	adult
**average**	**0,001345**	**0,044116**	**0,006465**	**0,326699**	**0,000287**	**0,60091**	**0,02017721**					

SES_CIBERSORTx_GSE44971

Sample	Bcells	TCD4	TCD8	Tgd	NK	MoMaDC	granulocytes	P-value	Correlation	RMSE
GSM1094863	0	0	0	1,812349	0	3,988011	0,31456475	0	0,709788221	0,703862
GSM1094864	0	0,1723	0	1,263826	0	2,622038	0	0	0,552661778	0,843202
GSM1094865	0	0,050178	0,015994	0,791509	0	1,378398	0	0	0,50876315	0,876936
GSM1094866	0	0,314148	0	1,084229	0	2,570074	0	0	0,53941579	0,851896
GSM1094867	0	0,109619	0	1,085285	0	1,316087	0	0	0,413776482	0,940446
**fetal**	**0**	**0,129249**	**0,003199**	**1,20744**	**0**	**2,374922**	**0,06291295**			
GSM1094868	0	0,207553	0	1,94659	0	3,373301	0,16865976	0	0,440667092	0,924395
GSM1094869	0	0,511568	0	2,385505	0	3,351458	0	0	0,461304163	0,9031
GSM1094870	0	0,084573	0,418622	2,376607	0	4,497952	0,82469985	0	0,50784149	0,871125
GSM1094871	0,070364	0,469053	0	1,509937	0,015002	3,746958	0	0	0,520897658	0,865392
**adult**	**0,017591**	**0,318187**	**0,104655**	**2,05466**	**0,00375**	**3,742417**	**0,2483399**			

CIBERSORTx_GSE37418

Sample	Bcells	TCD4	TCD8	Tgd	NK	MoMaDC	granulocytes	P-value	Correlation	RMSE	Group
GSM918586	0,017527	0,101786	0	0,276659	0,009757	0,594271	0	0	0,745226239	0,674223	G3
GSM918588	0	0,1209	0	0,446285	0	0,377789	0,05502652	0	0,644504791	0,764481	G3
GSM918589	0	0,154907	0	0,370216	0	0,474877	0	0	0,543345164	0,839754	G3
GSM918594	0	0,152356	0	0,262159	0	0,585485	0	0	0,738920529	0,681943	G3
GSM918600	0,003214	0,09692	0	0,362661	0,012582	0,524623	0	0	0,726498127	0,693487	G3
GSM918601	0	0,063409	0,050397	0,368759	0	0,517435	0	0	0,534048051	0,84921	G3
GSM918611	0	0,191128	0	0,411861	0	0,39701	0	0	0,697986611	0,723199	G3
GSM918612	0	0,052031	0,020939	0,373635	0	0,553395	0	0	0,749222808	0,669547	G3
GSM918614	0	0,079907	0	0,381801	0	0,538292	0	0	0,592644438	0,805591	G3
GSM918624	0	0,038389	0,004163	0,31672	0	0,640728	0	0	0,717660113	0,696393	G3
GSM918629	0	0,12376	0	0,386114	0,001182	0,488943	0	0	0,625663426	0,778895	G3
GSM918636	0,000899	0,140854	0	0,376218	0	0,482029	0	0	0,582007313	0,812044	G3
GSM918637	0	0,13248	0,061592	0,318227	0	0,400966	0,08673552	0	0,653961179	0,760945	G3
GSM918639	0	0,126074	0	0,33397	0	0,510225	0,02973084	0	0,593287676	0,803682	G3
GSM918640	0	0,101895	0,102006	0,290704	0	0,505395	0	0	0,527351528	0,850685	G3
GSM918578	0	0,049312	0	0,421318	0	0,529371	0	0	0,6527244	0,756816	G4
GSM918579	0	0,095753	0	0,428402	0	0,465948	0,00989776	0	0,521313151	0,858146	G4
GSM918581	0	0,129461	0	0,339247	0	0,531292	0	0	0,53895604	0,844336	G4
GSM918583	0	0,18862	0	0,378516	0	0,432863	0	0	0,499878255	0,868487	G4
GSM918584	0	0,15149	0	0,37631	0	0,4722	0	0	0,530507863	0,848942	G4
GSM918585	0	0,134164	0	0,397818	0	0,468018	0	0	0,615534399	0,786797	G4
GSM918590	0	0,017283	0,1045	0,282472	0	0,595745	0	0	0,712224729	0,703973	G4
GSM918591	0	0,093873	0	0,452079	0	0,454048	0	0	0,471107816	0,89494	G4
GSM918592	0	0,028188	0	0,380902	0	0,555987	0,03492239	0	0,636919967	0,770444	G4
GSM918595	0	0,082028	0	0,244743	0	0,673229	0	0	0,775950041	0,635449	G4
GSM918598	0	0,138214	0	0,403366	0	0,45842	0	0	0,558704282	0,829278	G4
GSM918599	0	0,139318	0	0,379309	0	0,481373	0	0	0,618088138	0,784836	G4
GSM918602	0	0,146907	0	0,271704	0	0,581389	0	0	0,753500263	0,668529	G4
GSM918605	0	0,114419	0,003569	0,352445	0	0,529568	0	0	0,66108825	0,750159	G4
GSM918610	0	0,116674	0	0,381158	0	0,502168	0	0	0,4638473	0,897534	G4
GSM918613	0	0,056877	0	0,392485	0	0,533965	0,01667371	0	0,612409808	0,790184	G4
GSM918615	0,000167	0,088609	0	0,312618	0	0,57636	0,02224662	0	0,734676686	0,683719	G4
GSM918616	0	0,165408	0	0,369675	0	0,453177	0,01173978	0	0,548080267	0,835866	G4
GSM918622	0,0067	0,09779	0	0,423474	0	0,442807	0,02922917	0	0,566625916	0,823888	G4
GSM918623	0	0,147114	0	0,341039	0	0,511847	0	0	0,498484887	0,871822	G4
GSM918626	0	0,102766	0,08425	0,121454	0,007254	0,684276	0	0	0,825977119	0,580267	G4
GSM918627	0	0,161139	0	0,409963	0	0,398386	0,03051227	0	0,578375764	0,814343	G4
GSM918630	0	0,167374	0	0,409457	0	0,423169	0	0	0,471492241	0,888864	G4
GSM918631	0,000629	0,116191	0,043035	0,280929	0,003502	0,543386	0,01232915	0	0,540565236	0,842619	G4
GSM918632	0	0,029831	0,061692	0,413638	0	0,49484	0	0	0,555617118	0,834545	G4
GSM918633	0	0,076168	0	0,36045	0	0,563382	0	0	0,671703384	0,740275	G4
GSM918634	0	0,056741	0	0,377199	0	0,56606	0	0	0,589257565	0,8095	G4
GSM918635	0	0,094336	0,049433	0,28987	0	0,566362	0	0	0,441561448	0,914181	G4
GSM918645	0	0,194384	0	0,376588	0	0,414603	0,01442548	0	0,509443562	0,86147	G4
GSM918646	0	0,017692	0,070861	0,32024	0	0,591207	0	0	0,633229101	0,773426	G4
GSM918647	0	0,204733	0,003419	0,304103	0	0,487744	0	0	0,534670496	0,844582	G4
GSM918648	0	0,0979	0	0,377345	0	0,524755	0	0	0,569415941	0,822842	G4
GSM918650	0	0,165699	0	0,431637	0	0,345813	0,05685123	0	0,425347739	0,917313	G4
GSM918652	0	0,165665	0	0,339137	0	0,495198	0	0	0,542519369	0,840117	G4
GSM918653	0	0,070158	0	0,459559	0	0,470283	0	0	0,440506923	0,918691	G4
GSM918582	0	0,130967	0	0,344869	0	0,524164	0	0	0,727414629	0,694358	SHH
GSM918606	0	0,129215	0	0,269355	0	0,60143	0	0	0,658581927	0,751392	SHH
GSM918607	0	0,068069	0,045646	0,216363	0	0,669922	0	0	0,858599791	0,540692	SHH
GSM918608	0	0,05357	0	0,278668	0	0,667762	0	0	0,749673853	0,66318	SHH
GSM918609	0	0,095336	0	0,287636	0	0,617029	0	0	0,722486335	0,693345	SHH
GSM918619	0	0,088588	0	0,308486	0	0,602926	0	0	0,778059103	0,63952	SHH
GSM918620	0	0,049913	0,022288	0,313055	0	0,614745	0	0	0,671419956	0,740145	SHH
GSM918621	0	0,065519	0	0,27318	0	0,661301	0	0	0,851038968	0,548968	SHH
GSM918649	0	0	0,088517	0,316578	0	0,594906	0	0	0,816036975	0,598646	SHH
GSM918651	0	0,167787	0,024434	0,293743	0	0,514037	0	0	0,677514809	0,739274	SHH
GSM918587	0,000384	0,094471	0,046486	0,200965	0,007429	0,650266	0	0	0,861080381	0,54373	Unknown
GSM918596	0	0,069876	0	0,32802	0	0,602105	0	0	0,684991501	0,72799	Unknown
GSM918597	0	0,104133	0	0,354963	0	0,540904	0	0	0,550154392	0,837322	Unknown
GSM918604	0	0,065128	0	0,335515	0	0,599357	0	0	0,809303792	0,605312	Unknown
GSM918617	0	0,119203	0,008339	0,347081	0	0,525376	0	0	0,585912495	0,809549	Unknown
GSM918618	0,009756	0,075906	0,011383	0,225095	0	0,67786	0	0	0,81797973	0,588089	Unknown
GSM918628	0	0,016019	0,07443	0,158443	0	0,751108	0	0	0,842289815	0,546556	Unknown
GSM918644	0,001839	0,228605	0	0,249077	0	0,520479	0	0	0,631841447	0,775605	Unknown
GSM918580	0	0,013484	0,083301	0,232499	0	0,670716	0	0	0,78290609	0,628202	WNT
GSM918593	0,000598	0,144525	0	0,326881	0	0,527996	0	0	0,618760574	0,784224	WNT
GSM918603	0	0,110514	0,005825	0,293997	0	0,589664	0	0	0,639740205	0,767274	WNT
GSM918625	0	0,079669	0,035443	0,275276	0	0,609613	0	0	0,70900619	0,706534	WNT
GSM918638	0	0,20525	0,005706	0,260029	0	0,529014	0	0	0,568705493	0,821243	WNT
GSM918641	0	0,089878	0,013671	0,252497	0	0,643954	0	0	0,755718511	0,659483	WNT
GSM918642	0	0,178385	0,055696	0,259846	0	0,506074	0	0	0,538607926	0,841881	WNT
GSM918643	0	0,066477	0,042738	0,324364	0	0,566421	0	0	0,612692094	0,789519	WNT
average	0,000549	0,106442	0,016102	0,33162	0,000549	0,53934	0,00539895				

SES_CIBERSORTx_GSE37418

Sample	Bcells	TCD4	TCD8	Tgd	NK	MoMaDC	granulocytes	P-value	Correlation	RMSE
GSM918578	0	0,183557	0	1,568301	0	1,970515	0	0	0,6527244	0,756816
GSM918579	0	0,27968	0	1,251297	0	1,360963	0,02890987	0	0,521313151	0,858146
GSM918580	0	0,10025	0,619303	1,72852	0	4,986452	0	0	0,78290609	0,628202
GSM918581	0	0,686968	0	1,800173	0	2,819239	0	0	0,53895604	0,844336
GSM918582	0	0,824627	0	2,171454	0	3,30038	0	0	0,727414629	0,694358
GSM918583	0	0,813135	0	1,631768	0	1,866055	0	0	0,499878255	0,868487
GSM918584	0	0,569863	0	1,415568	0	1,77628	0	0	0,530507863	0,848942
GSM918585	0	0,371289	0	1,100937	0	1,29521	0	0	0,615534399	0,786797
GSM918586	0,156738	0,910249	0	2,474101	0,087255	5,314441	0	0	0,745226239	0,674223
GSM918587	0,002464	0,60601	0,298194	1,289141	0,047652	4,171295	0	0	0,861080381	0,54373
GSM918588	0	0,239463	0	0,883945	0	0,748278	0,10898972	0	0,644504791	0,764481
GSM918589	0	0,281817	0	0,673522	0	0,863927	0	0	0,543345164	0,839754
GSM918590	0	0,128648	0,777846	2,102583	0	4,434433	0	0	0,712224729	0,703973
GSM918591	0	0,269026	0	1,295588	0	1,301229	0	0	0,471107816	0,89494
GSM918592	0	0,116059	0	1,56827	0	2,289141	0,14378433	0	0,636919967	0,770444
GSM918593	0,001596	0,385754	0	0,872483	0	1,409282	0	0	0,618760574	0,784224
GSM918594	0	0,695899	0	1,197432	0	2,674245	0	0	0,738920529	0,681943
GSM918595	0	0,868003	0	2,589817	0	7,123974	0	0	0,775950041	0,635449
GSM918596	0	0,364433	0	1,71077	0	3,140244	0	0	0,684991501	0,72799
GSM918597	0	0,304978	0	1,039593	0	1,584164	0	0	0,550154392	0,837322
GSM918598	0	0,511018	0	1,491362	0	1,694913	0	0	0,558704282	0,829278
GSM918599	0	0,479896	0	1,306567	0	1,658137	0	0	0,618088138	0,784836
GSM918600	0,006782	0,204529	0	0,765323	0,026552	1,107111	0	0	0,726498127	0,693487
GSM918601	0	0,225597	0,179304	1,311977	0	1,840941	0	0	0,534048051	0,84921
GSM918602	0	0,682375	0	1,262056	0	2,700531	0	0	0,753500263	0,668529
GSM918603	0	0,480095	0,025304	1,277187	0	2,561628	0	0	0,639740205	0,767274
GSM918604	0	0,286149	0	1,474122	0	2,633344	0	0	0,809303792	0,605312
GSM918605	0	0,494871	0,015436	1,524354	0	2,290426	0	0	0,66108825	0,750159
GSM918606	0	0,611231	0	1,274136	0	2,844958	0	0	0,658581927	0,751392
GSM918607	0	0,642491	0,430844	2,042225	0	6,323302	0	0	0,858599791	0,540692
GSM918608	0	0,273352	0	1,421968	0	3,407409	0	0	0,749673853	0,66318
GSM918609	0	0,197351	0	0,595423	0	1,277287	0	0	0,722486335	0,693345
GSM918610	0	0,380962	0	1,244554	0	1,639675	0	0	0,4638473	0,897534
GSM918611	0	0,345735	0	0,745022	0	0,718157	0	0	0,697986611	0,723199
GSM918612	0	0,131526	0,052931	0,944479	0	1,398878	0	0	0,749222808	0,669547
GSM918613	0	0,314868	0	2,172788	0	2,956021	0,09230537	0	0,612409808	0,790184
GSM918614	0	0,047719	0	0,228003	0	0,321457	0	0	0,592644438	0,805591
GSM918615	0,001502	0,799077	0	2,819199	0	5,197621	0,20062044	0	0,734676686	0,683719
GSM918616	0	0,555363	0	1,241193	0	1,521553	0,0394166	0	0,548080267	0,835866
GSM918617	0	0,386847	0,027062	1,126375	0	1,704991	0	0	0,585912495	0,809549
GSM918618	0,099661	0,775415	0,116281	2,299437	0	6,92462	0	0	0,81797973	0,588089
GSM918619	0	0,420434	0	1,464056	0	2,861454	0	0	0,778059103	0,63952
GSM918620	0	0,376324	0,168042	2,360325	0	4,634967	0	0	0,671419956	0,740145
GSM918621	0	0,351575	0	1,465883	0	3,548536	0	0	0,851038968	0,548968
GSM918622	0,0198	0,288988	0	1,251452	0	1,308584	0,08637812	0	0,566625916	0,823888
GSM918623	0	0,539308	0	1,25022	0	1,876388	0	0	0,498484887	0,871822
GSM918624	0	0,21269	0,023066	1,754768	0	3,549915	0	0	0,717660113	0,696393
GSM918625	0	0,280741	0,124895	0,970032	0	2,148188	0	0	0,70900619	0,706534
GSM918626	0	2,22914	1,827485	2,634491	0,157346	14,84286	0	0	0,825977119	0,580267
GSM918627	0	0,760985	0	1,936066	0	1,881396	0,1440955	0	0,578375764	0,814343
GSM918628	0	0,304358	1,41417	3,010416	0	14,27105	0	0	0,842289815	0,546556
GSM918629	0	0,483824	0	1,509462	0,004622	1,911458	0	0	0,625663426	0,778895
GSM918630	0	0,582936	0	1,426071	0	1,473827	0	0	0,471492241	0,888864
GSM918631	0,002877	0,53179	0,196964	1,285777	0,016029	2,487012	0,05642902	0	0,540565236	0,842619
GSM918632	0	0,068854	0,142392	0,954731	0	1,142156	0	0	0,555617118	0,834545
GSM918633	0	0,286017	0	1,353521	0	2,11555	0	0	0,671703384	0,740275
GSM918634	0	0,396633	0	2,63669	0	3,956866	0	0	0,589257565	0,8095
GSM918635	0	0,373204	0,195563	1,146761	0	2,240598	0	0	0,441561448	0,914181
GSM918636	0,002297	0,359813	0	0,961056	0	1,231351	0	0	0,582007313	0,812044
GSM918637	0	0,399584	0,185773	0,959834	0	1,20939	0,26161096	0	0,653961179	0,760945
GSM918638	0	0,688991	0,019154	0,872875	0	1,775811	0	0	0,568705493	0,821243
GSM918639	0	0,420987	0	1,115197	0	1,703751	0,09927757	0	0,593287676	0,803682
GSM918640	0	0,334342	0,334707	0,953868	0	1,65832	0	0	0,527351528	0,850685
GSM918641	0	0,669193	0,101791	1,879984	0	4,79461	0	0	0,755718511	0,659483
GSM918642	0	0,42333	0,132173	0,616648	0	1,20098	0	0	0,538607926	0,841881
GSM918643	0	0,157626	0,101338	0,769115	0	1,343069	0	0	0,612692094	0,789519
GSM918644	0,005095	0,633269	0	0,689979	0	1,441805	0	0	0,631841447	0,775605
GSM918645	0	0,78315	0	1,517226	0	1,670384	0,0581185	0	0,509443562	0,86147
GSM918646	0	0,069408	0,277996	1,256349	0	2,319391	0	0	0,633229101	0,773426
GSM918647	0	0,528922	0,008834	0,78564	0	1,26007	0	0	0,534670496	0,844582
GSM918648	0	0,32173	0	1,240066	0	1,724501	0	0	0,569415941	0,822842
GSM918649	0	0	0,601399	2,150892	0	4,041908	0	0	0,816036975	0,598646
GSM918650	0	0,383092	0	0,997935	0	0,799513	0,13143888	0	0,425347739	0,917313
GSM918651	0	0,733782	0,106856	1,284625	0	2,248036	0	0	0,677514809	0,739274
GSM918652	0	0,719068	0	1,472025	0	2,149407	0	0	0,542519369	0,840117
GSM918653	0	0,313887	0	2,056084	0	2,104063	0	0	0,440506923	0,918691
**average**	**0,003932**	**0,450713**	**0,111909**	**1,433199**	**0,004467**	**2,737893**	**0,01909704**			
