# Supplementary material for: EphA2 and phosphoantigen-mediated selective killing of medulloblastoma by γδT cells preserves neuronal and stem cell integrity
Source: Oncoimmunology. 2025 Apr 7;14(1):2485535. doi: 10.1080/2162402X.2025.2485535 (PMC11980450; doi:10.1080/2162402X.2025.2485535)
Supplement: Boutin et al_Table_S1.pdf [file KONI_A_2485535_SM7964.pdf]

Table S1

Boutin et al.

Ephrin-A2 and Phosphoantigen-Mediated Selective Killing of Medulloblastoma by γδT Cells Preserves Neuronal and Stem Cell Integrity

Gene Signatures

Naive	Effector Memory	Resident	Pre-exhausted	Exhausted
sell	ccl20	itga1	xcl1	pdcd1
tcf7	il7r	itgae	tox	havcr2
ccr7	cd69	cxcr6		prf1
lef1	gzma	ccr2		tigit
	gzmk	cxcr3		lag3
	ccl5	ccr5		gzmb
	klrc1	lfa1		entpd1
	ifng			ifng
	mki67			
	gzmb			
	tnfsf9			
	ccl3			
