# Supplementary material for: EphA2 and phosphoantigen-mediated selective killing of medulloblastoma by γδT cells preserves neuronal and stem cell integrity
Source: Oncoimmunology. 2025 Apr 7;14(1):2485535. doi: 10.1080/2162402X.2025.2485535 (PMC11980450; doi:10.1080/2162402X.2025.2485535)
Supplement: Boutin et al_Table_S3.pdf [file KONI_A_2485535_SM7963.pdf]

Table S3

Boutin et al.

Ephrin-A2 and Phosphoantigen-Mediated Selective Killing of Medulloblastoma by γδT Cells Preserves Neuronal and Stem Cell Integrity

CIBERSORTx_GSE85217_WNT

Sample	Bcells	TCD4	TCD8	Tgd	NK	MoMaDC	granulocytes	P-value	Correlation	RMSE	ratio mono/T cells
GSM2261548	0	0,202847	0,009953	0,051976	0	0,735224	0	0	0,601161	0,799082	2,776778664
GSM2261551	0	0,230386	0	0,063471	0	0,642135	0,06400721	0	0,495996	0,870733	2,185192822
GSM2261553	0	0,1026	0	0,082732	0	0,731776	0,082891911	0	0,752767	0,667515	3,948446011
GSM2261580	0	0,181016	0	0,083852	0	0,735132	0	0	0,646753	0,763604	2,775469891
GSM2261589	0	0,222129	0	0,14597	0	0,624449	0,007452584	0	0,427527	0,909766	1,696419392
GSM2261591	0	0,15856	0,048542	0,07621	0	0,716689	0	0	0,603858	0,796594	2,529690511
GSM2261609	0,003235	0,281271	0	0,071934	0	0,630222	0,013338154	0	0,494501	0,870499	1,78429972
GSM2261648	0	0,297956	0	0,126718	0	0,505481	0,069844985	0	0,428749	0,905268	1,190278652
GSM2261687	0,00784	0,192423	0	0,066743	0	0,732994	0	0	0,650231	0,761303	2,828283218
GSM2261702	0	0,270884	0	0,082514	0	0,646602	0	0	0,484585	0,876787	1,829671303
GSM2261706	0	0,122613	0,039936	0,028074	0,004353	0,805023	0	0	0,650917	0,758661	4,223106776
GSM2261707	0	0,193827	0,062548	0,031762	0	0,711863	0	0	0,656084	0,758007	2,470576124
GSM2261712	0	0,268773	0	0,131454	0	0,531952	0,067820943	0	0,443152	0,897929	1,329126393
GSM2261713	0	0,289174	0	0,155326	0	0,455228	0,100272243	0,01	0,327465	0,956319	1,02413381
GSM2261715	0	0,182856	0,007463	0,128899	0,008961	0,619611	0,052209699	0	0,621526	0,786082	1,941030264
GSM2261717	0	0,266114	0,033853	0,117162	0	0,573514	0,009357517	0	0,444368	0,89774	1,374907953
GSM2261726	0	0,280101	0	0,074539	0	0,643271	0,002088981	0	0,467365	0,887389	1,813870584
GSM2261741	0	0,292879	0	0,075263	0	0,631858	0	0	0,499357	0,867485	1,716343972
GSM2261752	0	0,21355	0,04358	0,079559	0	0,432461	0,230851087	0	0,619838	0,792198	1,28445366
GSM2261766	0	0,246785	0	0,115608	0	0,576879	0,060727775	0	0,393972	0,927413	1,591860252
GSM2261771	0	0,276457	0	0,077898	0	0,593149	0,052495762	0	0,472319	0,88325	1,673880253
GSM2261822	0	0,231146	0,011228	0,059906	0,010203	0,563474	0,124042206	0	0,477189	0,880458	1,864080211
GSM2261837	0	0,199828	0,114011	0,077993	0	0,541201	0,066967071	0	0,490694	0,870835	1,381204768
GSM2261849	0	0,186156	0,019278	0,111531	0	0,661065	0,021970035	0	0,477462	0,881748	2,085606501
GSM2261862	0	0,285984	0	0,111258	0	0,558324	0,044434061	0	0,38411	0,931173	1,405500176
GSM2261890	0	0,24424	0	0,113041	0	0,596022	0,046696534	0	0,532397	0,846199	1,66821324
GSM2261905	0	0,258541	0,008895	0,096104	0,001757	0,489725	0,144977412	0	0,623212	0,791486	1,347096345
GSM2261930	0	0,158246	0,010114	0,077596	0	0,698481	0,055563593	0	0,636453	0,77174	2,839866138
GSM2261933	0	0,359245	0	0,107839	0,007014	0,443297	0,0826042	0	0,382084	0,928264	0,94907318
GSM2261958	0,007505	0,285152	0	0,110347	0	0,526307	0,070689172	0	0,521774	0,853607	1,330742907
GSM2261971	0	0,243077	0,020688	0,083016	0	0,488494	0,164724697	0	0,380234	0,933487	1,408651294
GSM2261973	0	0,271411	0	0,119691	0	0,590298	0,018600506	0	0,515824	0,856524	1,509319417
GSM2261975	0	0,184448	0,043836	0,039236	0	0,641548	0,090931873	0	0,55822	0,829593	2,398127776
GSM2261986	0,00124	0,249606	0	0,063378	0	0,662343	0,023433706	0	0,631936	0,778715	2,11622088
GSM2261990	0	0,226879	0,036402	0,09891	0	0,579761	0,05804753	0	0,535505	0,844257	1,600706202
GSM2262002	0	0,369887	0	0,153635	0	0,410726	0,065752073	0	0,33575	0,950311	0,784542846
GSM2262024	0	0,272155	0	0,03233	0	0,695515	0	0	0,617616	0,788453	2,284234897
GSM2262040	0	0,309325	0	0,126635	0	0,552313	0,011726849	0	0,418294	0,911784	1,266888876
GSM2262069	0	0,265867	0,010407	0,097151	0	0,581999	0,044575093	0	0,478341	0,878974	1,558541492
GSM2262071	0	0,369748	0	0,087348	0	0,491704	0,051199264	0	0,366708	0,937655	1,075710263
GSM2262088	0	0,25512	0	0,076265	0	0,642922	0,025692847	0	0,48021	0,879788	1,940107295
GSM2262095	0	0,184	0	0,108016	0	0,688758	0,019225697	0	0,608543	0,79352	2,358624745
GSM2262113	0,001595	0,277493	0,023408	0,065719	0	0,61509	0,016694575	0	0,537758	0,843509	1,677730455
GSM2262121	0	0,312992	0	0,088829	0	0,56968	0,028499006	0	0,375882	0,936192	1,417744909
GSM2262136	0	0,178014	0	0,09304	0	0,726567	0,00238027	0	0,579535	0,81457	2,680531852
GSM2262147	0	0,172751	0,029465	0,082473	0	0,715311	0	0	0,573235	0,818926	2,512602228
GSM2262154	0	0,21083	0,029644	0,078914	0	0,654238	0,026373333	0	0,46646	0,888574	2,048408961
GSM2262160	0,000522	0,265497	0,030302	0,075124	0	0,62837	0,000185299	0	0,630723	0,782532	1,694069608
GSM2262171	0	0,348738	0	0,048763	0,006992	0,433744	0,161763263	0	0,537696	0,846308	1,091179185
GSM2262172	0	0,337613	0	0,095994	0	0,566392	0	0	0,528923	0,849809	1,306231759
GSM2262197	0	0,234439	0	0,100045	0	0,665516	0	0	0,549377	0,835332	1,9896801
GSM2262210	0,002684	0,257471	0,003313	0,083007	0	0,611948	0,041577589	0	0,513449	0,85839	1,780000345
GSM2262212	0	0,286277	0	0,08499	0	0,604633	0,02410046	0	0,507229	0,862239	1,628565269
GSM2262214	0	0,221815	0,006724	0,080304	0,002879	0,680318	0,007961011	0	0,599285	0,801004	2,202803006
GSM2262220	0	0,190935	0,067602	0,043792	0	0,697671	0	0	0,593125	0,805085	2,30765542
GSM2262222	0	0,364278	0	0,107976	0	0,504768	0,022978307	0	0,38142	0,930139	1,068848281
GSM2262223	0,001351	0,276439	0	0,133443	0	0,588767	0	0	0,391807	0,927735	1,436434327
GSM2262225	0,003118	0,363386	0	0,117105	0	0,501064	0,015327174	0	0,501586	0,866181	1,042817544
GSM2262230	0,016777	0,075026	0,088128	0,026459	0,00829	0,785319	0	0	0,770196	0,650453	4,141688821
GSM2262232	0	0,194163	0,036081	0,137643	0	0,632113	0	0	0,586991	0,810163	1,718226336
GSM2262245	0	0,303648	0,011658	0,08275	0	0,57883	0,023113432	0	0,420081	0,912262	1,45413856
GSM2262250	0,001545	0,360328	0	0,13928	0	0,434577	0,064271135	0	0,376134	0,93091	0,869836116
GSM2262252	0	0,250175	0	0,060958	0	0,688867	0	0	0,585842	0,810857	2,214055884
GSM2262257	0	0,311845	0	0,139699	0	0,47936	0,06909674	0	0,339814	0,950503	1,061601758
GSM2262267	0	0,283765	0	0,117009	0	0,561461	0,03776464	0	0,457289	0,890564	1,400942426
GSM2262279	0	0,349345	0	0,150509	0	0,432048	0,068097266	0	0,381033	0,92843	0,864348683
GSM2262280	0	0,15559	0,080586	0	0,008489	0,755335	0	0	0,745495	0,67854	3,198188134
GSM2262281	0	0,295891	0	0,108852	0	0,595256	0	0	0,474648	0,881185	1,470699536
GSM2262292	0	0,273428	0	0,067554	0	0,655728	0,003289483	0	0,575192	0,81883	1,923053852
GSM2262304	0	0,307258	0,033101	0,089618	0	0,527071	0,042952588	0	0,380803	0,931274	1,225814456
average	0,000677	0,252038	0,013725	0,090268	0,000842	0,603855	0,038594841				

SES_CIBERSORTx_GSE85217_WNT

Sample	Bcells	TCD4	TCD8	Tgd	NK	MoMaDC	granulocytes	P-value	Correlatior	RMSE		Tab
GSM2261548	0	1,219359	0,059829	0,312437	0	4,41959	0	0	0,601161	0,799082		1,279188
GSM2261551	0	0,650311	0	0,179161	0	1,812556	0,180673252	0	0,495996	0,870733		0,650311
GSM2261553	0	0,970223	0	0,782345	0	6,919919	0,783854127	0	0,752767	0,667515		0,970223
GSM2261580	0	0,773	0	0,358076	0	3,139267	0	0	0,646753	0,763604		0,773
GSM2261589	0	0,600414	0	0,394556	0	1,687885	0,020144328	0	0,427527	0,909766		0,600414
GSM2261591	0	0,628135	0,192298	0,301905	0	2,839168	0	0	0,603858	0,796594		0,820433
GSM2261609	0,010798	0,938674	0	0,240061	0	2,103217	0,044512929	0	0,494501	0,870499		0,938674
GSM2261648	0	0,789512	0	0,335772	0	1,339401	0,185072278	0	0,428749	0,905268		0,789512
GSM2261687	0,051988	1,275957	0	0,442572	0	4,860486	0	0	0,650231	0,761303		1,275957
GSM2261702	0	0,923946	0	0,281444	0	2,205469	0	0	0,484585	0,876787		0,923946
GSM2261706	0	1,107842	0,360831	0,25366	0,039331	7,273597	0	0	0,650917	0,758661		1,468673
GSM2261707	0	0,62448	0,201519	0,102331	0	2,293509	0	0	0,656084	0,758007		0,825999
GSM2261712	0	0,780267	0	0,381621	0	1,544295	0,196889086	0	0,443152	0,897929		0,780267
GSM2261713	0	1,231312	0	0,661385	0	1,938375	0,426962692	0,01	0,327465	0,956319		1,231312
GSM2261715	0	2,384368	0,09731	1,680786	0,11685	8,07947	0,680792414	0	0,621526	0,786082		2,481679
GSM2261717	0	0,576193	0,073299	0,253681	0	1,241779	0,020261018	0	0,444368	0,89774		0,649492
GSM2261726	0	0,843475	0	0,224461	0	1,937098	0,006290599	0	0,467365	0,887389		0,843475
GSM2261741	0	0,966768	0	0,248436	0	2,085709	0	0	0,499357	0,867485		0,966768
GSM2261752	0	0,607497	0,123974	0,226325	0	1,230244	0,656714684	0	0,619838	0,792198		0,731471
GSM2261766	0	0,664672	0	0,311371	0	1,553724	0,163559788	0	0,393972	0,927413		0,664672
GSM2261771	0	1,177005	0	0,331648	0	2,525304	0,223498333	0	0,472319	0,88325		1,177005
GSM2261822	0	1,045304	0,050777	0,270909	0,046142	2,548178	0,560951152	0	0,477189	0,880458		1,096081
GSM2261837	0	0,418372	0,238701	0,163292	0	1,133092	0,140206462	0	0,490694	0,870835		0,657073
GSM2261849	0	0,314334	0,032551	0,188326	0	1,11624	0,037097479	0	0,477462	0,881748		0,346885
GSM2261862	0	0,499862	0	0,194464	0	0,975875	0,077664735	0	0,38411	0,931173		0,499862
GSM2261890	0	0,619944	0	0,286927	0	1,512854	0,118527598	0	0,532397	0,846199		0,619944
GSM2261905	0	1,110632	0,038213	0,412842	0,007548	2,103742	0,622788789	0	0,623212	0,791486		1,148844
GSM2261930	0	0,985164	0,062964	0,483073	0	4,348406	0,345912187	0	0,636453	0,77174		1,048128
GSM2261933	0	0,559936	0	0,168083	0,010933	0,690944	0,128750817	0	0,382084	0,928264		0,559936
GSM2261958	0,011552	0,438914	0	0,169848	0	0,810105	0,108806563	0	0,521774	0,853607		0,438914
GSM2261971	0	0,586076	0,049881	0,200159	0	1,177795	0,397163327	0	0,380234	0,933487		0,635957
GSM2261973	0	0,703212	0	0,310114	0	1,529432	0,048192981	0	0,515824	0,856524		0,703212
GSM2261975	0	0,888188	0,211086	0,188936	0	3,089292	0,437870808	0	0,55822	0,829593		1,099273
GSM2261986	0,005129	1,032522	0	0,26217	0	2,739855	0,09693615	0	0,631936	0,778715		1,032522
GSM2261990	0	0,56255	0,090259	0,245249	0	1,437528	0,143929784	0	0,535505	0,844257		0,652809
GSM2262002	0	0,893512	0	0,371127	0	0,992164	0,158833099	0	0,33575	0,950311		0,893512
GSM2262024	0	1,63826	0	0,194614	0	4,186717	0	0	0,617616	0,788453		1,63826
GSM2262040	0	0,932501	0	0,381758	0	1,66502	0,035352118	0	0,418293	0,911784		0,932501
GSM2262069	0	0,680836	0,026651	0,248785	0	1,490391	0,114148486	0	0,478341	0,878974		0,707488
GSM2262071	0	0,546359	0	0,12907	0	0,726566	0,075654574	0	0,366708	0,937655		0,546359
GSM2262088	0	0,492339	0	0,147179	0	1,240733	0,049582934	0	0,48021	0,879788		0,492339
GSM2262095	0	0,472592	0	0,277432	0	1,769026	0,049379866	0	0,608543	0,79352		0,472592
GSM2262113	0,005425	0,943827	0,079617	0,223527	0	2,09208	0,056782546	0	0,537758	0,843509		1,023443
GSM2262121	0	0,547072	0	0,155263	0	0,995732	0,04981286	0	0,375882	0,936192		0,547072
GSM2262136	0	1,307251	0	0,68324	0	5,335573	0,017479619	0	0,579535	0,81457		1,307251
GSM2262147	0	0,689361	0,117579	0,32911	0	2,854441	0	0	0,573235	0,818926		0,80694
GSM2262154	0	0,432261	0,060778	0,161797	0	1,341372	0,054072745	0	0,46646	0,888574		0,493039
GSM2262160	0,002435	1,239096	0,14142	0,350611	0	2,932648	0,000864805	0	0,630723	0,782532		1,380515
GSM2262171	0	0,828916	0	0,115904	0,01662	1,030968	0,384495868	0	0,537696	0,846308		0,828916
GSM2262172	0	0,893341	0	0,254006	0	1,4987	0	0	0,528923	0,849809		0,893341
GSM2262197	0	0,93589	0	0,399386	0	2,656772	0	0	0,549377	0,835332		0,93589
GSM2262210	0,008346	0,800676	0,010302	0,258134	0	1,903019	0,1292969	0	0,513449	0,85839		0,810978
GSM2262212	0	0,868382	0	0,257807	0	1,834073	0,073105568	0	0,507229	0,862239		0,868382
GSM2262214	0	0,831889	0,025216	0,30117	0,010798	2,551452	0,029856826	0	0,599285	0,801004		0,857105
GSM2262220	0	0,717963	0,254198	0,164668	0	2,623408	0	0	0,593125	0,805085		0,972161
GSM2262222	0	1,098842	0	0,325708	0	1,522628	0,069313887	0	0,38142	0,930139		1,098842
GSM2262223	0,002961	0,60565	0	0,29236	0	1,289931	0	0	0,391807	0,927735		0,60565
GSM2262225	0,008029	0,935722	0	0,301546	0	1,290244	0,039467598	0	0,501586	0,866181		0,935722
GSM2262230	0,187793	0,839785	0,986439	0,296164	0,09279	8,79027	0	0	0,770196	0,650453		1,826224
GSM2262232	0	0,52669	0,097873	0,373372	0	1,714677	0	0	0,586991	0,810163		0,624563
GSM2262245	0	0,71402	0,027414	0,194585	0	1,361102	0,054350575	0	0,420081	0,912262		0,741434
GSM2262250	0,003238	0,755281	0	0,291944	0	0,910915	0,134718479	0	0,376134	0,93091		0,755281
GSM2262252	0	0,915934	0	0,223179	0	2,522059	0	0	0,585842	0,810857		0,915934
GSM2262257	0	0,547718	0	0,245363	0	0,841936	0,121359928	0	0,339814	0,950503		0,547718
GSM2262267	0	0,726239	0	0,29946	0	1,436944	0,096650788	0	0,457289	0,890564		0,726239
GSM2262279	0	0,721178	0	0,310706	0	0,891908	0,140577919	0	0,381033	0,92843		0,721178
GSM2262280	0	1,981149	1,026112	0	0,108094	9,617787	0	0	0,745495	0,67854		3,007261
GSM2262281	0	0,857169	0	0,315335	0	1,724401	0	0	0,474648	0,881185		0,857169
GSM2262292	0	1,001759	0	0,247499	0	2,402391	0,012051686	0	0,575192	0,81883		1,001759
GSM2262304	0	0,622971	0,067112	0,181701	0	1,068645	0,087087085	0	0,380803	0,931274		0,690083
average	0,004253	0,843441	0,068631	0,30217	0,006416	2,390716	0,126976016					

CIBERSORTx_GSE85217_SHH

Sample	Bcells	TCD4	TCD8	Tgd	NK	MoMaDC	granulocytes	P-value	Correlation	RMSE	ratio mono/T cells
GSM2261538	0	0,203724	0,000668	0,089941	0	0,705667	0	0	0,6265381	0,780385	2,39750792
GSM2261543	0	0,170766	0	0,092437	0	0,736797	0	0	0,7034297	0,717266	2,799353411
GSM2261544	0	0,20648	0	0,168306	0	0,590838	0,03437534	0	0,5703643	0,821555	1,576466944
GSM2261547	0	0,149637	0	0,119766	0	0,730597	0	0	0,6249725	0,780146	2,711917793
GSM2261549	0	0,106643	0	0,12249	0	0,770868	0	0	0,7189782	0,699516	3,36429062
GSM2261550	0	0,136431	0	0,125728	0	0,737842	0	0	0,6567303	0,75483	2,814488661
GSM2261552	0	0,194697	0	0,12945	0	0,675853	0	0	0,6039521	0,797317	2,085017629
GSM2261567	0	0,145612	0	0,16144	0	0,678378	0,01457062	0	0,5510153	0,833665	2,2093295
GSM2261568	0	0,101089	0,025413	0,088525	0	0,784972	0	0	0,6969537	0,718482	3,650566047
GSM2261569	0	0,159695	0	0,149374	0	0,690931	0	0	0,619177	0,785402	2,235519279
GSM2261570	0	0,094101	0,003162	0,064013	0	0,838723	0	0	0,7388039	0,675543	5,200528963
GSM2261571	0	0,231641	0	0,11844	0	0,649919	0	0	0,6126945	0,792715	1,856479117
GSM2261574	0	0,137161	0	0,121307	0	0,741533	0	0	0,7195002	0,702351	2,868959263
GSM2261586	0	0,161956	0	0,12844	0	0,709605	0	0	0,5840615	0,810819	2,443580797
GSM2261597	0	0,158991	0	0,098579	0	0,74243	0	0	0,6484882	0,761643	2,882444077
GSM2261598	0,00042	0,208781	0	0,137174	0	0,653625	0	0	0,4562617	0,894063	1,889334584
GSM2261600	0	0,158242	0	0,147882	0	0,693876	0	0	0,54886	0,835383	2,266646925
GSM2261602	0	0,171838	0	0,082983	0	0,74518	0	0	0,6613598	0,751422	2,924331403
GSM2261603	0	0,150681	0,018927	0,050152	0	0,78024	0	0	0,7009871	0,71586	3,550428723
GSM2261614	0	0,146845	0,002556	0,181526	0	0,623766	0,04530739	0	0,503457	0,864213	1,884907122
GSM2261615	0	0,140762	0,017006	0,063603	0,00219	0,776439	0	0	0,6499408	0,759605	3,507413289
GSM2261617	0	0,184059	0	0,11798	0	0,697961	0	0	0,6380871	0,771673	2,310831118
GSM2261619	0,000832	0,325888	0	0,114077	0	0,559203	0	0	0,4724567	0,882035	1,271018653
GSM2261620	0	0,148149	0	0,184496	0	0,667355	0	0	0,5213626	0,85329	2,006206867
GSM2261622	0	0,240823	0,060515	0,071484	0	0,627178	0	0	0,7075634	0,7283	1,682246079
GSM2261629	0	0,091182	0	0,112059	0,000623	0,653883	0,142253	0	0,6940832	0,723605	3,217276975
GSM2261633	0	0,144434	0	0,05522	0	0,800346	0	0	0,7726248	0,646009	4,008673665
GSM2261634	0	0,17637	0	0,163359	0	0,66027	0	0	0,6579396	0,758588	1,943517415
GSM2261641	0	0,225333	0	0,129335	0	0,640993	0,00433863	0	0,6537476	0,764259	1,807299455
GSM2261644	0	0,093459	0,001163	0,14743	0,008754	0,749193	0	0	0,7377433	0,684149	3,095162555
GSM2261647	0	0,112137	0,01101	0,068804	0,00843	0,79962	0	0	0,7478752	0,670047	4,165763842
GSM2261650	0	0,170686	0	0,163949	0	0,665365	0	0	0,6768178	0,744034	1,988326688
GSM2261655	0	0,145892	0,0495	0,1246	0	0,680008	0	0	0,7089821	0,718333	2,125073781
GSM2261657	0	0,163423	0	0,177986	0	0,633554	0,02503801	0	0,6324306	0,777584	1,855708019
GSM2261658	0,012305	0,101808	0,064098	0,125576	0	0,696213	0	0	0,7598601	0,674082	2,388529262
GSM2261661	0	0,189945	0	0,148087	0	0,661968	0	0	0,5624336	0,825973	1,958296314
GSM2261673	0	0,1336	0,007549	0,13756	0	0,721292	0	0	0,6910543	0,727982	2,587980596
GSM2261674	0	0,10733	0,076448	0,048979	0	0,767243	0	0	0,757131	0,665799	3,296325584
GSM2261680	0	0,205973	0	0,112604	0	0,681423	0	0	0,6564094	0,759332	2,138960044
GSM2261689	0	0,119397	0	0,077659	0	0,802944	0	0	0,754566	0,66326	4,074698985
GSM2261691	0	0,167516	0,010399	0,093608	0	0,728477	0	0	0,7434541	0,684038	2,682929916
GSM2261708	0	0,188898	0,011434	0,105754	0	0,693914	0	0	0,6697864	0,748173	2,267052974
GSM2261709	0	0,147189	0	0,112039	0	0,740772	0	0	0,7881395	0,640714	2,85760284
GSM2261710	0	0,201008	0	0,181053	0	0,617938	0	0	0,6561942	0,762968	1,617378206
GSM2261716	0	0,225324	0	0,190022	0	0,567097	0,01755656	0	0,6699962	0,757205	1,365360523
GSM2261718	0	0,188391	0	0,138204	0	0,673405	0	0	0,5448257	0,837912	2,061895126
GSM2261719	0	0,156635	0	0,112464	0	0,730901	0	0	0,6864891	0,731474	2,716109163
GSM2261725	0	0,199659	0	0,168378	0	0,631963	0	0	0,7281128	0,709278	1,717116152
GSM2261729	0	0,116519	0	0,102284	0	0,781196	0	0	0,6980232	0,717738	3,570308047
GSM2261735	0	0,156026	0	0,085207	0	0,758767	0	0	0,6899381	0,726738	3,145375238
GSM2261738	0	0,20748	0	0,146503	0	0,646018	0	0	0,6182895	0,788411	1,824999472
GSM2261739	0	0,122053	0,02076	0,058868	0,005126	0,793193	0	0	0,7738673	0,64559	3,932906164
GSM2261742	0	0,287882	0,046096	0,113355	0	0,538845	0,01382284	0	0,6714076	0,76239	1,204573687
GSM2261746	0	0,176738	0	0,068908	0	0,754355	0	0	0,7239948	0,69799	3,070909595
GSM2261747	0	0,204641	0	0,121797	0,004624	0,616644	0,05229479	0	0,7017316	0,729321	1,889009856
GSM2261750	0	0,163288	0	0,086482	0,001736	0,748493	0	0	0,720737	0,701181	2,99672266
GSM2261753	0	0,181179	0	0,097352	0	0,719926	0,00154348	0	0,7059125	0,716926	2,584723697
GSM2261760	0	0,16529	0,012247	0,094495	0	0,727968	0	0	0,6865579	0,731971	2,676032059
GSM2261762	0	0,158146	0	0,112077	0	0,729777	0	0	0,6734128	0,742204	2,700647818
GSM2261765	0	0,140076	0	0,1169	0	0,743024	0	0	0,733566	0,68999	2,891421329
GSM2261775	0	0,146415	0	0,130954	0	0,722632	0	0	0,6435341	0,765933	2,605314384
GSM2261776	0	0,172596	0	0,144557	0	0,682847	0	0	0,6908382	0,7321	2,153053946
GSM2261780	0	0,186705	0	0,068003	0	0,745292	0	0	0,7174046	0,704861	2,926063061
GSM2261782	0	0,24893	0,005447	0,13453	0	0,611093	0	0	0,4662782	0,886134	1,57130584
GSM2261791	0	0,114728	0,087899	0,126257	0	0,665747	0,00536789	0	0,5710784	0,8197	2,024256825
GSM2261798	0	0,188973	0	0,061509	0,002385	0,747133	0	0	0,6995686	0,719939	2,982779981
GSM2261800	0	0,197009	0,037746	0,140942	0	0,468515	0,15578794	0	0,4853692	0,873536	1,247053937
GSM2261801	0	0,186019	0	0,113735	0	0,700246	0	0	0,6421006	0,768612	2,336068153
GSM2261802	0	0,151409	0,004436	0,116248	0	0,727907	0	0	0,7054236	0,716003	2,675216488
GSM2261805	0	0,198959	0	0,093941	0	0,7071	0	0	0,7175217	0,70924	2,414135333
GSM2261807	0	0,195158	0	0,128153	0	0,669543	0,00714621	0	0,6797794	0,742184	2,07089579
GSM2261812	0	0,226868	0,053414	0,072818	0	0,6469	0	0	0,7691784	0,677958	1,832060359
GSM2261815	0	0,100854	0,044507	0,10161	0	0,753028	0	0	0,742107	0,680992	3,049048096
GSM2261816	0	0,224956	0	0,139019	0	0,624843	0,01118232	0	0,5192649	0,854142	1,716718827

GSM2261817	0	0,193726	0	0,153745	0	0,652529	0	0	0,624883	0,78322	1,877934934
GSM2261821	0	0,209901	0	0,104526	0	0,685573	0	0	0,6649379	0,752769	2,180387917
GSM2261823	0	0,108136	0	0,078405	0	0,759125	0,05433488	0	0,6912174	0,723384	4,06949819
GSM2261824	0	0,185372	0	0,14195	0	0,672678	0	0	0,761966	0,676645	2,05509733
GSM2261826	0	0,173539	0,000649	0,130322	0	0,69549	0	0	0,5774893	0,815546	2,283966087
GSM2261828	0	0,19188	0	0,113514	0	0,692464	0,00214123	0	0,5859779	0,809788	2,267443468
GSM2261830	0	0,228847	0	0,105976	0	0,665177	0	0	0,6520148	0,764129	1,986652337
GSM2261832	0	0,201448	0	0,097029	0	0,701523	0	0	0,710528	0,715615	2,350341611
GSM2261834	0	0,16015	0	0,109364	0	0,730486	0	0	0,6841324	0,733506	2,710381869
GSM2261839	0	0,192769	0	0,110797	0	0,655187	0,0412472	0	0,547526	0,836293	2,15830096
GSM2261842	0	0,173001	0	0,126845	0	0,700154	0	0	0,5938384	0,803884	2,33504985
GSM2261844	0	0,228717	0	0,130643	0	0,640641	0	0	0,5313439	0,846669	1,782728933
GSM2261851	0	0,079254	0,08208	0,097671	0	0,740995	0	0	0,6868696	0,72988	2,860923307
GSM2261853	0	0,171899	0	0,065834	0	0,762267	0	0	0,7232117	0,69778	3,206407543
GSM2261856	0	0,185987	0	0,119909	0	0,694103	0	0	0,7223113	0,706458	2,269076817
GSM2261858	0	0,174577	0,006876	0,105242	0	0,713305	0	0	0,7111032	0,713328	2,488023011
GSM2261859	0	0,232963	0	0,063881	0	0,703156	0	0	0,6777708	0,742076	2,368770618
GSM2261860	0	0,202941	0	0,043731	0,005148	0,746352	0,00182804	0	0,7528315	0,673469	3,025686209
GSM2261861	0	0,181267	0	0,152629	0	0,666104	0	0	0,6595063	0,757269	1,994947748
GSM2261866	0	0,070747	0,027732	0,149695	0	0,751825	0	0	0,6519516	0,757564	3,029415945
GSM2261867	0	0,261097	0	0,126076	0	0,612827	0	0	0,6957062	0,738036	1,582825455
GSM2261875	0	0,129831	0,045689	0,10187	0	0,72261	0	0	0,6859647	0,732506	2,605036404
GSM2261876	0	0,169683	0,000836	0,173601	0	0,627794	0,02808699	0	0,5667229	0,822968	1,82435113
GSM2261877	0	0,198972	0	0,114993	0	0,686035	0	0	0,6827999	0,738862	2,185068552
GSM2261888	0	0,138992	0	0,054747	0	0,806262	0	0	0,7706919	0,647065	4,161601346
GSM2261889	0	0,143894	0,014381	0,071638	0	0,770088	0	0	0,7127898	0,705999	3,349483183
GSM2261891	0	0,225589	0	0,137809	0	0,581511	0,05509086	0	0,5101323	0,859566	1,600201767
GSM2261893	0	0,146965	0,00406	0,13984	0	0,709136	0	0	0,6208022	0,783645	2,438027162
GSM2261894	0	0,1722	0	0,119826	0	0,707974	0	0	0,6070918	0,794179	2,424357129
GSM2261907	0	0,12399	0,029303	0,101709	0	0,744998	0	0	0,7490567	0,676068	2,921532165
GSM2261911	0	0,147075	0	0,100718	0	0,752206	0	0	0,7107457	0,709194	3,035616185
GSM2261914	0	0,167555	0	0,123046	0	0,709399	0	0	0,684359	0,734993	2,441140515
GSM2261915	0	0,14301	0,032078	0,147155	0	0,677757	0	0	0,5396205	0,841234	2,103245425
GSM2261937	0	0,13465	0,025118	0,111557	0	0,728674	0	0	0,7127478	0,709715	2,685609807
GSM2261941	0	0,155304	0,033195	0,079938	0	0,731563	0	0	0,7183237	0,705302	2,725265416
GSM2261946	0	0,162993	0,00068	0,164319	0	0,672008	0	0	0,6429378	0,768668	2,048850834
GSM2261949	0	0,218982	0,015686	0,090642	0	0,674689	0	0	0,5016539	0,866684	2,073981601
GSM2261954	0	0,132083	0	0,158735	0	0,709182	0	0	0,6440198	0,765722	2,438576752
GSM2261956	0	0,129075	0,127628	0,02952	0,000375	0,713402	0	0	0,718113	0,707553	2,492469967
GSM2261960	0	0,08197	0,027086	0,167397	0	0,723547	0	0	0,5057173	0,86672	2,617251709
GSM2261961	0	0,137591	0	0,104738	0	0,728676	0,02899597	0	0,7044439	0,715234	3,006978469
GSM2261965	0	0,205791	0,039871	0,101432	0	0,652907	0	0	0,6105508	0,793961	1,881070717
GSM2261968	0,004099	0,169548	0	0,124926	0	0,701427	0	0	0,6835988	0,73641	2,381968441
GSM2261969	0	0,147394	0,0043	0,079341	0	0,768966	0	0	0,7311787	0,689554	3,328357909
GSM2261970	0	0,255984	0,005494	0,115325	0	0,556762	0,06643399	0	0,5140558	0,857494	1,477590123
GSM2261972	0	0,177467	0	0,201372	0	0,621161	0	0	0,5454493	0,836913	1,639644406
GSM2261974	0	0,237115	0,032166	0,068144	0	0,662574	0	0	0,7185221	0,715294	1,963617037
GSM2261979	0	0,14362	0,027744	0,10573	0	0,691333	0,03157318	0	0,713385	0,711836	2,494937238
GSM2261982	0	0,195999	0	0,125305	0	0,678696	0	0	0,7346871	0,698653	2,112316445
GSM2261983	0	0,158803	0,031672	0,085514	0	0,712669	0,01134311	0	0,6634404	0,751204	2,582240986
GSM2261984	0	0,188233	0	0,177156	0	0,634612	0	0	0,5344278	0,844215	1,73681414
GSM2261987	0	0,119325	0,016241	0,091283	0	0,773151	0	0	0,7415587	0,679193	3,408215243
GSM2261993	0,004357	0,151375	0,046709	0,054348	0,003449	0,703468	0,03629455	0	0,7283684	0,698164	2,786773489
GSM2261994	0	0,230897	0	0,071277	0	0,697827	0	0	0,7397365	0,692878	2,309358465
GSM2261995	0	0,176784	0	0,081936	0	0,74128	0	0	0,6949044	0,724153	2,86518535
GSM2261997	0	0,260942	0	0,163896	0	0,575161	0	0	0,5549027	0,832474	1,353835091
GSM2261999	0	0,133805	0	0,112196	0	0,753999	0	0	0,7290365	0,692671	3,065017896
GSM2262000	0	0,18693	0,00024	0,053589	0	0,759241	0	0	0,7489936	0,675099	3,153527618
GSM2262005	0	0,213361	0	0,122805	0	0,663835	0	0	0,5951274	0,804057	1,974726282
GSM2262006	0	0,205565	0	0,131772	0	0,658305	0,00435918	0	0,6897073	0,736055	1,95147897
GSM2262008	0	0,234173	0,001787	0,180427	0	0,580523	0,00308858	0	0,556377	0,830966	1,394188648
GSM2262009	0	0,112518	0	0,207332	0	0,68015	0	0	0,6835923	0,73567	2,126461425
GSM2262010	0	0,230095	0,004977	0,123413	0	0,641516	0	0	0,5200665	0,853894	1,789521353
GSM2262011	0,005287	0,193694	0	0,127315	0	0,673703	0	0	0,6763831	0,744832	2,098703358
GSM2262012	0	0,230004	0	0,09561	0	0,674387	0	0	0,7257447	0,707491	2,071129047
GSM2262019	0	0,12479	0,034003	0,163072	0	0,678135	0	0	0,7073556	0,718565	2,106891078
GSM2262027	0	0,120715	0,011598	0,102953	0	0,764735	0	0	0,6917829	0,724305	3,250518057
GSM2262029	0	0,196632	0	0,101301	0,003439	0,698628	0	0	0,6572141	0,757457	2,344912372
GSM2262037	0	0,163587	0	0,100261	0	0,736152	0	0	0,7336602	0,691317	2,790054175
GSM2262043	0	0,134659	0,043902	0,085415	0	0,736025	0	0	0,7586294	0,669232	2,788234021
GSM2262045	0	0,248861	0	0,119268	0	0,63187	0	0	0,6768382	0,749333	1,716435011
GSM2262047	0	0,159817	0	0,04577	0,001837	0,792576	0	0	0,7012707	0,71486	3,855178674
GSM2262050	0	0,167239	0,056197	0,077371	0,003004	0,696188	0	0	0,6140872	0,789565	2,314400065
GSM2262052	0,00032	0,121807	0,021757	0,090624	0	0,765492	0	0	0,6558812	0,754658	3,268705373
GSM2262054	0	0,230444	0	0,116031	0	0,653525	0	0	0,5142247	0,857892	1,886212648
GSM2262055	0	0,119133	0,027089	0,039909	0	0,813869	0	0	0,7615344	0,655312	4,372562529
GSM2262057	0	0,219357	0	0,122012	0	0,658631	0	0	0,6577947	0,760116	1,929381943

GSM2262062	0	0,096681	0	0,153743	0	0,696527	0,05304857	0	0,6038317	0,795828	2,781387911
GSM2262066	0	0,226509	0	0,131075	0,000939	0,570549	0,07092908	0	0,5677706	0,823883	1,595567531
GSM2262073	0	0,205424	0	0,120538	0	0,674039	0	0	0,5487578	0,835464	2,067847233
GSM2262074	0	0,193136	0	0,177353	0	0,629511	0	0	0,5439876	0,838046	1,699136341
GSM2262076	0	0,16022	0,019509	0,125529	0	0,694742	0	0	0,6328226	0,775458	2,27591375
GSM2262081	0	0,199612	0	0,10778	0	0,692607	0	0	0,5765995	0,816494	2,253167352
GSM2262084	0	0,145043	0	0,165105	0,010057	0,626124	0,05367097	0	0,5611021	0,826464	2,018789873
GSM2262085	0	0,240241	0	0,14558	0	0,610644	0,00353472	0	0,4387128	0,902367	1,582714273
GSM2262093	0	0,201963	0	0,096555	0	0,672356	0,02912571	0	0,6138755	0,790275	2,25231449
GSM2262096	0	0,205437	0,03153	0,13794	0	0,625093	0	0	0,6629221	0,758827	1,667325055
GSM2262099	0	0,156556	0	0,172261	0	0,671183	0	0	0,7304102	0,701129	2,041209813
GSM2262100	0	0,140288	0	0,092735	0	0,766977	0	0	0,6930448	0,723303	3,291429165
GSM2262106	0	0,166288	0	0,138704	0	0,695009	0	0	0,7531293	0,680012	2,278780782
GSM2262111	0	0,230902	0	0,147989	0	0,621109	0	0	0,5624653	0,826703	1,639281464
GSM2262115	0	0,135489	0,062649	0,126715	0	0,675147	0	0	0,6774548	0,743027	2,078318834
GSM2262128	0	0,138198	0,002238	0,19484	0	0,664724	0	0	0,5995547	0,799633	1,982615287
GSM2262130	0	0,193522	0	0,138384	0	0,668095	0	0	0,5341155	0,844985	2,012906436
GSM2262132	0	0,109854	0,043329	0,097779	0	0,749039	0	0	0,6513184	0,758777	2,984676433
GSM2262135	0	0,170766	0	0,176546	0	0,652688	0	0	0,489886	0,873154	1,879257803
GSM2262139	0	0,180417	0	0,089421	0	0,730162	0	0	0,6417559	0,767662	2,705930282
GSM2262142	0	0,264183	0	0,142861	0	0,548905	0,04405031	0	0,4999244	0,865657	1,348514029
GSM2262145	0	0,219542	0	0,083194	0,004729	0,692536	0	0	0,6285738	0,779558	2,287593303
GSM2262151	0	0,145468	0	0,081049	0	0,773483	0	0	0,6576026	0,753243	3,414680999
GSM2262156	0	0,158417	0,001864	0,09113	0	0,74859	0	0	0,7392335	0,684807	2,977562221
GSM2262165	0	0,158751	0,006322	0,122307	0	0,71262	0	0	0,7312037	0,696201	2,479714204
GSM2262167	0	0,213761	0	0,109483	0	0,676756	0	0	0,6034528	0,798001	2,093642023
GSM2262169	0,00294	0,33614	0	0,09493	0,005353	0,559139	0,00149729	0	0,5471074	0,839169	1,297096601
GSM2262170	0	0,177357	0	0,140713	0	0,68193	0	0	0,6070441	0,794688	2,143958265
GSM2262174	0	0,177256	0	0,10991	0	0,712834	0	0	0,662753	0,752008	2,482305007
GSM2262178	0	0,236864	0	0,172667	0	0,574406	0,01606221	0	0,638105	0,778856	1,402593455
GSM2262181	0	0,189438	0	0,057883	0	0,752678	0	0	0,6073107	0,794332	3,043316486
GSM2262182	0	0,161459	0	0,139941	0	0,698601	0	0	0,621885	0,783263	2,317857354
GSM2262188	0	0,251652	0	0,080716	0	0,635475	0,03215668	0	0,5537187	0,832796	1,911960146
GSM2262189	0	0,223024	0	0,155236	0	0,62174	0	0	0,5365041	0,843142	1,643686363
GSM2262190	0	0,093263	0,062904	0,149232	0	0,637018	0,0575827	0	0,6570659	0,758232	2,085856675
GSM2262198	0	0,236814	0	0,17428	0	0,566673	0,02223289	0	0,5602972	0,828816	1,378448707
GSM2262199	0	0,092289	0,001044	0,208533	0	0,698134	0	0	0,6940298	0,725549	2,312732839
GSM2262202	0	0,16383	0	0,058256	0	0,777913	0	0	0,6848869	0,730159	3,502749446
GSM2262206	0	0,189711	0	0,107178	0	0,703111	0	0	0,5766977	0,816367	2,368259263
GSM2262207	0	0,16107	0	0,10178	0	0,73715	0	0	0,6350366	0,772413	2,804450774
GSM2262217	0	0,142452	0,005568	0,115566	0	0,736413	0	0	0,6238621	0,780874	2,793818514
GSM2262218	0	0,167407	0	0,141928	0	0,690665	0	0	0,6709808	0,746732	2,232740921
GSM2262226	0	0,158998	0	0,182178	0	0,658824	0	0	0,5869588	0,808821	1,931035401
GSM2262233	0	0,173665	0	0,061206	0	0,747852	0,01727672	0	0,7132285	0,707159	3,184101365
GSM2262234	0	0,199921	0,004383	0,061753	0	0,733943	0	0	0,6488373	0,762354	2,758594722
GSM2262236	0	0,204646	0	0,107403	0	0,687951	0	0	0,628414	0,77961	2,204627616
GSM2262240	0	0,157204	0	0,092728	0	0,750068	0	0	0,7149796	0,705926	3,001096172
GSM2262244	0	0,240333	0	0,095283	0	0,664384	0	0	0,5457269	0,837824	1,979595782
GSM2262247	0	0,163253	0	0,107885	0,004423	0,72444	0	0	0,6394543	0,769293	2,671849874
GSM2262248	0	0,205312	0,013374	0,09682	0	0,684494	0	0	0,5976597	0,801903	2,16950935
GSM2262255	0	0,238827	0	0,129486	0	0,619571	0,01211631	0	0,5023066	0,864724	1,682186983
GSM2262260	0	0,146955	0	0,057137	0	0,795908	0	0	0,6993803	0,71623	3,899739185
GSM2262262	0	0,196145	0	0,091013	0	0,712842	0	0	0,612687	0,790369	2,482403027
GSM2262269	0	0,175093	0	0,135361	0	0,689546	0	0	0,58793	0,808169	2,221090287
GSM2262271	0	0,242473	0	0,132158	0	0,62537	0	0	0,6198505	0,789072	1,66929878
GSM2262272	0	0,271165	0	0,122806	0	0,565204	0,04082502	0	0,498744	0,866602	1,434634379
GSM2262273	0	0,199922	0	0,098559	0	0,701519	0	0	0,6009677	0,799138	2,350299204
GSM2262277	0	0,100499	0,002396	0,15802	0	0,697412	0,04167353	0	0,7229883	0,700754	2,672951926
GSM2262278	0	0,196644	0	0,140868	0	0,662488	0	0	0,5990817	0,80103	1,962859294
GSM2262283	0	0,192559	0	0,11304	0	0,691605	0,00279631	0	0,625889	0,781065	2,263114326
GSM2262284	0	0,248529	0	0,141925	0	0,609546	0	0	0,4761095	0,880243	1,561118924
GSM2262285	0	0,229017	0	0,132078	0	0,638096	0,00080943	0	0,7018196	0,729872	1,767114141
GSM2262286	0	0,176824	0	0,095593	0	0,727583	0	0	0,6686523	0,746515	2,67083948
GSM2262287	0	0,261174	0	0,079744	0	0,659082	0	0	0,6023634	0,80012	1,933258106
GSM2262288	0	0,14217	0	0,071158	0	0,786672	0	0	0,6996908	0,716361	3,687620179
GSM2262289	0	0,143467	0	0,116048	0	0,728509	0,01197653	0	0,6900201	0,728091	2,807194963
GSM2262295	0	0,242387	0	0,18499	0	0,48563	0,08699322	0	0,4281832	0,905146	1,136303197
GSM2262298	0	0,231609	0	0,193496	0	0,553862	0,02103301	0	0,4298007	0,905622	1,302884559
GSM2262302	0	0,290283	0	0,142628	0	0,497108	0,06998037	0	0,5142929	0,857867	1,148289879
GSM2262306	0	0,194828	0	0,094363	0	0,710808	0	0	0,6251911	0,781085	2,457915122
GSM2262309	0	0,180377	0	0,123331	0	0,690914	0,00537819	0	0,5864123	0,809329	2,274925785
GSM2262310	0	0,179031	0	0,125664	0	0,695305	0	0	0,630366	0,777411	2,28197322
**average**	**0,000137**	**0,177508**	**0,008109**	**0,116807**	**0,000344**	**0,689965**	**0,00713073**				

SES_CIBERSORTx_GSE85217_SHH

Sample	Bcells	TCD4	TCD8	Tgd	NK	MoMaDC	granulocytes	P-value	Correlation	RMSE		Tab
GSM2261538	0	0,861694	0,002827	0,380422	0	2,9847599	0	0	0,6265381	0,780385		0,864521
GSM2261543	0	1,425353	0	0,771555	0	6,1499223	0	0	0,7034297	0,717266		1,425353
GSM2261544	0	0,581533	0	0,47402	0	1,6640443	0,0968151	0	0,5703643	0,821555		0,581533
GSM2261547	0	1,007144	0	0,806094	0	4,9173509	0	0	0,6249725	0,780146		1,007144
GSM2261549	0	0,814156	0	0,935139	0	5,8851358	0	0	0,7189782	0,699516		0,814156
GSM2261550	0	1,312535	0	1,209569	0	7,098433	0	0	0,6567303	0,75483		1,312535
GSM2261552	0	0,741504	0	0,493011	0	2,5739848	0	0	0,6039521	0,797317		0,741504
GSM2261567	0	0,529225	0	0,58675	0	2,4655549	0,0529567	0	0,5510153	0,833665		0,529225
GSM2261568	0	0,625436	0,157232	0,547701	0	4,8566012	0	0	0,6969537	0,718482		0,782668
GSM2261569	0	0,487019	0	0,455545	0	2,1071181	0	0	0,619177	0,785402		0,487019
GSM2261570	0	1,365022	0,045875	0,928564	0	12,166434	0	0	0,7388039	0,675543		1,410897
GSM2261571	0	1,474163	0	0,753755	0	4,1360829	0	0	0,6126945	0,792715		1,474163
GSM2261574	0	0,931295	0	0,823649	0	5,0348643	0	0	0,7195002	0,702351		0,931295
GSM2261586	0	0,489249	0	0,388002	0	2,1436349	0	0	0,5840615	0,810819		0,489249
GSM2261597	0	1,123155	0	0,696387	0	5,2447279	0	0	0,6484882	0,761643		1,123155
GSM2261598	0,002197	1,09247	0	0,717777	0	3,4201632	0	0	0,4562617	0,894063		1,09247
GSM2261600	0	0,877487	0	0,820041	0	3,8476965	0	0	0,54886	0,835383		0,877487
GSM2261602	0	0,857456	0	0,414077	0	3,7183848	0	0	0,6613598	0,751422		0,857456
GSM2261603	0	1,098223	0,137947	0,365528	0	5,686713	0	0	0,7009871	0,71586		1,23617
GSM2261614	0	0,185541	0,003229	0,229361	0	0,7881388	0,0572467	0	0,503457	0,864213		0,18877
GSM2261615	0	0,744322	0,089925	0,336323	0,011578	4,105673	0	0	0,6499408	0,759605		0,834247
GSM2261617	0	0,620196	0	0,39754	0	2,3518152	0	0	0,6380871	0,771673		0,620196
GSM2261619	0,003152	1,233824	0	0,4319	0	2,1171661	0	0	0,4724567	0,882035		1,233824
GSM2261620	0	0,620888	0	0,773222	0	2,7968725	0	0	0,5213626	0,85329		0,620888
GSM2261622	0	1,249144	0,313891	0,370788	0	3,2531657	0	0	0,7075634	0,7283		1,563035
GSM2261629	0	0,58804	0	0,722679	0,004016	4,216946	0,9174013	0	0,6940832	0,723605		0,58804
GSM2261633	0	0,777833	0	0,29738	0	4,3101787	0	0	0,7726248	0,646009		0,777833
GSM2261634	0	1,132371	0	1,048836	0	4,2392134	0	0	0,6579396	0,758588		1,132371
GSM2261641	0	0,57042	0	0,327406	0	1,6226417	0,010983	0	0,6537476	0,764259		0,57042
GSM2261644	0	0,838675	0,010438	1,322997	0,078556	6,7230318	0	0	0,7377433	0,684149		0,849113
GSM2261647	0	0,884295	0,086824	0,542577	0,066477	6,3057012	0	0	0,7478752	0,670047		0,971119
GSM2261650	0	0,455459	0	0,437481	0	1,7754557	0	0	0,6768178	0,744034		0,455459
GSM2261655	0	0,614902	0,208633	0,525163	0	2,8660848	0	0	0,7089821	0,718333		0,823536
GSM2261657	0	0,412568	0	0,449333	0	1,5994373	0,0632097	0	0,6324306	0,777584		0,412568
GSM2261658	0,079293	0,656027	0,413035	0,809183	0	4,4862448	0	0	0,7598601	0,674082		1,069062
GSM2261661	0	0,713285	0	0,556098	0	2,4858273	0	0	0,5624336	0,825973		0,713285
GSM2261673	0	0,554765	0,031345	0,571209	0	2,9951182	0	0	0,6910543	0,727982		0,58611
GSM2261674	0	0,579495	0,412759	0,264448	0	4,1424984	0	0	0,757131	0,665799		0,992254
GSM2261680	0	0,423434	0	0,231488	0	1,4008517	0	0	0,6564094	0,759332		0,423434
GSM2261689	0	1,143229	0	0,743583	0	7,6881904	0	0	0,754566	0,66326		1,143229
GSM2261691	0	1,026867	0,063743	0,573812	0	4,4655269	0	0	0,7434541	0,684038		1,09061
GSM2261708	0	0,91692	0,055502	0,513336	0	3,3682917	0	0	0,6697864	0,748173		0,972422
GSM2261709	0	1,909561	0	1,453543	0	9,6104178	0	0	0,7881395	0,640714		1,909561
GSM2261710	0	0,779692	0	0,702288	0	2,3969223	0	0	0,6561942	0,762968		0,779692
GSM2261716	0	0,843942	0	0,71172	0	2,1240388	0,0657574	0	0,6699962	0,757205		0,843942
GSM2261718	0	0,37179	0	0,272745	0	1,3289628	0	0	0,5448257	0,837912		0,37179
GSM2261719	0	0,444225	0	0,318953	0	2,0728733	0	0	0,6864891	0,731474		0,444225
GSM2261725	0	0,771665	0	0,650765	0	2,4424774	0	0	0,7281128	0,709278		0,771665
GSM2261729	0	0,755171	0	0,662914	0	5,0630002	0	0	0,6980232	0,717738		0,755171
GSM2261735	0	0,800924	0	0,437391	0	3,8949654	0	0	0,6899381	0,726738		0,800924
GSM2261738	0	0,767277	0	0,541778	0	2,3890252	0	0	0,6182895	0,788411		0,767277
GSM2261739	0	1,251138	0,212812	0,603446	0,052549	8,1308735	0	0	0,7738673	0,64559		1,46395
GSM2261742	0	1,59738	0,255772	0,628978	0	2,9899089	0,0766993	0	0,6714076	0,76239		1,853152
GSM2261746	0	0,703699	0	0,274362	0	3,0035357	0	0	0,7239948	0,69799		0,703699
GSM2261747	0	0,45312	0	0,269686	0,010238	1,3653866	0,1157923	0	0,7017316	0,729321		0,45312
GSM2261750	0	0,814655	0	0,431466	0,008663	3,734281	0	0	0,720737	0,701181		0,814655
GSM2261753	0	0,930271	0	0,499857	0	3,696485	0,007925	0	0,7059125	0,716926		0,930271
GSM2261760	0	0,721081	0,053428	0,412237	0	3,1757708	0	0	0,6865579	0,731971		0,774509
GSM2261762	0	0,616858	0	0,437163	0	2,8465392	0	0	0,6734128	0,742204		0,616858
GSM2261765	0	0,824705	0	0,688253	0	4,3745997	0	0	0,733566	0,68999		0,824705
GSM2261775	0	0,70249	0	0,62831	0	3,467153	0	0	0,6435341	0,765933		0,70249
GSM2261776	0	0,698272	0	0,584836	0	2,7626024	0	0	0,6908382	0,7321		0,698272
GSM2261780	0	0,871013	0	0,317246	0	3,4769205	0	0	0,7174046	0,704861		0,871013
GSM2261782	0	0,984831	0,02155	0,532234	0	2,4176344	0	0	0,4662782	0,886134		1,00638
GSM2261791	0	0,332607	0,254828	0,366031	0	1,930062	0,015562	0	0,5710784	0,8197		0,587436
GSM2261798	0	0,747472	0	0,243295	0,009435	2,9552396	0	0	0,6995686	0,719939		0,747472
GSM2261800	0	0,223193	0,042763	0,159675	0	0,5307846	0,1764936	0	0,4853692	0,873536		0,265956
GSM2261801	0	0,492807	0	0,301308	0	1,8551069	0	0	0,6421006	0,768612		0,492807
GSM2261802	0	0,63452	0,01859	0,487166	0	3,0504848	0	0	0,7054236	0,716003		0,65311
GSM2261805	0	0,837211	0	0,395299	0	2,9754459	0	0	0,7175217	0,70924		0,837211
GSM2261807	0	0,692177	0	0,454527	0	2,3747049	0,0253458	0	0,6797794	0,742184		0,692177
GSM2261812	0	2,549381	0,600228	0,818277	0	7,2694061	0	0	0,7691784	0,677958		3,149609
GSM2261815	0	0,646208	0,285173	0,651048	0	4,8249049	0	0	0,742107	0,680992		0,931382
GSM2261816	0	0,403086	0	0,2491	0	1,1196207	0,020037	0	0,5192649	0,854142		0,403086
GSM2261817	0	0,729845	0	0,57922	0	2,4583399	0	0	0,624883	0,78322		0,729845
GSM2261821	0	0,677549	0	0,337405	0	2,2129939	0	0	0,6649379	0,752769		0,677549
GSM2261823	0	0,956969	0	0,693857	0	6,718032	0,4808477	0	0,6912174	0,723384		0,956969

GSM2261824	0	1,015901	0	0,77793	0	3,6864987	0	0	0,761966	0,676645		1,015901
GSM2261826	0	0,566839	0,002119	0,425676	0	2,2717088	0	0	0,5774893	0,815546		0,568958
GSM2261828	0	0,364802	0	0,215812	0	1,3165103	0,0040709	0	0,5859779	0,809788		0,364802
GSM2261830	0	0,918048	0	0,425135	0	2,6684389	0	0	0,6520148	0,764129		0,918048
GSM2261832	0	0,888805	0	0,4281	0	3,0951768	0	0	0,710528	0,715615		0,888805
GSM2261834	0	0,718659	0	0,490758	0	3,2779842	0	0	0,6841324	0,733506		0,718659
GSM2261839	0	0,558274	0	0,320875	0	1,8974688	0,1194549	0	0,547526	0,836293		0,558274
GSM2261842	0	0,536876	0	0,39364	0	2,1727993	0	0	0,5938384	0,803884		0,536876
GSM2261844	0	0,744395	0	0,425197	0	2,0850657	0	0	0,5313439	0,846669		0,744395
GSM2261851	0	0,434829	0,450333	0,535872	0	4,0654677	0	0	0,6868696	0,72988		0,885162
GSM2261853	0	0,662134	0	0,253584	0	2,9361678	0	0	0,7232117	0,69778		0,662134
GSM2261856	0	0,533398	0	0,343892	0	1,9906392	0	0	0,7223113	0,706458		0,533398
GSM2261858	0	1,091132	0,042975	0,657777	0	4,458248	0	0	0,7111032	0,713328		1,134106
GSM2261859	0	1,270456	0	0,348373	0	3,8346344	0	0	0,6777708	0,742076		1,270456
GSM2261860	0	1,177498	0	0,253737	0,029868	4,3304686	0,0106066	0	0,7528315	0,673469		1,177498
GSM2261861	0	0,695283	0	0,585434	0	2,5549647	0	0	0,6595063	0,757269		0,695283
GSM2261866	0	0,149141	0,058462	0,315571	0	1,5849132	0	0	0,6519516	0,757564		0,207604
GSM2261867	0	0,75262	0	0,363417	0	1,7664929	0	0	0,6957062	0,738036		0,75262
GSM2261875	0	0,58689	0,206533	0,460493	0	3,2664953	0	0	0,6859647	0,732506		0,793422
GSM2261876	0	0,281548	0,001387	0,288049	0	1,0416751	0,0466037	0	0,5667229	0,822968		0,282935
GSM2261877	0	0,692088	0	0,39998	0	2,3862433	0	0	0,6827999	0,738862		0,692088
GSM2261888	0	1,10245	0	0,434237	0	6,3950812	0	0	0,7706919	0,647065		1,10245
GSM2261889	0	0,373202	0,037298	0,1858	0	1,9972967	0	0	0,7127898	0,705999		0,4105
GSM2261891	0	0,258318	0	0,157803	0	0,6658772	0,0630835	0	0,5101323	0,859566		0,258318
GSM2261893	0	0,540082	0,014921	0,513898	0	2,6060083	0	0	0,6208022	0,783645		0,555003
GSM2261894	0	0,552193	0	0,384245	0	2,2702602	0	0	0,6070918	0,794179		0,552193
GSM2261907	0	0,568067	0,134255	0,465985	0	3,4132478	0	0	0,7490567	0,676068		0,702322
GSM2261911	0	1,038452	0	0,711141	0	5,3110958	0	0	0,7107457	0,709194		1,038452
GSM2261914	0	0,863827	0	0,634359	0	3,6572829	0	0	0,684359	0,734993		0,863827
GSM2261915	0	0,561574	0,125964	0,577851	0	2,6614251	0	0	0,5396205	0,841234		0,687538
GSM2261937	0	0,61864	0,115402	0,512539	0	3,3478293	0	0	0,7127478	0,709715		0,734042
GSM2261941	0	1,092581	0,233534	0,562375	0	5,1466348	0	0	0,7183237	0,705302		1,326114
GSM2261946	0	0,731192	0,00305	0,737139	0	3,0146406	0	0	0,6429378	0,768668		0,734242
GSM2261949	0	0,721489	0,051683	0,298643	0	2,2229235	0	0	0,5016539	0,866684		0,773172
GSM2261954	0	0,440226	0	0,529055	0	2,363667	0	0	0,6440198	0,765722		0,440226
GSM2261956	0	1,101368	1,089014	0,251884	0,003201	6,0872746	0	0	0,718113	0,707553		2,190382
GSM2261960	0	0,447404	0,14784	0,913676	0	3,9492214	0	0	0,5057173	0,86672		0,595244
GSM2261961	0	0,436697	0	0,332425	0	2,312732	0,0920298	0	0,7044439	0,715234		0,436697
GSM2261965	0	0,458091	0,088752	0,225788	0	1,4533754	0	0	0,6105508	0,793961		0,546844
GSM2261968	0,016887	0,698541	0	0,514699	0	2,8898985	0	0	0,6835988	0,73641		0,698541
GSM2261969	0	0,365316	0,010657	0,196647	0	1,905886	0	0	0,7311787	0,689554		0,375974
GSM2261970	0	0,373956	0,008026	0,168473	0	0,8133491	0,0970505	0	0,5140558	0,857494		0,381983
GSM2261972	0	0,563148	0	0,639003	0	1,9710994	0	0	0,5454493	0,836913		0,563148
GSM2261974	0	1,760579	0,238835	0,505972	0	4,91962	0	0	0,7185221	0,715294		1,999414
GSM2261979	0	0,805105	0,155528	0,592704	0	3,8754802	0,1769933	0	0,713385	0,711836		0,960634
GSM2261982	0	2,241284	0	1,432884	0	7,7610047	0	0	0,7346871	0,698653		2,241284
GSM2261983	0	0,478135	0,09536	0,257472	0	2,1457576	0,0341527	0	0,6634404	0,751204		0,573495
GSM2261984	0	0,363784	0	0,342376	0	1,2264673	0	0	0,5344278	0,844215		0,363784
GSM2261987	0	1,032397	0,14052	0,789776	0	6,6892767	0	0	0,7415587	0,679193		1,172916
GSM2261993	0,018362	0,637958	0,196849	0,229044	0,014535	2,9647116	0,1529605	0	0,7283684	0,698164		0,834807
GSM2261994	0	1,451397	0	0,44804	0	4,3864813	0	0	0,7397365	0,692878		1,451397
GSM2261995	0	0,638324	0	0,295852	0	2,6765864	0	0	0,6949044	0,724153		0,638324
GSM2261997	0	0,635787	0	0,399334	0	1,4013838	0	0	0,5549027	0,832474		0,635787
GSM2261999	0	0,661001	0	0,554254	0	3,7247782	0	0	0,7290365	0,692671		0,661001
GSM2262000	0	0,986371	0,001266	0,282772	0	4,0062675	0	0	0,7489936	0,675099		0,987636
GSM2262005	0	0,395026	0	0,227366	0	1,2290545	0	0	0,5951274	0,804057		0,395026
GSM2262006	0	0,561802	0	0,360128	0	1,7991255	0,0119135	0	0,6897073	0,736055		0,561802
GSM2262008	0	0,902049	0,006886	0,695017	0	2,2362113	0,0118974	0	0,556377	0,830966		0,908934
GSM2262009	0	0,305972	0	0,563799	0	1,8495348	0	0	0,6835923	0,73567		0,305972
GSM2262010	0	0,687087	0,014861	0,368523	0	1,9156307	0	0	0,5200665	0,853894		0,701948
GSM2262011	0,017595	0,644596	0	0,423692	0	2,2420189	0	0	0,6763831	0,744832		0,644596
GSM2262012	0	0,966867	0	0,401915	0	2,8349249	0	0	0,7257447	0,707491		0,966867
GSM2262019	0	0,414417	0,112922	0,54155	0	2,2520329	0	0	0,7073556	0,718565		0,527339
GSM2262027	0	1,272803	0,122283	1,085521	0	8,063259	0	0	0,6917829	0,724305		1,395086
GSM2262029	0	0,610671	0	0,314607	0,01068	2,1696956	0	0	0,6572141	0,757457		0,610671
GSM2262037	0	1,074947	0	0,658823	0	4,8373129	0	0	0,7336602	0,691317		1,074947
GSM2262043	0	0,644092	0,209989	0,408551	0	3,5205146	0	0	0,7586294	0,669232		0,854081
GSM2262045	0	0,716274	0	0,343279	0	1,818654	0	0	0,6768382	0,749333		0,716274
GSM2262047	0	0,977394	0	0,279918	0,011235	4,8471634	0	0	0,7012707	0,71486		0,977394
GSM2262050	0	0,5627	0,189081	0,260327	0,010109	2,3424224	0	0	0,6140872	0,789565		0,751781
GSM2262052	0,001131	0,430524	0,076899	0,320309	0	2,7056096	0	0	0,6558812	0,754658		0,507423
GSM2262054	0	0,743462	0	0,374342	0	2,1084157	0	0	0,5142247	0,857892		0,743462
GSM2262055	0	0,961622	0,218654	0,322139	0	6,5694017	0	0	0,7615344	0,655312		1,180276
GSM2262057	0	0,878675	0	0,488743	0	2,6382724	0	0	0,6577947	0,760116		0,878675
GSM2262062	0	0,613506	0	0,975599	0	4,4199181	0,3366277	0	0,6038317	0,795828		0,613506
GSM2262066	0	0,334687	0	0,193675	0,001387	0,8430376	0,1048042	0	0,5677706	0,823883		0,334687
GSM2262073	0	0,83194	0	0,488163	0	2,7297715	0	0	0,5487578	0,835464		0,83194
GSM2262074	0	0,854326	0	0,784507	0	2,7846014	0	0	0,5439876	0,838046		0,854326
GSM2262076	0	0,501019	0,061007	0,392538	0	2,1725066	0	0	0,6328226	0,775458		0,562026
GSM2262081	0	0,551864	0	0,297978	0	1,9148363	0	0	0,5765995	0,816494		0,551864

GSM2262084	0	0,835655	0	0,95124	0,057942	3,6073663	0,3092212	0	0,5611021	0,826464		0,835655
GSM2262085	0	0,77391	0	0,468972	0	1,9671266	0,0113867	0	0,4387128	0,902367		0,77391
GSM2262093	0	0,863063	0	0,412613	0	2,8732233	0,1244648	0	0,6138755	0,790275		0,863063
GSM2262096	0	0,774213	0,118826	0,519845	0	2,3557372	0	0	0,6629221	0,758827		0,893039
GSM2262099	0	1,016506	0	1,118475	0	4,3579442	0	0	0,7304102	0,701129		1,016506
GSM2262100	0	0,799583	0	0,528552	0	4,3714621	0	0	0,6930448	0,723303		0,799583
GSM2262106	0	1,140013	0	0,950908	0	4,7647501	0	0	0,7531293	0,680012		1,140013
GSM2262111	0	0,778743	0	0,499111	0	2,0947625	0	0	0,5624653	0,826703		0,778743
GSM2262115	0	0,772713	0,357293	0,722669	0	3,8504495	0	0	0,6774548	0,743027		1,130006
GSM2262128	0	0,923555	0,014958	1,302083	0	4,442241	0	0	0,5995547	0,799633		0,938513
GSM2262130	0	0,682958	0	0,488369	0	2,3577717	0	0	0,5341155	0,844985		0,682958
GSM2262132	0	0,841834	0,33204	0,749298	0	5,7400483	0	0	0,6513184	0,758777		1,173874
GSM2262135	0	0,44612	0	0,461222	0	1,7051296	0	0	0,489886	0,873154		0,44612
GSM2262139	0	1,275841	0	0,632351	0	5,163434	0	0	0,6417559	0,767662		1,275841
GSM2262142	0	0,41815	0	0,226121	0	0,8688078	0,0697229	0	0,4999244	0,865657		0,41815
GSM2262145	0	1,137608	0	0,43109	0,024503	3,5885424	0	0	0,6285738	0,779558		1,137608
GSM2262151	0	0,617811	0	0,344219	0	3,285025	0	0	0,6576026	0,753243		0,617811
GSM2262156	0	1,289078	0,015169	0,741546	0	6,091474	0	0	0,7392335	0,684807		1,304246
GSM2262165	0	0,796409	0,031716	0,613576	0	3,5750063	0	0	0,7312037	0,696201		0,828124
GSM2262167	0	0,850267	0	0,435484	0	2,6919031	0	0	0,6034528	0,798001		0,850267
GSM2262169	0,018428	2,106704	0	0,594961	0,03355	3,5043212	0,009384	0	0,5471074	0,839169		2,106704
GSM2262170	0	0,635236	0	0,503991	0	2,4424532	0	0	0,6070441	0,794688		0,635236
GSM2262174	0	0,778862	0	0,482947	0	3,1321965	0	0	0,662753	0,752008		0,778862
GSM2262178	0	1,010754	0	0,736809	0	2,4511211	0,0685411	0	0,638105	0,778856		1,010754
GSM2262181	0	1,265656	0	0,386725	0	5,0287183	0	0	0,6073107	0,794332		1,265656
GSM2262182	0	0,592967	0	0,513942	0	2,5656573	0	0	0,621885	0,783263		0,592967
GSM2262188	0	0,344664	0	0,11055	0	0,8703505	0,044042	0	0,5537187	0,832796		0,344664
GSM2262189	0	1,12128	0	0,78047	0	3,1258807	0	0	0,536504	0,843142		1,12128
GSM2262190	0	0,652982	0,440423	1,044848	0	4,4600888	0,4031657	0	0,6570659	0,758232		1,093405
GSM2262198	0	0,305534	0	0,224854	0	0,7311121	0,0286845	0	0,5602972	0,828816		0,305534
GSM2262199	0	0,638148	0,007217	1,441937	0	4,827374	0	0	0,6940298	0,725549		0,645366
GSM2262202	0	1,264416	0	0,44961	0	6,0038065	0	0	0,6848869	0,730159		1,264416
GSM2262206	0	0,73903	0	0,417516	0	2,7390023	0	0	0,5766977	0,816367		0,73903
GSM2262207	0	0,931751	0	0,588776	0	4,2642429	0	0	0,6350366	0,772413		0,931751
GSM2262217	0	0,379231	0,014824	0,307656	0	1,9604528	0	0	0,6238621	0,780874		0,394055
GSM2262218	0	0,706652	0	0,599098	0	2,9154015	0	0	0,6709808	0,746732		0,706652
GSM2262226	0	0,456091	0	0,522584	0	1,8898553	0	0	0,5869588	0,808821		0,456091
GSM2262233	0	0,915975	0	0,322821	0	3,9444501	0,0911238	0	0,7132285	0,707159		0,915975
GSM2262234	0	1,00371	0,022002	0,310032	0	3,6847774	0	0	0,6488373	0,762354		1,025712
GSM2262236	0	0,990033	0	0,519595	0	3,3281676	0	0	0,628414	0,77961		0,990033
GSM2262240	0	0,744176	0	0,438959	0	3,5507001	0	0	0,7149796	0,705926		0,744176
GSM2262244	0	0,986687	0	0,391182	0	2,7276224	0	0	0,5457269	0,837824		0,986687
GSM2262247	0	0,547707	0	0,361948	0,014838	2,4304614	0	0	0,6394543	0,769293		0,547707
GSM2262248	0	0,592637	0,038605	0,279471	0	1,975801	0	0	0,5976597	0,801903		0,631242
GSM2262255	0	0,46038	0	0,249607	0	1,1943298	0,0233563	0	0,5023066	0,864724		0,46038
GSM2262260	0	0,972855	0	0,378254	0	5,268973	0	0	0,6993803	0,71623		0,972855
GSM2262262	0	0,966362	0	0,448403	0	3,5120175	0	0	0,612687	0,790369		0,966362
GSM2262269	0	0,509396	0	0,393804	0	2,0060889	0	0	0,58793	0,808169		0,509396
GSM2262271	0	1,182078	0	0,644281	0	3,0487388	0	0	0,6198505	0,789072		1,182078
GSM2262272	0	0,700369	0	0,317186	0	1,45982	0,1054436	0	0,498744	0,866602		0,700369
GSM2262273	0	1,203043	0	0,593082	0	4,2214311	0	0	0,6009677	0,799138		1,203043
GSM2262277	0	0,658227	0,015692	1,03497	0	4,5677767	0,2729454	0	0,7229883	0,700754		0,673919
GSM2262278	0	1,037532	0	0,74325	0	3,495426	0	0	0,5990817	0,80103		1,037532
GSM2262283	0	0,522339	0	0,306634	0	1,8760601	0,0075853	0	0,625889	0,781065		0,522339
GSM2262284	0	1,376804	0	0,786238	0	3,3767671	0	0	0,4761095	0,880243		1,376804
GSM2262285	0	0,806072	0	0,464876	0	2,2459098	0,002849	0	0,7018196	0,729872		0,806072
GSM2262286	0	0,986543	0	0,533337	0	4,0593555	0	0	0,6686523	0,746515		0,986543
GSM2262287	0	0,993568	0	0,303366	0	2,5073087	0	0	0,6023634	0,80012		0,993568
GSM2262288	0	1,005162	0	0,503098	0	5,5618901	0	0	0,6996908	0,716361		1,005162
GSM2262289	0	0,779704	0	0,630685	0	3,9592365	0,065089	0	0,6900201	0,728091		0,779704
GSM2262295	0	0,900649	0	0,687374	0	1,8044756	0,3232444	0	0,4281832	0,905146		0,900649
GSM2262298	0	0,364962	0	0,304904	0	0,8727575	0,0331431	0	0,4298007	0,905622		0,364962
GSM2262302	0	0,334741	0	0,164472	0	0,5732412	0,080698	0	0,5142929	0,857867		0,334741
GSM2262306	0	1,509702	0	0,73121	0	5,5079701	0	0	0,6251911	0,781085		1,509702
GSM2262309	0	0,473382	0	0,32367	0	1,8132344	0,0141145	0	0,5864123	0,809329		0,473382
GSM2262310	0	0,434881	0	0,30525	0	1,6889586	0	0	0,630366	0,777411		0,434881
**average**	**0,000704**	**0,788224**	**0,044172**	**0,515585**	**0,002033**	**3,2912066**	**0,024814**					

CIBERSORTx_GSE85217_GP3

Sample	Bcells	TCD4	TCD8	Tgd	NK	MoMaDC	granulocytes	P-value	Correlation	RMSE	ratio mono/T cells
GSM2261539	0	0,339832	0,054648	0,089426	0	0,44303	0,07306385	0	0,3989844	0,919762	0,915530295
GSM2261545	0	0,350243	0	0,132466	0	0,457904	0,05938724	0	0,3805862	0,929204	0,948612796
GSM2261554	0	0,379944	0	0,034962	0,023633	0,515368	0,04609337	0	0,4776851	0,879683	1,242133122
GSM2261555	0	0,325486	0	0,186661	0	0,389968	0,09788521	0	0,3499741	0,943313	0,76143693
GSM2261559	0	0,412242	0	0,187824	0	0,225329	0,17460546	0	0,3653978	0,935674	0,375506286
GSM2261560	0	0,33191	0	0,134926	0	0,471057	0,0621067	0	0,3287346	0,955518	1,009040812
GSM2261561	0	0,351578	0	0,119984	0	0,450108	0,07832994	0	0,484039	0,87573	0,954504909
GSM2261565	0	0,136769	0,126071	0,068912	0	0,668248	0	0	0,459582	0,892669	2,014301316
GSM2261566	0	0,296221	0	0,189286	0	0,424578	0,08991439	0	0,453068	0,891142	0,874504429
GSM2261577	0	0,432194	0	0,143105	0	0,373086	0,05161516	0	0,4174269	0,910485	0,6485091
GSM2261581	0,008076	0,165188	0,02601	0,107482	0	0,683598	0,00964663	0	0,6096076	0,792979	2,288731462
GSM2261590	0	0,312574	0	0,149276	0,00693	0,49282	0,03839971	0	0,5202615	0,854703	1,067058051
GSM2261592	0	0,251439	0	0,137688	0,001603	0,586064	0,02320745	0,77	0,0092904	1,121121	1,506101418
GSM2261593	0	0,297216	0,058527	0,085489	0	0,558768	0	0	0,5594566	0,831396	1,266382738
GSM2261594	0	0,260203	0,032124	0,123528	0	0,512902	0,07124301	0	0,4749077	0,879823	1,233367235
GSM2261601	0	0,344624	0	0,080294	0	0,538522	0,03655952	0	0,4071596	0,918157	1,267352308
GSM2261604	0	0,275713	0	0,209353	0	0,398696	0,11623767	0	0,4135276	0,911729	0,821941678
GSM2261605	0	0,16271	0,010474	0,0593	0,00817	0,759347	0	0	0,6773557	0,737439	3,266235631
GSM2261612	0	0,317746	0	0,107697	0	0,574557	0	0	0,4538985	0,892774	1,350490113
GSM2261613	0	0,242959	0	0,164368	0	0,498378	0,09429467	0	0,5261149	0,850226	1,223533908
GSM2261624	0	0,152042	0,051056	0,058198	0	0,738704	0	0	0,5198403	0,85775	2,827079359
GSM2261625	0	0,278217	0	0,147077	0	0,557783	0,0169222	0	0,4383712	0,900766	1,311522556
GSM2261628	0	0,398793	0	0,11491	0	0,339492	0,14680537	0	0,3927966	0,922276	0,660873675
GSM2261635	0	0,169048	0	0,143838	0	0,687114	0	0	0,5695864	0,820977	2,19605382
GSM2261636	0	0,216632	0,01801	0,108046	0	0,636475	0,02083858	0	0,5957147	0,804431	1,857307139
GSM2261642	0	0,381927	0	0,093951	0	0,416883	0,10723914	0	0,3570411	0,940656	0,876028705
GSM2261643	0,001604	0,323279	0	0,152591	0,00959	0,432005	0,08093031	0	0,4945066	0,869424	0,907821517
GSM2261645	0	0,298898	0,01425	0,14221	0,011164	0,435433	0,09804438	0	0,4886495	0,872298	0,956243571
GSM2261646	0	0,330503	0	0,178681	0,005096	0,366721	0,11899913	0	0,4373522	0,899283	0,720214104
GSM2261649	0	0,333831	0,020026	0,075121	0,07324	0,444648	0,05313396	0	0,4283811	0,90383	1,036529297
GSM2261667	0	0,313744	0	0,108381	0	0,549039	0,02883555	0	0,5680388	0,826357	1,300652425
GSM2261668	0,001652	0,382979	0	0,10146	0,000368	0,411167	0,10237341	0	0,4498989	0,89412	0,848749188
GSM2261675	0	0,244063	0,019386	0,134562	0	0,513049	0,08894087	0	0,5397336	0,842362	1,289033542
GSM2261676	0	0,237753	0,024423	0,06541	0,001729	0,66391	0,0067759	0	0,6575775	0,760446	2,026675046
GSM2261678	0	0,414422	0	0,134173	0	0,355414	0,09599215	0	0,3604477	0,937853	0,647862935
GSM2261700	0	0,268481	0	0,153896	0,013745	0,50312	0,06075909	0	0,4640847	0,885468	1,191163824
GSM2261701	0	0,27398	0	0,11724	0	0,585268	0,02351185	0	0,4202482	0,912164	1,49600697
GSM2261711	0	0,351428	0	0,121071	0,024335	0,460253	0,04291301	0	0,4347814	0,901108	0,974081465
GSM2261720	0	0,253879	0	0,24305	0	0,341421	0,16164997	0	0,4727844	0,880114	0,687063074
GSM2261722	0	0,298674	0	0,168416	0	0,446724	0,08618653	0	0,4635144	0,885779	0,956398062
GSM2261732	0	0,304996	0	0,095435	0	0,59957	0	0	0,5470841	0,838007	1,497312399
GSM2261734	0	0,241233	0	0,132746	0,065052	0,553859	0,00711066	0	0,4908085	0,870181	1,480989675
GSM2261737	0	0,306386	0	0,067542	0,030211	0,587518	0,00834199	0	0,5475147	0,837544	1,571204305
GSM2261743	0	0,216945	0,027244	0,08606	0	0,65321	0,01653952	0	0,5594423	0,828578	1,977926522
GSM2261745	0,000712	0,333977	0,039038	0,116972	0	0,46213	0,04717119	0	0,3947332	0,921967	0,94314958
GSM2261748	0	0,294401	0,040094	0,107592	0	0,442556	0,11535704	0	0,3710685	0,933991	1,001062405
GSM2261756	0	0,158728	0,109792	0,09474	0	0,63369	0,00304971	0	0,6147118	0,791424	1,744454978
GSM2261757	0	0,252921	0,027756	0,088126	0	0,611379	0,01981777	0	0,6266333	0,785615	1,657734581
GSM2261758	0	0,249278	0	0,055956	0,010148	0,661912	0,02270601	0	0,7571407	0,682946	2,168535067
GSM2261763	0	0,318874	0	0,167761	0	0,383852	0,1295128	0	0,555284	0,836656	0,788788628
GSM2261768	0	0,317641	0,014766	0,095397	0,002385	0,533266	0,03654618	0	0,4813294	0,876928	1,246520401
GSM2261793	0,002894	0,325332	0	0,197097	0	0,319744	0,15493243	0	0,4061	0,915005	0,612034152
GSM2261794	0	0,448194	0	0,178843	0	0,2948	0,07816307	0	0,4420648	0,898033	0,470148079
GSM2261797	0	0,365326	0	0,16966	0	0,354266	0,11074816	0	0,3019483	0,965555	0,662196412
GSM2261818	0	0,297251	0	0,175256	0,007319	0,445581	0,07459317	0	0,5039959	0,863666	0,943016337
GSM2261820	0	0,370173	0	0,065905	0,027351	0,45592	0,08065152	0	0,3953465	0,921862	1,045503691
GSM2261840	0,009329	0,399535	0	0,192262	0	0,252195	0,14667882	0	0,4344668	0,901239	0,426151623
GSM2261841	0,009313	0,367566	0	0,165812	0	0,328645	0,12866462	0	0,3987882	0,91862	0,616156801
GSM2261843	0	0,424186	0	0,089011	0	0,415792	0,07101121	0	0,4304192	0,904558	0,810198789
GSM2261845	0	0,295982	0,018299	0,184893	0,029096	0,354921	0,11680898	0	0,4560563	0,889052	0,711015798
GSM2261850	0	0,126795	0,042133	0,189256	0	0,56041	0,08140592	0	0,6079054	0,796139	1,564588765
GSM2261855	0	0,174732	0	0,097558	0	0,72771	0	0	0,6826468	0,73521	2,67255358
GSM2261863	0,021679	0,180178	0,071018	0,062452	0	0,350926	0,31374767	0	0,6454475	0,775556	1,118852247
GSM2261869	0	0,226123	0,053418	0,11884	0	0,571409	0,03021056	0	0,5136137	0,85742	1,434328287
GSM2261874	0	0,218827	0,023953	0,051881	0	0,666497	0,03884268	0	0,5433667	0,839818	2,261912579
GSM2261878	0	0,346315	0	0,129847	0	0,468978	0,05485936	0	0,5493772	0,839652	0,984911395
GSM2261881	0	0,296745	0,012172	0,11196	0	0,543323	0,03579964	0	0,324478	0,961378	1,290930105
GSM2261882	0	0,174231	0,055046	0,046524	0,039693	0,682184	0,00232164	0	0,6098018	0,792681	2,473465046
GSM2261883	0	0,255699	0,007605	0,142193	0	0,594503	0	0	0,4958136	0,868219	1,466107131
GSM2261885	0	0,340019	0	0,098828	0	0,519576	0,04157682	0	0,3977569	0,922261	1,18395727
GSM2261896	0	0,302659	0	0,180198	0	0,403748	0,11339395	0	0,3593559	0,939034	0,836164063
GSM2261900	0	0,298716	0	0,092483	0	0,552782	0,05601917	0	0,4367037	0,902406	1,413046165
GSM2261919	0	0,285846	0,025684	0,160097	0	0,415305	0,1130687	0	0,5163154	0,857292	0,880579299
GSM2261926	0	0,337733	0	0,114109	0	0,486049	0,06210925	0	0,5089006	0,86188	1,075705499

GSM2261927	0	0,291025	0,002423	0,074273	0	0,618982	0,01329702	0	0,507765	0,862172	1,683293109
GSM2261943	0	0,121191	0,036173	0,068977	0	0,773659	0	0	0,7627592	0,659485	3,418113529
GSM2261959	0	0,319036	0,005591	0,125156	0	0,509924	0,04029369	0	0,5181384	0,85615	1,133712884
GSM2261978	0	0,242686	0,081534	0,045494	0	0,630286	0	0	0,6244936	0,786463	1,704793391
GSM2261980	0	0,283319	0,058487	0,144604	0	0,46883	0,0447594	0	0,4364772	0,900136	0,963855221
GSM2261981	0	0,22393	0	0,162563	0,002612	0,513951	0,09694326	0	0,2764317	0,98689	1,329779681
GSM2262014	0	0,301363	0	0,132108	0	0,535871	0,03065814	0	0,5832039	0,817227	1,236232899
GSM2262015	0,00206	0,408096	0	0,100615	0,001825	0,45672	0,0306831	0	0,4678447	0,8851	0,897799501
GSM2262016	0	0,313584	0,008914	0,185557	0	0,388367	0,10357712	0	0,403206	0,916742	0,764418164
GSM2262018	0,000444	0,420561	0,038728	0,114952	0,014566	0,281795	0,12895427	0	0,331119	0,951195	0,490724933
GSM2262021	0	0,254264	0	0,173866	0	0,492382	0,07948882	0	0,3490045	0,947159	1,150075686
GSM2262022	0,002085	0,308082	0	0,225655	0	0,314723	0,14945388	0	0,3748216	0,930949	0,589659541
GSM2262032	0,006476	0,257842	0	0,184268	0	0,515833	0,03558037	0	0,5030862	0,863571	1,166754103
GSM2262036	0	0,248793	0	0,10466	0	0,602479	0,0440675	0	0,5508341	0,834691	1,704550132
GSM2262038	0	0,319202	0,027261	0,075092	0	0,496177	0,0822684	0	0,4909803	0,871788	1,177016648
GSM2262039	0	0,303535	0	0,126904	0	0,558996	0,01056464	0	0,5524251	0,835233	1,29866555
GSM2262048	0	0,341206	0	0,190593	0	0,392609	0,07559145	0	0,3142478	0,960783	0,7382661
GSM2262056	0	0,394557	0	0,096161	0	0,418241	0,09104096	0	0,3890769	0,924869	0,8523054
GSM2262058	0	0,294851	0,075872	0,128354	0	0,425221	0,07570249	0	0,5727282	0,82664	0,852015908
GSM2262065	0	0,211308	0,096665	0,113645	0	0,555936	0,0224464	0	0,3792731	0,932938	1,318578899
GSM2262070	0	0,361743	0	0,132984	0	0,44259	0,06268381	0	0,3669483	0,935705	0,89461442
GSM2262072	0	0,295235	0	0,186857	0,016839	0,3859	0,11516902	0	0,4361594	0,899654	0,80046937
GSM2262075	0	0,370711	0,050569	0,013285	0,053485	0,511951	0	0	0,2995212	0,971566	1,178080499
GSM2262079	0	0,370298	0	0,110101	0,002999	0,447332	0,06926935	0	0,4474734	0,895152	0,931168506
GSM2262087	0	0,34713	0	0,035586	0,107308	0,360875	0,14910101	0	0,3507288	0,942669	0,942933197
GSM2262089	0	0,21549	0,040909	0,195682	0,002151	0,35907	0,18669875	0	0,4970119	0,866783	0,794261212
GSM2262090	0,007957	0,365869	0	0,12584	0	0,38866	0,11167517	0	0,3814708	0,927626	0,790427218
GSM2262092	0,007288	0,39548	0	0,132874	0	0,31997	0,14438751	0	0,412341	0,912428	0,605596523
GSM2262097	0	0,187064	0,20312	0,176769	0	0,339795	0,09325253	0	0,3691071	0,935101	0,59933537
GSM2262101	0	0,386197	0	0,134248	0	0,309754	0,1697999	0	0,3513438	0,941912	0,595170968
GSM2262108	0	0,302165	0,02645	0,135147	0	0,498657	0,03758046	0	0,3579253	0,941664	1,075242196
GSM2262109	0	0,261409	0,045728	0,11189	0,007801	0,474574	0,09859808	0	0,4493838	0,893473	1,13256047
GSM2262110	0	0,194605	0,05218	0,104626	0	0,491097	0,15749101	0	0,5386984	0,842347	1,397499353
GSM2262112	0	0,223619	0,039978	0,178698	0,011466	0,515123	0,03111542	0	0,5390655	0,842145	1,164661145
GSM2262114	0	0,31189	0,00525	0,108101	0,011056	0,487132	0,07657149	0	0,4537366	0,891517	1,145544286
GSM2262118	0	0,372124	0	0,061374	0,005701	0,530414	0,03038659	0	0,4528919	0,893443	1,22356516
GSM2262125	0	0,345088	0,051711	0,122033	0	0,315863	0,16530499	0	0,3519087	0,941429	0,608796701
GSM2262129	0	0,349432	0	0,107024	0	0,523894	0,01965026	0	0,3557817	0,943992	1,147742445
GSM2262133	0	0,342154	0	0,080933	0	0,5607	0,01621314	0	0,4178467	0,912948	1,325258795
GSM2262134	0	0,377987	0	0,069504	0,005375	0,547133	0	0	0,4743297	0,881691	1,222667437
GSM2262137	0	0,444456	0	0,127217	0,005447	0,340655	0,08222439	0	0,3782491	0,929465	0,595889787
GSM2262140	0	0,285376	0,054433	0,126742	0,014192	0,467211	0,05204571	0	0,3892269	0,92452	1,001415039
GSM2262141	0	0,415341	0	0,135811	0	0,392871	0,05597752	0	0,3514334	0,942707	0,71281827
GSM2262143	0	0,374745	0	0,112771	0	0,492074	0,02041012	0	0,3752814	0,932895	1,009348791
GSM2262150	0	0,265947	0	0,06734	0	0,666713	0	0	0,5932932	0,806249	2,000413985
GSM2262153	0	0,421456	0	0,103494	0,002502	0,380366	0,09218122	0	0,4644542	0,887104	0,724576592
GSM2262155	0	0,191178	0	0,067405	0	0,741417	0	0	0,7035265	0,717157	2,86723196
GSM2262157	0	0,272903	0	0,07352	0,016944	0,636633	0	0	0,6416858	0,77414	1,837736354
GSM2262158	0	0,477319	0	0,043997	0,025263	0,369346	0,08407583	0	0,390585	0,924293	0,708488652
GSM2262184	0	0,296921	0,001791	0,113478	0,017404	0,456528	0,11387697	0	0,3519385	0,944067	1,10756694
GSM2262192	0	0,361969	0	0,19305	0	0,304487	0,14049282	0	0,3355429	0,949626	0,548606322
GSM2262195	0,019089	0,129576	0,062056	0,087279	0,005585	0,647026	0,04938896	0	0,6457936	0,767701	2,319831505
GSM2262204	0	0,348078	0	0,149978	0	0,413122	0,0888231	0	0,2990947	0,967973	0,829469366
GSM2262205	0	0,35045	0	0,088199	0	0,498654	0,06269725	0	0,3460754	0,948508	1,136793714
GSM2262208	0	0,249936	0	0,088946	0	0,645986	0,01513198	0	0,5711593	0,821183	1,906229804
GSM2262209	0	0,324151	0	0,124856	0	0,491984	0,0590093	0	0,3876344	0,926527	1,095718664
GSM2262229	0,002918	0,304692	0	0,09296	0,019064	0,552132	0,02823328	0	0,5707688	0,824349	1,38847927
GSM2262249	0	0,374452	0	0,248976	0	0,290524	0,08604792	0	0,4141227	0,911917	0,466011432
GSM2262254	0	0,374033	0	0,10677	0	0,519197	0	0	0,5235446	0,85393	1,07985276
GSM2262259	0	0,267561	0	0,18804	0	0,462259	0,08214073	0	0,4305627	0,903364	1,014613702
GSM2262261	0	0,349498	0	0,235288	0	0,341104	0,07410948	0	0,3396767	0,949187	0,583296447
GSM2262263	0	0,39281	0	0,146168	0	0,319012	0,14200997	0	0,3354668	0,949338	0,591882732
GSM2262275	0	0,327559	0	0,134081	0,00644	0,314628	0,21729216	0	0,4602372	0,887854	0,681546513
GSM2262291	0	0,249084	0	0,093154	0	0,657762	0	0	0,5870328	0,810373	1,921946445
GSM2262294	0	0,419357	0	0,100454	0,003606	0,38901	0,08757335	0	0,4021668	0,918126	0,74836771
GSM2262296	0	0,20249	0,069457	0,190965	0,023198	0,488637	0,02525359	0	0,3708448	0,935945	1,055571606
GSM2262299	0	0,338097	0,001892	0,116058	0	0,507387	0,03656609	0	0,5090247	0,861627	1,112576985
GSM2262300	0	0,404535	0	0,163417	0,001292	0,40466	0,02609555	0	0,3689906	0,934529	0,712490666
GSM2262301	0	0,369919	0	0,209146	0	0,376301	0,04463358	0	0,3857081	0,926084	0,649842595
GSM2262308	0	0,32734	0	0,036505	0,006875	0,629281	0	0	0,59108	0,809812	1,729529757
average	0,000719	0,304918	0,015001	0,124033	0,00543	0,4844	0,06549869				

SES_CIBERSORTx_GSE85217_GP3

Sample	Bcells	TCD4	TCD8	Tgd	NK	MoMaDC	granulocytes	P-value	Correlation	RMSE		Tab
GSM2261539	0	1,08901	0,175122	0,28657	0	1,419715	0,23413709	0	0,39898443	0,919762		1,264133
GSM2261545	0	1,987295	0	0,751621	0	2,598171	0,3369664	0	0,3805862	0,929204		1,987295
GSM2261554	0	1,289376	0	0,118647	0,0802	1,748952	0,15642235	0	0,47768508	0,879683		1,289376
GSM2261555	0	1,063764	0	0,610053	0	1,274506	0,3199118	0	0,34997414	0,943313		1,063764
GSM2261559	0	0,661578	0	0,301425	0	0,361614	0,28021182	0	0,36539782	0,935674		0,661578
GSM2261560	0	1,056116	0	0,429327	0	1,498872	0,19761941	0	0,32873465	0,955518		1,056116
GSM2261561	0	1,004661	0	0,342865	0	1,28622	0,22383406	0	0,48403897	0,87573		1,004661
GSM2261565	0	0,571013	0,526348	0,287708	0	2,789945	0	0	0,45958202	0,892669		1,097361
GSM2261566	0	0,959864	0	0,613357	0	1,375789	0,29135549	0	0,45306802	0,891142		0,959864
GSM2261577	0	1,374496	0	0,455113	0	1,186518	0,16415052	0	0,41742689	0,910485		1,374496
GSM2261581	0,031488	0,644102	0,101419	0,419093	0	2,665488	0,03761417	0	0,60960762	0,792979		0,745521
GSM2261590	0	0,975453	0	0,465847	0,021628	1,53795	0,1198344	0	0,52026151	0,854703		0,975453
GSM2261592	0	1,588517	0	0,869871	0,010124	3,702582	0,14661797	0,77	0,00929035	1,121121		1,588517
GSM2261593	0	1,253429	0,246822	0,360527	0	2,356457	0	0	0,55945655	0,831396		1,500251
GSM2261594	0	1,064822	0,13146	0,505512	0	2,098937	0,29154614	0	0,47490774	0,879823		1,196282
GSM2261601	0	1,19055	0	0,277387	0	1,860393	0,12629958	0	0,40715959	0,918157		1,19055
GSM2261604	0	0,679746	0	0,516141	0	0,982949	0,28657345	0	0,41352761	0,911729		0,679746
GSM2261605	0	1,203471	0,077466	0,438606	0,060427	5,616432	0	0	0,67735575	0,737439		1,280937
GSM2261612	0	0,744987	0	0,252507	0	1,347106	0	0	0,45389853	0,892774		0,744987
GSM2261613	0	0,762687	0	0,51598	0	1,564492	0,29600658	0	0,52611488	0,850226		0,762687
GSM2261624	0	1,685513	0,565996	0,645169	0	8,189139	0	0	0,51984027	0,85775		2,251509
GSM2261625	0	1,630131	0	0,861756	0	3,268167	0,09915063	0	0,43837124	0,900766		1,630131
GSM2261628	0	0,890965	0	0,256726	0	0,758479	0,32798614	0	0,39279664	0,922276		0,890965
GSM2261635	0	0,681343	0	0,579736	0	2,769398	0	0	0,56958643	0,820977		0,681343
GSM2261636	0	0,924306	0,076842	0,461	0	2,715659	0,08891236	0	0,59571474	0,804431		1,001148
GSM2261642	0	1,072519	0	0,263832	0	1,170682	0,30114678	0	0,35704113	0,940656		1,072519
GSM2261643	0,005118	1,031362	0	0,486815	0,030595	1,378234	0,25819333	0	0,49450656	0,869424		1,031362
GSM2261645	0	1,08489	0,051722	0,516169	0,040522	1,580461	0,35586468	0	0,48864946	0,872298		1,136612
GSM2261646	0	1,070578	0	0,578789	0,016507	1,187898	0,38546658	0	0,43735217	0,899283		1,070578
GSM2261649	0	0,941072	0,056453	0,211766	0,206465	1,253466	0,14978505	0	0,42838108	0,90383		0,997525
GSM2261667	0	1,584417	0	0,547328	0	2,772659	0,14562021	0	0,56803881	0,826357		1,584417
GSM2261668	0,004562	1,058016	0	0,280293	0,001017	1,135889	0,2828162	0	0,44989886	0,89412		1,058016
GSM2261675	0	1,082979	0,086021	0,59709	0	2,276549	0,39465691	0	0,53973364	0,842362		1,169
GSM2261676	0	0,983755	0,101056	0,270646	0,007154	2,747072	0,02803677	0	0,65757748	0,760446		1,084811
GSM2261678	0	1,213492	0	0,392879	0	1,040708	0,28108029	0	0,36044769	0,937853		1,213492
GSM2261700	0	0,97871	0	0,561005	0,050105	1,834053	0,22148891	0	0,46408468	0,885468		0,97871
GSM2261701	0	1,315834	0	0,563065	0	2,810845	0,11291951	0	0,42024819	0,912164		1,315834
GSM2261711	0	0,647819	0	0,223181	0,044859	0,848426	0,07910544	0	0,43478144	0,901108		0,647819
GSM2261720	0	0,533392	0	0,510641	0	0,717317	0,33962198	0	0,47278437	0,880114		0,533392
GSM2261722	0	1,022313	0	0,576461	0	1,529064	0,29500282	0	0,46351439	0,885779		1,022313
GSM2261732	0	1,07615	0	0,336733	0	2,115528	0	0	0,54708414	0,838007		1,07615
GSM2261734	0	0,859221	0	0,472812	0,2317	1,972727	0,02532666	0	0,49080854	0,870181		0,859221
GSM2261737	0	0,949353	0	0,209283	0,093612	1,820454	0,02584809	0	0,54751469	0,837544		0,949353
GSM2261743	0	1,247029	0,156604	0,494687	0	3,754736	0,09507129	0	0,55944228	0,828578		1,403633
GSM2261745	0,001625	0,762184	0,08909	0,266947	0	1,05465	0,10765169	0	0,39473323	0,921967		0,851275
GSM2261748	0	1,173503	0,159816	0,428869	0	1,764061	0,45982134	0	0,37106853	0,933991		1,333319
GSM2261756	0	0,573824	0,396911	0,342498	0	2,290875	0,01102512	0	0,61471179	0,791424		0,970735
GSM2261757	0	1,420234	0,155861	0,494859	0	3,433091	0,11128327	0	0,62663327	0,785615		1,576094
GSM2261758	0	1,078112	0	0,242006	0,04389	2,862724	0,09820199	0	0,75714073	0,682946		1,078112
GSM2261763	0	0,972298	0	0,511528	0	1,170425	0,39490473	0	0,55528403	0,836656		0,972298
GSM2261768	0	0,908477	0,042231	0,272843	0,00682	1,525182	0,10452495	0	0,48132936	0,876928		0,950708
GSM2261793	0,007459	0,838563	0	0,508031	0	0,824162	0,39934808	0	0,40610003	0,915005		0,838563
GSM2261794	0	0,933903	0	0,372655	0	0,614276	0,16286859	0	0,4420648	0,898033		0,933903
GSM2261797	0	1,189009	0	0,552186	0	1,153013	0,36044717	0	0,30194831	0,965555		1,189009
GSM2261818	0	0,541606	0	0,319324	0,013335	0,811872	0,13591247	0	0,50399595	0,863666		0,541606
GSM2261820	0	1,022859	0	0,182108	0,075575	1,259797	0,2228559	0	0,39534652	0,921862		1,022859
GSM2261840	0,016133	0,690932	0	0,332487	0	0,436132	0,25365774	0	0,43446676	0,901239		0,690932
GSM2261841	0,029782	1,17546	0	0,53026	0	1,050991	0,41146406	0	0,39878816	0,91862		1,17546
GSM2261843	0	1,034827	0	0,217149	0	1,014349	0,1732362	0	0,43041917	0,904558		1,034827
GSM2261845	0	0,434177	0,026843	0,27122	0,042681	0,520635	0,17134757	0	0,45605627	0,889052		0,461021
GSM2261850	0	0,301806	0,100287	0,450479	0	1,333924	0,19376749	0	0,60790536	0,796139		0,402093
GSM2261855	0	0,872206	0	0,486981	0	3,6325	0	0	0,68264677	0,73521		0,872206
GSM2261863	0,13489	1,1211	0,44189	0,388586	0	2,183525	1,95219719	0	0,64544751	0,775556		1,56299
GSM2261869	0	1,018077	0,240504	0,535053	0	2,572661	0,13601738	0	0,51361373	0,85742		1,258581
GSM2261874	0	1,440613	0,15769	0,341553	0	4,387783	0,25571506	0	0,54336673	0,839818		1,598302
GSM2261878	0	0,742923	0	0,278552	0	1,006062	0,11768554	0	0,5493772	0,839652		0,742923
GSM2261881	0	0,922192	0,037825	0,347938	0	1,688479	0,11125412	0	0,32447802	0,961378		0,960018
GSM2261882	0	0,89421	0,282511	0,238776	0,203717	3,501181	0,01191539	0	0,60980183	0,792681		1,176721
GSM2261883	0	0,932731	0,027742	0,518687	0	2,168607	0	0	0,49581358	0,868219		0,960473
GSM2261885	0	0,81755	0	0,237624	0	1,249281	0,09996827	0	0,39775686	0,922261		0,81755
GSM2261896	0	0,629938	0	0,375054	0	0,840338	0,23601154	0	0,35935592	0,939034		0,629938
GSM2261900	0	0,928102	0	0,287342	0	1,717478	0,17405001	0	0,43670367	0,902406		0,928102
GSM2261919	0	0,672945	0,060465	0,376903	0	0,977718	0,26618851	0	0,51631535	0,857292		0,73341
GSM2261926	0	1,0689	0	0,361148	0	1,53831	0,19657141	0	0,50890055	0,86188		1,0689
GSM2261927	0	0,867963	0,007226	0,221513	0	1,846071	0,03965744	0	0,50776502	0,862172		0,875189
GSM2261943	0	0,890417	0,265769	0,506788	0	5,684234	0	0	0,76275925	0,659485		1,156186
GSM2261959	0	0,679737	0,011911	0,266656	0	1,086442	0,08584954	0	0,51813839	0,85615		0,691648

GSM2261978	0	0,860232	0,289009	0,16126	0	2,234134	0	0	0,62449365	0,786463		1,149241
GSM2261980	0	0,937626	0,19356	0,478557	0	1,551559	0,14812811	0	0,43647718	0,900136		1,131186
GSM2261981	0	1,1304	0	0,820619	0,013184	2,594426	0,48936948	0	0,27643171	0,98689		1,1304
GSM2262014	0	0,712834	0	0,312483	0	1,267532	0,07251776	0	0,58320387	0,817227		0,712834
GSM2262015	0,009966	1,974077	0	0,486702	0,00883	2,209286	0,14842285	0	0,46784468	0,8851		1,974077
GSM2262016	0	0,646572	0,018381	0,382597	0	0,800766	0,21356344	0	0,40320599	0,916742		0,664953
GSM2262018	0,000958	0,907828	0,0836	0,248138	0,031443	0,608286	0,27836256	0	0,33111901	0,951195		0,991428
GSM2262021	0	0,759483	0	0,519337	0	1,470739	0,23743243	0	0,34900451	0,947159		0,759483
GSM2262022	0,004588	0,677827	0	0,496476	0	0,692439	0,32882115	0	0,37482161	0,930949		0,677827
GSM2262032	0,026354	1,049207	0	0,749823	0	2,099025	0,14478334	0	0,50308616	0,863571		1,049207
GSM2262036	0	1,250748	0	0,526154	0	3,028818	0,22153871	0	0,55083412	0,834691		1,250748
GSM2262038	0	1,349483	0,115249	0,317465	0	2,097675	0,34780416	0	0,49098025	0,871788		1,464732
GSM2262039	0	0,995612	0	0,416254	0	1,833543	0,03465268	0	0,55242507	0,835233		0,995612
GSM2262048	0	0,958005	0	0,535129	0	1,102331	0,21223838	0	0,31424776	0,960783		0,958005
GSM2262056	0	1,146684	0	0,279468	0	1,215517	0,26458846	0	0,38907686	0,924869		1,146684
GSM2262058	0	0,531186	0,136686	0,231234	0	0,766053	0,13638119	0	0,57272816	0,82664		0,667872
GSM2262065	0	0,650296	0,297486	0,34974	0	1,710885	0,06907849	0	0,37927308	0,932938		0,947782
GSM2262070	0	1,212715	0	0,445818	0	1,483748	0,21014265	0	0,36694828	0,935705		1,212715
GSM2262072	0	0,820588	0	0,519357	0,046802	1,072585	0,32010493	0	0,43615936	0,899654		0,820588
GSM2262075	0	1,912695	0,260912	0,068542	0,275956	2,641432	0	0	0,29952117	0,971566		2,173607
GSM2262079	0	0,638508	0	0,189849	0,005172	0,77134	0,11944191	0	0,44747345	0,895152		0,638508
GSM2262087	0	1,005696	0	0,103098	0,31089	1,045519	0,43197169	0	0,35072877	0,942669		1,005696
GSM2262089	0	0,661705	0,125619	0,600882	0,006604	1,102598	0,57329652	0	0,49701194	0,866783		0,787323
GSM2262090	0,017908	0,823457	0	0,283226	0	0,874753	0,25134622	0	0,38147077	0,927626		0,823457
GSM2262092	0,013775	0,747488	0	0,251143	0	0,604767	0,27290348	0	0,41234104	0,912428		0,747488
GSM2262097	0	0,34942	0,379412	0,33019	0	0,63471	0,17418837	0	0,36910711	0,935101		0,728833
GSM2262101	0	0,982754	0	0,341621	0	0,78823	0,43208873	0	0,35134382	0,941912		0,982754
GSM2262108	0	1,178256	0,10314	0,526989	0	1,944452	0,14654041	0	0,35792528	0,941664		1,281397
GSM2262109	0	0,760781	0,133084	0,325636	0,022702	1,381159	0,28695128	0	0,44938375	0,893473		0,893865
GSM2262110	0	0,74768	0,200479	0,401975	0	1,886812	0,60508543	0	0,53869839	0,842347		0,948159
GSM2262112	0	0,732562	0,130965	0,585406	0,037563	1,687517	0,10193246	0	0,53906546	0,842145		0,863528
GSM2262114	0	1,190118	0,020033	0,412493	0,042187	1,858811	0,29218337	0	0,45373657	0,891517		1,210151
GSM2262118	0	0,919582	0	0,151667	0,014088	1,310743	0,07509043	0	0,45289192	0,893443		0,919582
GSM2262125	0	1,085165	0,162611	0,383747	0	0,993265	0,51981927	0	0,3519087	0,941429		1,247775
GSM2262129	0	0,850209	0	0,260403	0	1,274697	0,04781145	0	0,35578173	0,943992		0,850209
GSM2262133	0	0,955244	0	0,225952	0	1,565391	0,04526468	0	0,41784672	0,912948		0,955244
GSM2262134	0	1,235243	0	0,227137	0,017565	1,788005	0	0	0,47432971	0,881691		1,235243
GSM2262137	0	0,707932	0	0,202633	0,008676	0,542596	0,13096743	0	0,37824909	0,929465		0,707932
GSM2262140	0	0,845804	0,16133	0,375642	0,042063	1,384733	0,15425443	0	0,38922693	0,92452		1,007134
GSM2262141	0	0,852739	0	0,278834	0	0,806605	0,11492774	0	0,35143344	0,942707		0,852739
GSM2262143	0	1,403347	0	0,422307	0	1,842722	0,076432	0	0,37528144	0,932895		1,403347
GSM2262150	0	1,114516	0	0,282204	0	2,79402	0	0	0,59329324	0,806249		1,114516
GSM2262153	0	1,070543	0	0,262887	0,006356	0,966172	0,23415029	0	0,46445417	0,887104		1,070543
GSM2262155	0	1,244164	0	0,438664	0	4,82506	0	0	0,70352646	0,717157		1,244164
GSM2262157	0	1,363099	0	0,367217	0,084631	3,179864	0	0	0,64168576	0,77414		1,363099
GSM2262158	0	1,554363	0	0,143272	0,082266	1,202755	0,27378838	0	0,39058504	0,924293		1,554363
GSM2262184	0	0,87477	0,005276	0,334323	0,051276	1,344994	0,33549699	0	0,35193851	0,944067		0,880046
GSM2262192	0	1,16285	0	0,620187	0	0,978185	0,45134215	0	0,3355429	0,949626		1,16285
GSM2262195	0,172784	1,172879	0,561706	0,790017	0,050557	5,856651	0,44705126	0	0,64579358	0,767701		1,734584
GSM2262204	0	0,526835	0	0,226999	0	0,625282	0,13443858	0	0,29909467	0,967972		0,526835
GSM2262205	0	1,03365	0	0,260142	0	1,470775	0,18492508	0	0,34607545	0,948508		1,03365
GSM2262208	0	1,256061	0	0,447	0	3,246426	0,07604626	0	0,57115929	0,821183		1,256061
GSM2262209	0	0,757239	0	0,291672	0	1,14931	0,13784987	0	0,38763438	0,926527		0,757239
GSM2262229	0,008501	0,887673	0	0,270825	0,055541	1,608551	0,08225327	0	0,57076881	0,824349		0,887673
GSM2262249	0	0,86659	0	0,576202	0	0,672358	0,1991398	0	0,41412268	0,911917		0,86659
GSM2262254	0	1,115841	0	0,318524	0	1,548903	0	0	0,52354464	0,85393		1,115841
GSM2262259	0	0,512267	0	0,360018	0	0,885032	0,15726518	0	0,43056275	0,903364		0,512267
GSM2262261	0	1,006114	0	0,677334	0	0,98195	0,21334194	0	0,33967666	0,949187		1,006114
GSM2262263	0	0,754754	0	0,28085	0	0,612956	0,27286095	0	0,3354668	0,949338		0,754754
GSM2262275	0	1,189971	0	0,487097	0,023396	1,142997	0,78939011	0	0,46023722	0,887854		1,189971
GSM2262291	0	1,66199	0	0,621559	0	4,38886	0	0	0,58703278	0,810373		1,66199
GSM2262294	0	1,215057	0	0,291059	0,01045	1,127128	0,25373771	0	0,40216684	0,918126		1,215057
GSM2262296	0	0,793401	0,272149	0,748245	0,090896	1,914591	0,09894943	0	0,37084481	0,935945		1,065551
GSM2262299	0	0,618097	0,003459	0,212173	0	0,927588	0,06684887	0	0,50902471	0,861627		0,621555
GSM2262300	0	1,012165	0	0,408876	0,003233	1,012478	0,06529223	0	0,36899064	0,934529		1,012165
GSM2262301	0	1,335194	0	0,754898	0	1,358231	0,16110154	0	0,38570807	0,926084		1,335194
GSM2262308	0	1,429598	0	0,159428	0,030024	2,748268	0	0	0,59107999	0,809812		1,429598
average	0,003374	0,99284	0,059111	0,400266	0,018426	1,786536	0,19877291					

CIBERSORTx_GSE85217_GP4

Sample	Bcells	TCD4	TCD8	Tgd	NK	MoMaDC	granulocytes	P-value	Correlation	RMSE	ratio mono/T cells
GSM2261540	0	0,27726	0	0,263684	0	0,38037	0,07868532	0	0,3598455	0,940333	0,70315936
GSM2261541	0	0,179393	0	0,217109	0	0,425363	0,1781353	0	0,4104143	0,915444	1,072792035
GSM2261542	0	0,168176	0,002903	0,186812	0	0,422746	0,21936327	0	0,4269089	0,907196	1,181215112
GSM2261546	0	0,235968	0	0,255653	0	0,420279	0,08810009	0	0,3801833	0,930376	0,854885698
GSM2261556	0	0,264958	0	0,249282	0	0,349748	0,13601197	0	0,3766431	0,930994	0,680125843
GSM2261557	0	0,407516	0	0,198181	0	0,281825	0,11247922	0	0,3893759	0,923897	0,465290149
GSM2261558	0	0,304291	0	0,141177	0	0,554532	0	0	0,6588625	0,769132	1,244830504
GSM2261562	0	0,266985	0,019836	0,165446	0	0,547733	0	0	0,5350412	0,844935	1,211083682
GSM2261563	0	0,280191	0	0,184658	0	0,483421	0,05173	0	0,4372366	0,899988	1,039953419
GSM2261564	0	0,212477	0,005913	0,141141	0	0,640468	0	0	0,6266741	0,783042	1,781395926
GSM2261572	0	0,350923	0	0,184079	0	0,418305	0,04669271	0	0,3896014	0,924075	0,781875264
GSM2261573	0	0,173914	0	0,12285	0	0,703236	0	0	0,6157798	0,787883	2,369682741
GSM2261575	0	0,290185	0	0,176239	0	0,527445	0,00613139	0	0,4345432	0,902119	1,13082824
GSM2261576	0	0,259546	0	0,213155	0	0,508087	0,01921137	0	0,5068694	0,861159	1,074857504
GSM2261578	0	0,304768	0	0,225437	0	0,462326	0,00746912	0	0,4076931	0,915829	0,87197593
GSM2261579	0	0,105618	0,032041	0,094561	0,006141	0,573216	0,1884236	0	0,6514869	0,761268	2,46841736
GSM2261582	0	0,261627	0	0,182131	0	0,538031	0,01821106	0	0,5469887	0,837689	1,212442892
GSM2261583	0	0,310098	0	0,120121	0	0,541916	0,02786477	0	0,4859709	0,874145	1,259630033
GSM2261584	0	0,272433	0	0,229689	0	0,430672	0,06720567	0	0,3796134	0,929945	0,857703357
GSM2261585	0	0,296199	0	0,162922	0	0,535365	0,00551363	0	0,3937326	0,924369	1,166064948
GSM2261587	0	0,299795	0	0,256906	0	0,295501	0,1477975	0	0,388295	0,924851	0,530808104
GSM2261588	0	0,390243	0	0,127737	0	0,48202	0	0	0,4135607	0,913127	0,930575318
GSM2261595	0	0,335047	0	0,255337	0	0,280324	0,12929144	0	0,4410479	0,897451	0,4748163
GSM2261596	0	0,278191	0	0,227698	0	0,438977	0,0551342	0	0,4368451	0,899841	0,867733927
GSM2261599	0	0,340853	0	0,186719	0	0,436411	0,0360167	0	0,4039992	0,91698	0,827207495
GSM2261606	0	0,188391	0	0,128415	0	0,584332	0,09886272	0	0,4950276	0,86927	1,844450236
GSM2261607	0	0,360358	0	0,228561	0	0,344775	0,06630607	0	0,3578219	0,940093	0,585437814
GSM2261608	0	0,313662	0	0,210036	0	0,423632	0,0526694	0	0,3775117	0,930466	0,808924901
GSM2261610	0	0,276236	0	0,210787	0	0,447147	0,06582966	0	0,3560214	0,942051	0,91812317
GSM2261611	0	0,32252	0	0,230605	0	0,414327	0,03254849	0	0,3679554	0,935781	0,749066645
GSM2261616	0	0,30366	0	0,179855	0	0,510172	0,00631338	0	0,4352075	0,901486	1,055130455
GSM2261618	0	0,241079	0	0,254207	0	0,372835	0,13187843	0	0,3724885	0,933728	0,752767069
GSM2261621	0	0,31865	0	0,207004	0,00312	0,364613	0,10661438	0	0,3856225	0,925553	0,693636727
GSM2261623	0	0,198845	0	0,236529	0	0,52124	0,0433861	0	0,492003	0,869538	1,1972247
GSM2261626	0	0,337175	0	0,138798	0	0,461916	0,06211128	0	0,3354146	0,951844	0,970465924
GSM2261627	0	0,250447	0	0,255934	0	0,326559	0,16706088	0	0,449478	0,892751	0,644887453
GSM2261630	0	0,255471	0	0,152551	0	0,584466	0,00751131	0	0,4841993	0,874958	1,432436912
GSM2261631	0	0,219391	0	0,231496	0	0,526874	0,02223955	0	0,5002503	0,86473	1,168527671
GSM2261632	0	0,145286	6,19E-06	0,260498	0	0,504279	0,08993165	0	0,4272002	0,907951	1,242710235
GSM2261637	0	0,23433	0,018645	0,19879	0	0,473521	0,07471324	0	0,511416	0,858591	1,048157931
GSM2261638	0	0,294337	0	0,186767	0	0,438067	0,08082848	0	0,3925381	0,922823	0,910543979
GSM2261639	0	0,184705	0,085486	0,157643	0	0,571479	0,00068637	0	0,6538752	0,7682	1,335747659
GSM2261640	0	0,265551	0	0,1522	0	0,51917	0,06307851	0	0,5580011	0,831716	1,242772861
GSM2261651	0	0,324325	0,012009	0,158934	0	0,378054	0,12667767	0	0,3968833	0,919872	0,763332727
GSM2261652	0	0,212673	0	0,105322	0	0,682005	0	0	0,7052547	0,722208	2,144707568
GSM2261653	0	0,278143	0,020418	0,18224	0	0,445936	0,07326277	0	0,5057664	0,862532	0,927485768
GSM2261654	0	0,180159	0,038839	0,163172	0	0,617831	0	0	0,6413231	0,77349	1,61664079
GSM2261656	0	0,236711	0	0,182708	0	0,523536	0,05704427	0	0,5801119	0,817396	1,248241035
GSM2261659	0	0,353784	0	0,220095	0	0,389762	0,03635946	0	0,42779	0,904638	0,679170132
GSM2261660	0	0,322675	0	0,198358	0	0,413332	0,06563485	0	0,4048685	0,916221	0,793292863
GSM2261662	0	0,214831	0	0,181189	0,001667	0,551565	0,05074703	0	0,7135303	0,727436	1,392767132
GSM2261663	0	0,233546	0	0,214968	0	0,45646	0,09502633	0	0,4987043	0,865737	1,017715021
GSM2261664	0	0,171605	0	0,145255	0	0,61701	0,06612958	0	0,6738141	0,748159	1,947264912
GSM2261665	0	0,2403	0,000606	0,21308	0	0,492264	0,0537501	0	0,4691196	0,882519	1,084316011
GSM2261666	0	0,329028	0	0,212916	0	0,335798	0,12225843	0	0,3595558	0,938431	0,619618108
GSM2261669	0	0,290617	0	0,103056	0	0,597596	0,00873081	0	0,5511342	0,835262	1,51799931
GSM2261670	0	0,30349	0	0,180355	0	0,378002	0,13815255	0	0,4537057	0,890812	0,781245236
GSM2261671	0	0,169339	0,019772	0,213375	0	0,571845	0,02566903	0	0,5526488	0,832444	1,420781891
GSM2261672	0	0,311506	0	0,183534	0	0,382938	0,12202214	0	0,4269737	0,904653	0,773548514
GSM2261677	0	0,227688	0,04337	0,157617	0	0,539787	0,03153786	0	0,5355867	0,844321	1,259200376
GSM2261679	0	0,253968	0	0,101367	0,004124	0,639635	0,00090556	0	0,6049073	0,798832	1,800088836
GSM2261681	0	0,281557	0	0,230853	0	0,419371	0,06821942	0	0,3932821	0,922649	0,818429844
GSM2261682	0	0,256667	0,028766	0,126203	0	0,573788	0,01457583	0	0,5997248	0,804975	1,393918919
GSM2261683	0	0,166866	0	0,129997	0	0,703138	0	0	0,6962773	0,725957	2,368564555
GSM2261684	0	0,202468	0,043942	0,17553	0	0,575525	0,00253472	0	0,6445191	0,774085	1,363997169
GSM2261685	0	0,229151	0	0,183043	0	0,547167	0,0406391	0	0,5241072	0,850864	1,327450558
GSM2261686	0	0,263734	0	0,200528	0	0,489125	0,04661276	0	0,5058077	0,861965	1,053553221
GSM2261688	0	0,121389	0,069377	0,154607	0	0,601324	0,05330155	0	0,5893368	0,807998	1,741081032
GSM2261690	0	0,252455	0,001617	0,137122	0	0,608806	0	0	0,5850128	0,812855	1,556273932
GSM2261692	0	0,186729	0,002348	0,105929	0	0,704994	0	0	0,6991991	0,724041	2,389757818
GSM2261693	0	0,141646	0,10301	0,211787	0	0,504001	0,03955683	0	0,5339336	0,844375	1,104193117
GSM2261694	0	0,301802	0	0,210356	0	0,443718	0,04412456	0	0,4341332	0,901299	0,866370585
GSM2261695	0	0,243059	0,007392	0,186705	0	0,512339	0,05050431	0	0,4891955	0,871338	1,17198261
GSM2261696	0	0,275437	0,027148	0,158914	0,000535	0,422254	0,11571123	0	0,4059575	0,915687	0,914962002
GSM2261697	0	0,316049	0	0,187818	0	0,418748	0,07738469	0	0,4349284	0,900711	0,8310696
GSM2261698	0	0,313377	0	0,195287	0	0,462193	0,02914217	0	0,4241211	0,906782	0,908639926
GSM2261699	0	0,283347	0	0,193629	0,003782	0,459269	0,05997383	0	0,5430439	0,841313	0,962876793
GSM2261703	0	0,303872	0	0,237496	0	0,350315	0,10831568	0	0,4079494	0,914498	0,647091841
GSM2261704	0	0,295944	0,024253	0,199411	0	0,43796	0,04243248	0	0,4794055	0,876952	0,84286858
GSM2261705	0	0,230286	0,015264	0,149645	0	0,511258	0,09354692	0	0,6072588	0,800972	1,293684511

GSM2261714	0	0,301672	0,035222	0,140783	0	0,510911	0,01141261	0	0,5729923	0,824176	1,069574549
GSM2261721	0	0,267316	0,016096	0,158674	0	0,514586	0,04332842	0	0,5950066	0,809842	1,163995882
GSM2261723	0	0,174025	0,005028	0,083833	0	0,714783	0,02233068	0	0,6421687	0,767491	2,718979647
GSM2261724	0	0,356058	0	0,177024	0	0,387076	0,07984197	0	0,5032948	0,865261	0,726109867
GSM2261727	0	0,313414	5,67E-05	0,266491	0	0,293115	0,12692296	0	0,4267358	0,905036	0,505403421
GSM2261728	0	0,243769	0	0,170537	0	0,542603	0,04309063	0	0,5378819	0,842901	1,30966513
GSM2261730	0	0,321327	0	0,175868	0	0,376829	0,12597548	0	0,4678298	0,883597	0,757910169
GSM2261731	0	0,190721	0	0,161581	0	0,362547	0,28515187	0	0,5493692	0,835455	1,029082115
GSM2261733	0	0,186502	0,003747	0,194172	0	0,430415	0,18516346	0	0,5461101	0,837619	1,119641187
GSM2261736	0	0,258267	0	0,204536	0	0,48599	0,05120726	0	0,4419507	0,897488	1,050101639
GSM2261740	0	0,24113	0	0,197405	0,004721	0,471093	0,08565056	0	0,4830352	0,874637	1,074243673
GSM2261744	0	0,220601	0,009968	0,22128	0	0,432399	0,11575188	0	0,4934046	0,868633	0,95695415
GSM2261749	0	0,181875	0	0,256141	0	0,505798	0,05618667	0	0,5577714	0,829592	1,154747376
GSM2261751	0	0,214145	0	0,158395	0	0,560753	0,06670757	0	0,5897116	0,809881	1,505218452
GSM2261754	0	0,304529	0	0,198658	0	0,359009	0,13780381	0	0,5313333	0,849214	0,713469132
GSM2261755	0	0,286287	0	0,176444	0	0,458152	0,07911689	0	0,4600442	0,887565	0,990104403
GSM2261759	0	0,295741	0	0,085172	0	0,619087	0	0	0,5628665	0,827744	1,62526994
GSM2261761	0	0,288383	0,006525	0,220263	0	0,330609	0,15422046	0	0,3553483	0,940864	0,641745608
GSM2261764	0	0,269221	0	0,246833	0	0,349841	0,13410458	0	0,483694	0,874215	0,677914722
GSM2261767	0	0,229803	0	0,161292	0	0,51961	0,08929414	0	0,6105078	0,798304	1,328601483
GSM2261769	0	0,218222	0,002203	0,259933	0	0,377447	0,14219555	0	0,5389635	0,842323	0,785762483
GSM2261770	0	0,254398	0	0,244557	0	0,385704	0,11534072	0	0,5190406	0,854416	0,773023213
GSM2261772	0	0,306367	0	0,175055	0	0,384804	0,13377317	0	0,4421181	0,896892	0,799307388
GSM2261773	0	0,263644	0	0,16509	0	0,48847	0,08279553	0	0,5601046	0,830906	1,139332165
GSM2261774	0	0,291024	0	0,192691	0	0,407418	0,10886738	0	0,5008167	0,865473	0,842270435
GSM2261777	0	0,209682	0,009464	0,104947	0,003305	0,631221	0,04138087	0	0,6777107	0,74644	1,947656999
GSM2261778	0	0,305229	0	0,233224	0	0,331838	0,12970919	0	0,4872521	0,872683	0,616279585
GSM2261779	0	0,295451	0	0,160696	0	0,494141	0,04971245	0	0,4701671	0,882343	1,083295331
GSM2261781	0	0,330998	0,006205	0,200551	0	0,390872	0,07137498	0	0,396338	0,920384	0,726860243
GSM2261783	0	0,15623	0	0,147128	0	0,696642	0	0	0,6726576	0,744748	2,296440418
GSM2261784	0	0,370838	0	0,199636	0	0,262103	0,1674232	0	0,3690813	0,933556	0,459447576
GSM2261785	0	0,264357	0,081046	0,14034	0	0,491408	0,02284897	0	0,5882136	0,815089	1,011662562
GSM2261786	0	0,169214	0	0,150485	0	0,502611	0,17768964	0	0,591802	0,807488	1,572138507
GSM2261787	0	0,145037	0,119322	0,128177	0	0,583389	0,02407509	0	0,6046154	0,799569	1,48620415
GSM2261788	0	0,155889	0,061459	0,17901	0	0,433198	0,17044515	0	0,5807227	0,816847	1,092948517
GSM2261789	0	0,201085	0,058524	0,175028	0	0,433205	0,13215792	0	0,4291186	0,903895	0,996703531
GSM2261790	0	0,240024	0,045655	0,169905	0	0,434351	0,11006519	0	0,499551	0,865706	0,953393321
GSM2261792	0	0,309278	0	0,137388	0,002117	0,45601	0,09520729	0	0,3692487	0,935104	1,02091888
GSM2261795	0	0,162379	0	0,205601	0	0,487675	0,14434486	0	0,5952146	0,805929	1,325274365
GSM2261796	0	0,311401	0	0,199956	0	0,441604	0,04703951	0	0,4505615	0,892581	0,863592987
GSM2261799	0	0,223163	0	0,219461	0	0,478049	0,07932793	0	0,5656993	0,826299	1,080034207
GSM2261803	0	0,229349	0	0,183324	0	0,565446	0,02188076	0	0,5282532	0,848289	1,370205108
GSM2261804	0	0,155076	0,015687	0,288979	0	0,407816	0,13244202	0	0,6182628	0,791454	0,887055855
GSM2261806	0	0,264113	0,001273	0,123974	0	0,567318	0,04332206	0	0,5520775	0,834553	1,457053429
GSM2261808	0	0,34549	0	0,266431	0	0,328716	0,05936347	0	0,3963517	0,921545	0,537186914
GSM2261809	0	0,2078	0	0,083477	0,004284	0,704439	0	0	0,6142862	0,789484	2,418452008
GSM2261810	0	0,247246	0,045584	0,182168	0	0,37313	0,15187143	0	0,4766003	0,878267	0,785540731
GSM2261811	0	0,196617	0,06281	0,166699	0	0,478272	0,09560119	0	0,5296509	0,847947	1,122369889
GSM2261813	0	0,24954	0	0,124452	0	0,435271	0,19073633	0	0,3513063	0,946729	1,163851025
GSM2261814	0	0,177622	0,012343	0,196836	0	0,404957	0,20824197	0	0,5663551	0,825365	1,046939207
GSM2261819	0	0,341625	0	0,173066	0	0,410951	0,07435829	0	0,3695659	0,933831	0,798441306
GSM2261825	0	0,300007	0	0,179363	0,004848	0,492146	0,0236361	0	0,504717	0,863006	1,026651901
GSM2261827	0	0,101841	0,055497	0,106906	0	0,418874	0,31688233	0	0,615351	0,78935	1,585182994
GSM2261829	0	0,24916	0,000275	0,13954	0	0,609617	0,00140933	0	0,5873989	0,811199	1,567242438
GSM2261831	0	0,28386	0	0,107198	0	0,549298	0,05964384	0	0,5001605	0,865971	1,404646123
GSM2261833	0	0,198227	0	0,18097	0	0,60554	0,01526232	0	0,6095571	0,795336	1,596896741
GSM2261835	0	0,361367	0	0,204179	0	0,304053	0,13040102	0	0,4044414	0,916024	0,537627715
GSM2261836	0	0,302002	0,009204	0,124192	0	0,564602	0	0	0,5172621	0,855992	1,296750384
GSM2261838	0	0,216889	0	0,311885	0	0,344815	0,12640999	0	0,4601186	0,887492	0,652103097
GSM2261846	0	0,311447	0,015532	0,232138	0	0,40332	0,03756301	0	0,5405976	0,842796	0,72135113
GSM2261847	0	0,22528	0,015992	0,214877	0	0,542674	0,00117695	0,02	0,2070456	1,023101	1,189686162
GSM2261848	0	0,20118	0	0,179209	0	0,583538	0,03607348	0	0,5425085	0,839174	1,534056041
GSM2261852	0	0,184791	0,018215	0,183617	0	0,51183	0,1015469	0	0,3172743	0,966598	1,323846309
GSM2261854	0	0,278225	0	0,165609	0	0,556166	0	0	0,5745233	0,821133	1,25309692
GSM2261857	0	0,370935	0	0,122477	0	0,46787	0,03871827	0	0,4356592	0,901352	0,948233129
GSM2261864	0	0,219817	0	0,204711	0	0,441606	0,13386521	0	0,4304391	0,90366	1,0402269
GSM2261865	0	0,238282	0,013444	0,138114	0	0,551444	0,05871661	0	0,5624251	0,828012	1,414538552
GSM2261868	0	0,210119	0	0,130907	0	0,658975	0	0	0,6524915	0,763674	1,932333252
GSM2261870	0	0,274846	0	0,132628	0	0,498253	0,09427245	0	0,4699791	0,882658	1,222785557
GSM2261871	0	0,216601	0	0,234591	0	0,481257	0,06755103	0	0,4814587	0,875448	1,066632723
GSM2261872	0	0,234539	0,022461	0,179731	0	0,465967	0,09730122	0	0,5136798	0,857482	1,066939692
GSM2261873	0	0,277012	0	0,114579	0	0,601974	0,00643431	0	0,48286	0,87638	1,537251508
GSM2261879	0	0,335902	0	0,124842	0	0,401092	0,13816499	0	0,4673105	0,884427	0,870532407
GSM2261880	0,000733	0,308809	0	0,224267	0	0,436333	0,02985769	0	0,330597	0,954901	0,818518766
GSM2261884	0	0,265331	0	0,081563	0	0,653105	0	0	0,530529	0,847949	1,882719257
GSM2261886	0	0,229826	0,034736	0,164441	0	0,4921	0,0788975	0	0,5152799	0,856498	1,147077115
GSM2261887	0	0,293228	0	0,144099	0	0,500783	0,06188979	0	0,4315346	0,903496	1,145100208
GSM2261892	0	0,18704	0	0,216383	0	0,573135	0,02344193	0	0,5826326	0,813359	1,420678511
GSM2261895	0	0,218929	0	0,271	0	0,407529	0,10254157	0	0,5276288	0,848669	0,831811835
GSM2261897	0	0,379586	0	0,166597	0	0,395377	0,0584395	0	0,4217677	0,907771	0,72389091
GSM2261898	0	0,253478	0	0,164965	0	0,512028	0,06952964	0	0,5271453	0,849692	1,223651575
GSM2261899	0,001232	0,344772	0	0,219666	0	0,314694	0,11963561	0	0,4587644	0,8882	0,557535101
GSM2261901	0	0,339249	0,04465	0,045198	0	0,570903	0	0	0,7196436	0,728777	1,330475407

GSM2261902	0	0,206713	0,066053	0,167296	0	0,559937	0	0	0,6840242	0,74898	1,272402482
GSM2261903	0	0,173962	0,000761	0,202546	0	0,611681	0,01104989	0	0,5790735	0,814911	1,621337003
GSM2261904	0	0,240623	0,043109	0,151338	0	0,489191	0,07573998	0	0,4815911	0,87569	1,124397674
GSM2261906	0	0,313132	0	0,181918	0	0,422677	0,0822737	0	0,4696933	0,882469	0,853806049
GSM2261908	0	0,276194	0,027918	0,200354	0	0,344048	0,15148603	0	0,441654	0,896779	0,68200369
GSM2261909	0	0,304838	0	0,205111	0	0,408679	0,08137222	0	0,4270284	0,904727	0,801412038
GSM2261910	0	0,151974	0,053279	0,198002	0	0,42219	0,17455519	0	0,5534735	0,833148	1,046955353
GSM2261912	0	0,252507	0,007014	0,209101	0	0,46616	0,06521757	0	0,4619535	0,886329	0,994746731
GSM2261913	0	0,293797	0	0,190114	0	0,376704	0,13938619	0	0,4884523	0,872292	0,778457578
GSM2261916	0	0,181285	0,000375	0,202817	0	0,557345	0,05817896	0	0,5911319	0,808104	1,449619036
GSM2261917	0	0,282196	0	0,203846	0	0,435833	0,07812497	0	0,4566155	0,889185	0,896698387
GSM2261918	0	0,202335	0	0,21476	0	0,541301	0,04160388	0	0,625186	0,786979	1,297789677
GSM2261920	0	0,281554	0	0,120428	0	0,553112	0,04490547	0	0,5645381	0,827573	1,37596095
GSM2261921	0	0,2255	0	0,228958	0	0,46707	0,0784718	0	0,4701074	0,88181	1,02775157
GSM2261922	0	0,247315	0	0,257671	0	0,435013	0,06000159	0,27	0,0982178	1,069497	0,861437479
GSM2261923	0	0,265132	0	0,174537	0	0,511478	0,04885189	0	0,5005883	0,865071	1,163323281
GSM2261924	0	0,183484	0,117029	0,108659	0	0,590828	0	0	0,5948551	0,806592	1,443957262
GSM2261925	0	0,270512	0,003901	0,168633	0	0,472627	0,08432709	0	0,530595	0,848364	1,066769306
GSM2261928	0	0,141847	0	0,21974	0	0,391981	0,24643124	0	0,6368631	0,778209	1,084056832
GSM2261935	0	0,239889	0,034884	0,121054	0	0,593027	0,01114613	0	0,5993144	0,80421	1,498198712
GSM2261939	0	0,182263	0	0,142156	0	0,543284	0,13229675	0	0,6501716	0,768159	1,674633844
GSM2261945	0	0,250856	0,019905	0,15451	0	0,523185	0,05154414	0	0,5158019	0,856299	1,230236977
GSM2261951	0	0,275446	0	0,154291	0	0,458224	0,1120395	0	0,4501185	0,89308	1,066290603
GSM2261953	0	0,362946	0	0,168711	0	0,412508	0,05583588	0	0,4583452	0,888939	0,775891282
GSM2261955	0	0,274932	0	0,189142	0	0,535926	0	0	0,5246564	0,851012	1,154830595
GSM2261957	0	0,315784	0	0,127422	0	0,458136	0,09865756	0	0,4695108	0,883046	1,033684286
GSM2261962	0	0,18142	0,026745	0,140901	0	0,574624	0,07631024	0	0,535473	0,843627	1,646177731
GSM2261963	0	0,301021	0	0,243992	0	0,353928	0,10105815	0	0,4444857	0,895461	0,649393856
GSM2261964	0	0,177865	0,047281	0,211452	0	0,545353	0,01804893	0	0,5097905	0,858975	1,249096497
GSM2261966	0	0,318703	0	0,223412	0	0,324928	0,13295658	0	0,3826069	0,927091	0,599370686
GSM2261967	0	0,198045	0	0,243653	0	0,48747	0,07083205	0	0,4510051	0,892944	1,103627595
GSM2261976	0	0,363491	0	0,131817	0,007089	0,416927	0,08067701	0	0,4969954	0,868953	0,841754136
GSM2261977	0	0,243446	0	0,179576	0	0,53088	0,04609814	0	0,5332475	0,845713	1,254968028
GSM2261985	0	0,172276	0	0,120351	0	0,707373	0	0	0,725332	0,702014	2,417324409
GSM2261988	0	0,262288	0	0,235319	0	0,316808	0,18558439	0	0,4155771	0,910635	0,636663825
GSM2261989	0	0,300972	0	0,225205	0	0,413035	0,06078761	0	0,3942484	0,92196	0,784973139
GSM2261991	0	0,278432	0	0,194242	0	0,389447	0,13787967	0	0,397588	0,919879	0,823924019
GSM2261992	0	0,351217	0	0,186698	0	0,37005	0,09203569	0	0,3866439	0,924998	0,687933952
GSM2261996	0	0,175411	0,108702	0,122063	0	0,415151	0,17867282	0	0,4878526	0,871913	1,022097181
GSM2261998	0	0,315002	0	0,277258	0	0,31673	0,0910109	0,32	0,0869392	1,071926	0,534782023
GSM2262001	0	0,342952	0	0,177993	0	0,405033	0,07402206	0	0,4645449	0,885459	0,777498195
GSM2262003	0	0,241119	0,026029	0,143556	0	0,325399	0,26389811	0	0,5558548	0,833846	0,792296761
GSM2262004	0	0,313401	0	0,203941	0	0,318823	0,16383543	0	0,421385	0,907345	0,616271286
GSM2262007	0	0,223737	0,001176	0,165976	0	0,583845	0,0252664	0	0,5794066	0,816283	1,493637987
GSM2262013	0	0,257274	0,026118	0,17963	0	0,481759	0,05521871	0	0,4700908	0,881977	1,040466978
GSM2262017	0	0,229177	0,009509	0,153575	0	0,528855	0,07888374	0	0,4450849	0,896743	1,348221313
GSM2262020	0	0,267192	0	0,220336	0	0,380254	0,13221846	0	0,4629899	0,885545	0,779963742
GSM2262023	0	0,320795	0	0,219457	0	0,347467	0,11228159	0	0,3747954	0,931054	0,643158671
GSM2262025	0	0,240226	0	0,165749	0	0,543949	0,05007601	0	0,6199034	0,792073	1,339859426
GSM2262026	0	0,27309	0	0,258475	0	0,302606	0,16582919	0	0,4048642	0,916389	0,569273969
GSM2262028	0	0,306654	0	0,200368	0	0,388925	0,10405368	0	0,451529	0,891864	0,767078671
GSM2262030	0	0,317877	0	0,214207	0	0,409809	0,05810746	0	0,4604197	0,887231	0,77019639
GSM2262031	0	0,333899	0	0,257743	0	0,358173	0,05018493	0	0,3336406	0,953516	0,605387091
GSM2262033	0	0,27024	0	0,203311	0	0,505621	0,0208284	0	0,4415626	0,897947	1,067723313
GSM2262034	0	0,346484	0,002526	0,201689	0	0,391391	0,05790955	0	0,3632424	0,937107	0,710717335
GSM2262035	0	0,261697	0	0,232849	0	0,412468	0,09298627	0	0,47578	0,878573	0,834032861
GSM2262041	0	0,238937	0	0,287957	0	0,351702	0,12140382	0	0,5056593	0,861458	0,667499765
GSM2262042	0	0,193828	0,03513	0,100321	0	0,60081	0,06991031	0	0,5178231	0,855071	1,824617006
GSM2262044	0	0,323322	0,017346	0,19887	0	0,38749	0,07297138	0	0,4761532	0,878985	0,718189061
GSM2262046	0	0,336333	0	0,227409	0	0,349057	0,08720151	0	0,3509421	0,943222	0,619178161
GSM2262049	0	0,151985	0,00728	0,048479	0	0,792256	0	0	0,6882746	0,726453	3,813608302
GSM2262051	0	0,334595	0	0,137475	0,008598	0,372266	0,14706646	0	0,3803882	0,928085	0,788583381
GSM2262053	0	0,304028	0	0,261645	0	0,25021	0,18411723	0	0,3749055	0,932024	0,442322194
GSM2262059	0	0,228082	0,002178	0,184605	0	0,570874	0,01426058	0	0,5463267	0,837194	1,376046229
GSM2262060	0	0,315082	0	0,246558	0	0,372215	0,06614419	0	0,3567773	0,941196	0,662728261
GSM2262061	0	0,240772	0	0,268121	0	0,404186	0,08692069	0	0,4351719	0,900894	0,794243416
GSM2262063	0	0,37526	0	0,172292	0	0,427638	0,02480942	0	0,3871657	0,925586	0,781000493
GSM2262064	0	0,218544	0,00828	0,202709	0	0,517893	0,05257467	0	0,4257767	0,90712	1,205715363
GSM2262067	0	0,266352	0	0,127049	0,003055	0,547507	0,05603643	0	0,5007263	0,865277	1,391729274
GSM2262068	0	0,287788	0	0,142126	0	0,485303	0,08478383	0	0,4565132	0,889888	1,128838043
GSM2262077	0	0,351772	0	0,165433	0,004254	0,354266	0,12427471	0	0,3212869	0,95636	0,68496321
GSM2262078	0	0,319029	0	0,209013	0	0,328971	0,14298711	0	0,4866052	0,87344	0,623000936
GSM2262080	0	0,30995	0,00215	0,202254	0	0,396443	0,08920341	0	0,3586326	0,939376	0,770758496
GSM2262082	0	0,270924	0,01189	0,213779	0	0,458149	0,04525803	0	0,4364645	0,900131	0,922586343
GSM2262083	0	0,197475	0	0,223247	0	0,478689	0,10058817	0	0,5079103	0,860149	1,137778929
GSM2262086	0	0,263008	0	0,1399	0,002874	0,538357	0,05586115	0	0,5383725	0,843068	1,336178039
GSM2262091	0	0,242911	0	0,131981	0	0,625108	0	0	0,4982298	0,867255	1,667436866
GSM2262094	0	0,223532	0,063702	0,141522	0	0,517812	0,05343148	0	0,5159647	0,856076	1,207707597
GSM2262098	0	0,344739	0	0,121158	0	0,505943	0,02816049	0	0,4216614	0,909028	1,085956186
GSM2262102	0	0,198693	0,025661	0,125544	0	0,650102	0	0	0,5750324	0,817855	1,85797951
GSM2262103	0	0,254013	0	0,211219	0	0,353198	0,18156987	0	0,4592268	0,887574	0,759187862
GSM2262104	0	0,179977	0,013312	0,132121	0	0,519735	0,15485495	0	0,5562343	0,830695	1,597169159
GSM2262105	0	0,280032	0	0,145006	0	0,501241	0,07372088	0	0,5068106	0,861925	1,179284109

GSM2262107	0	0,259284	0,063639	0,191599	0	0,374069	0,11140979	0	0,5167811	0,856551	0,727022327
GSM2262116	0,014739	0,104479	0,02167	0,105623	0,006221	0,670325	0,07694189	0	0,6550247	0,757527	2,892174228
GSM2262117	0	0,295512	0	0,177497	0	0,409496	0,11749505	0	0,47924	0,877283	0,865725743
GSM2262119	0	0,262642	0,012564	0,081865	0	0,642929	0	0	0,661068	0,760206	1,800560069
GSM2262120	0	0,332788	0,000884	0,22195	0	0,36527	0,07910752	0	0,470707	0,881799	0,657407243
GSM2262122	0	0,392371	0	0,141939	0	0,336357	0,12933291	0	0,4097048	0,91372	0,629515931
GSM2262123	0	0,270588	0,003018	0,179856	0	0,479665	0,06687356	0	0,5400997	0,84275	1,057786136
GSM2262124	0	0,341541	0	0,109762	0	0,548697	0	0	0,4419429	0,898983	1,215804641
GSM2262126	0	0,125585	0,115379	0,128797	0	0,63024	0	0	0,6633705	0,756865	1,704454424
GSM2262127	0	0,310225	0	0,194676	0	0,474822	0,02027744	0	0,4311099	0,903232	0,94042707
GSM2262131	0	0,280924	0	0,211505	0	0,414477	0,0930941	0	0,4406624	0,897578	0,841699349
GSM2262138	0	0,31151	0	0,160743	0	0,500581	0,02716564	0	0,3899282	0,925325	1,059984459
GSM2262144	0	0,342588	0	0,16173	0	0,455865	0,0398166	0	0,4193203	0,909286	0,903924068
GSM2262146	0	0,284539	0	0,11254	2,18E-05	0,589109	0,01379047	0	0,5212913	0,853405	1,483605277
GSM2262148	0,003911	0,268477	0	0,168873	0,01396	0,495678	0,04910115	0	0,5410698	0,841946	1,133366471
GSM2262149	0	0,175427	0,060993	0,132475	0	0,542669	0,08843644	0	0,5743293	0,819805	1,471065735
GSM2262152	0	0,309629	0	0,216658	0	0,418418	0,05529525	0	0,4589056	0,888003	0,795036533
GSM2262159	0	0,281339	0	0,232556	0	0,43769	0,04841515	0	0,3755406	0,932241	0,851709625
GSM2262161	0	0,240112	0	0,202253	0,001941	0,531867	0,02382742	0	0,5607044	0,828933	1,202328108
GSM2262162	0	0,198492	0,008497	0,201865	0	0,541374	0,04977116	0	0,5826182	0,814449	1,324123795
GSM2262163	0	0,278046	0	0,228185	0	0,475502	0,01826578	0	0,6166922	0,796243	0,939298028
GSM2262164	0	0,291692	0	0,199213	0	0,503666	0,00542933	0	0,4566785	0,889652	1,025993571
GSM2262166	0	0,302299	0,027281	0,207658	0	0,399636	0,06312583	0	0,50214	0,864665	0,743872332
GSM2262168	0	0,179467	0	0,150198	0	0,655617	0,01471768	0	0,5470404	0,836218	1,988736695
GSM2262173	0	0,357411	0	0,197336	0,002476	0,397275	0,04550174	0	0,3596405	0,938995	0,716135523
GSM2262175	0	0,254797	0,013124	0,089817	0	0,642262	0	0	0,5932091	0,806795	1,795344723
GSM2262176	0	0,237851	0	0,11601	0	0,519777	0,12636216	0	0,4821169	0,876197	1,468870857
GSM2262177	0	0,251294	0	0,146468	0	0,543568	0,05867016	0	0,5015412	0,864605	1,366566257
GSM2262179	0	0,254796	0	0,254001	0	0,428747	0,06245608	0	0,5280591	0,848883	0,842666661
GSM2262180	0	0,27167	0,000111	0,19885	0	0,465969	0,0634008	0	0,503316	0,863555	0,990095338
GSM2262183	0	0,44675	0	0,174825	0	0,292713	0,08571219	0	0,3437489	0,946435	0,470921899
GSM2262185	0	0,36632	0	0,184121	0	0,417268	0,03229062	0	0,384678	0,926699	0,758061385
GSM2262186	0	0,282196	0	0,157968	0	0,511206	0,04863053	0	0,4440619	0,89675	1,161399592
GSM2262187	0	0,244197	0,059318	0,165178	0	0,531307	0	0	0,5882715	0,812983	1,133594522
GSM2262191	0	0,328264	0	0,195279	0	0,415142	0,06131527	0	0,3040853	0,966383	0,792946408
GSM2262193	0	0,219466	0,012826	0,229056	0	0,523451	0,01520158	0	0,5098974	0,859023	1,134611434
GSM2262194	0	0,280284	0	0,180221	0	0,452798	0,08669708	0	0,4476145	0,894178	0,983265607
GSM2262196	0	0,284524	0,112861	0,089407	0	0,488209	0,02499976	0	0,5938764	0,812888	1,002912417
GSM2262200	0	0,243691	0	0,248068	0	0,508241	0	0	0,4214932	0,909665	1,033516037
GSM2262201	0	0,259992	0	0,20125	0	0,519123	0,01963491	0	0,4604516	0,887635	1,12548993
GSM2262203	0	0,24266	0,022493	0,170567	0	0,476378	0,08790162	0	0,4199396	0,909243	1,093311995
GSM2262211	0	0,290829	0	0,150412	0	0,55876	0	0	0,4619132	0,887522	1,266338691
GSM2262213	0	0,367623	0	0,263978	0	0,279253	0,08914544	0	0,3136758	0,963696	0,44213394
GSM2262215	0	0,296621	0	0,209504	0	0,482792	0,0110836	0	0,3859745	0,927401	0,953899535
GSM2262216	0	0,261102	0	0,195423	0	0,510223	0,03325247	0	0,401476	0,919817	1,117625849
GSM2262219	0	0,142752	0	0,145513	0	0,711734	0	0	0,5214516	0,854933	2,469023144
GSM2262221	0	0,163941	0,009104	0,102634	0	0,59333	0,13099073	0	0,6937203	0,732027	2,152251404
GSM2262224	0	0,207713	0,006406	0,12171	0	0,606595	0,057577	0	0,4893891	0,872758	1,806266082
GSM2262227	0	0,090243	0,003911	0,183794	0,017903	0,407568	0,29658096	0	0,6385374	0,77279	1,466346356
GSM2262228	0	0,267776	0	0,129438	0	0,546245	0,05654149	0	0,5490932	0,836705	1,375192035
GSM2262231	0	0,106445	0,010019	0,073086	0	0,596948	0,21350129	0	0,6988897	0,720178	3,149286783
GSM2262235	0	0,272759	0,001144	0,150037	0	0,5188	0,05726129	0	0,4779209	0,878122	1,22375947
GSM2262237	0	0,188073	0,015726	0,061962	0	0,734238	0	0	0,6451119	0,765157	2,762772119
GSM2262238	0	0,209969	0	0,260129	0	0,362156	0,16774561	0	0,40348	0,918029	0,770385054
GSM2262239	0	0,271283	0	0,210187	0	0,467746	0,05078312	0	0,4315011	0,90292	0,971494526
GSM2262241	0	0,173522	0	0,177886	0	0,502063	0,14652939	0	0,4644185	0,886191	1,428716348
GSM2262242	0	0,363293	0	0,208263	0	0,371051	0,05739338	0	0,3867926	0,925352	0,649193744
GSM2262243	0	0,298722	0	0,224848	0	0,311076	0,16535399	0	0,4067348	0,914912	0,59414388
GSM2262246	0	0,281566	0	0,077437	0,007718	0,633279	0	0	0,5904901	0,809188	1,763991084
GSM2262251	0	0,306048	0	0,211315	0	0,356418	0,12621937	0	0,3741888	0,931335	0,6889122
GSM2262253	0	0,388774	0	0,23795	0	0,286666	0,08660929	0	0,3323123	0,953218	0,457404397
GSM2262256	0	0,364277	0	0,214575	0	0,356961	0,06418744	0	0,3754936	0,931012	0,616669918
GSM2262258	0	0,243027	0	0,194719	0	0,387728	0,17452612	0	0,4600763	0,88724	0,885735536
GSM2262264	0	0,349256	0	0,23245	0,008692	0,39632	0,01328201	0	0,4431298	0,896665	0,681306814
GSM2262265	0	0,335482	0	0,178499	0	0,352916	0,13310365	0	0,4040753	0,916165	0,686632906
GSM2262266	0	0,277653	0	0,247161	0	0,365363	0,10982275	0	0,4296568	0,903319	0,696177223
GSM2262268	0	0,301624	0	0,214355	0	0,351414	0,1326064	0	0,4538456	0,890527	0,681063186
GSM2262270	0	0,350599	0	0,19801	0	0,384294	0,06709688	0	0,3166383	0,959711	0,700488638
GSM2262274	0	0,307468	0	0,162862	0	0,52967	0	0	0,4404346	0,898985	1,1261663
GSM2262276	0	0,277476	0	0,182542	0	0,448489	0,09149347	0	0,4626012	0,88609	0,974937811
GSM2262282	0	0,274133	0	0,20497	0	0,520898	0	0	0,4254132	0,907108	1,087236982
GSM2262290	0	0,220096	0	0,174806	0	0,52084	0,08425753	0	0,5509595	0,834812	1,318910387
GSM2262293	0	0,224589	0	0,347374	0	0,340788	0,08724883	0	0,4097772	0,917322	0,595821362
GSM2262297	0	0,283189	0	0,174355	0	0,537242	0,00521447	0	0,4349881	0,902066	1,174189695
GSM2262303	0	0,184638	0,021264	0,186729	0	0,501315	0,10605433	0	0,5506549	0,834665	1,276808565
GSM2262305	0	0,28583	0	0,223337	0	0,490833	0	0	0,4668311	0,883927	0,963991126
GSM2262307	0	0,320683	0	0,19952	0	0,450515	0,0292826	0	0,5112922	0,859671	0,866036351
GSM2262311	0	0,328465	0	0,246541	0,014404	0,358989	0,05160085	0	0,3985158	0,919988	0,624321341
GSM2262312	0	0,359627	0	0,217841	0	0,361882	0,06064947	0	0,4177022	0,909678	0,626670209
GSM2262313	0	0,291208	0	0,21012	0	0,412632	0,08604071	0	0,4714277	0,8812	0,823077375
**average**	**6,32365E-[illegible]**	**0,262823**	**0,008781**	**0,182259**	**0,000423**	**0,473163**	**0,07248865**				

SES_CIBERSORTx_GSE85217_GP4

Sample	Bcells	TCD4	TCD8	Tgd	NK	MoMaDC	granulocytes	P-value	Correlation	RMSE		Tab
GSM2261540	0	1,048256	0	0,99693	0	1,438091	0,29749091	0	0,3598455	0,940333		1,048256
GSM2261541	0	0,68413	0	0,827963	0	1,622162	0,67933499	0	0,4104143	0,915444		0,68413
GSM2261542	0	0,755829	0,013047	0,839584	0	1,899937	0,98587899	0	0,4269089	0,907196		0,768876
GSM2261546	0	0,817528	0	0,88573	0	1,456091	0,30522966	0	0,3801833	0,930376		0,817528
GSM2261556	0	0,629614	0	0,592365	0	0,831099	0,32320261	0	0,3766431	0,930994		0,629614
GSM2261557	0	1,220233	0	0,593416	0	0,843873	0,33679887	0	0,3893759	0,923897		1,220233
GSM2261558	0	2,890453	0	1,341038	0	5,267489	0	0	0,6588625	0,769132		2,890453
GSM2261562	0	1,483726	0,110233	0,919442	0	3,043939	0	0	0,5350412	0,844935		1,593959
GSM2261563	0	1,286151	0	0,847632	0	2,219035	0,23745486	0	0,4372366	0,899988		1,286151
GSM2261564	0	0,923103	0,02569	0,613186	0	2,782502	0	0	0,6266741	0,783042		0,948793
GSM2261572	0	1,272266	0	0,667374	0	1,516556	0,16928342	0	0,3896014	0,924075		1,272266
GSM2261573	0	1,221358	0	0,862742	0	4,938655	0	0	0,6157798	0,787883		1,221358
GSM2261575	0	1,088281	0	0,66095	0	1,97808	0,02299456	0	0,4345432	0,902119		1,088281
GSM2261576	0	1,371158	0	1,126079	0	2,684175	0,10149182	0	0,5068694	0,861159		1,371158
GSM2261578	0	1,314017	0	0,971978	0	1,993332	0,03220333	0	0,4076931	0,915829		1,314017
GSM2261579	0	0,928242	0,281596	0,831071	0,053972	5,037814	1,6559968	0	0,6514869	0,761268		1,209837
GSM2261582	0	0,908717	0	0,632604	0	1,868764	0,06325316	0	0,5469887	0,837689		0,908717
GSM2261583	0	1,284178	0	0,497447	0	2,244189	0,11539378	0	0,4859709	0,874145		1,284178
GSM2261584	0	0,916461	0	0,77267	0	1,448774	0,22607879	0	0,3796134	0,929945		0,916461
GSM2261585	0	1,211025	0	0,666116	0	2,188869	0,02254274	0	0,3937326	0,924369		1,211025
GSM2261587	0	0,965575	0	0,827437	0	0,951745	0,47602321	0	0,388295	0,924851		0,965575
GSM2261588	0	1,39701	0	0,457278	0	1,725554	0	0	0,4135607	0,913127		1,39701
GSM2261595	0	0,888439	0	0,677073	0	0,74333	0,34283977	0	0,4410479	0,897451		0,888439
GSM2261596	0	1,2184	0	0,997252	0	1,922597	0,24147242	0	0,4368451	0,899841		1,2184
GSM2261599	0	1,577004	0	0,863878	0	2,019116	0,16663607	0	0,4039992	0,91698		1,577004
GSM2261606	0	1,050527	0	0,716082	0	3,258423	0,55129042	0	0,4950276	0,86927		1,050527
GSM2261607	0	1,023169	0	0,648957	0	0,978926	0,18826391	0	0,3578219	0,940093		1,023169
GSM2261608	0	1,11907	0	0,749357	0	1,511417	0,18791156	0	0,3775117	0,930466		1,11907
GSM2261610	0	0,825345	0	0,629794	0	1,335997	0,19668739	0	0,3560214	0,942051		0,825345
GSM2261611	0	0,943665	0	0,674728	0	1,212284	0,09523397	0	0,3679554	0,935781		0,943665
GSM2261616	0	1,068174	0	0,63267	0	1,794612	0,02220834	0	0,4352075	0,901486		1,068174
GSM2261618	0	0,733854	0	0,773816	0	1,134924	0,4014429	0	0,3724885	0,933728		0,733854
GSM2261621	0	1,080545	0	0,701953	0,010578	1,236406	0,36153087	0	0,3856225	0,925553		1,080545
GSM2261623	0	0,924789	0	1,100049	0	2,424186	0,20178022	0	0,492003	0,869538		0,924789
GSM2261626	0	1,118992	0	0,460634	0	1,532973	0,20613058	0	0,3354146	0,951844		1,118992
GSM2261627	0	1,176643	0	1,202424	0	1,53423	0,7848818	0	0,449478	0,892751		1,176643
GSM2261630	0	1,98556	0	1,185647	0	4,542554	0,05837899	0	0,4841993	0,874958		1,98556
GSM2261631	0	1,119775	0	1,181555	0	2,689168	0,11351087	0	0,5002503	0,86473		1,119775
GSM2261632	0	0,643897	2,74E-05	1,15451	0	2,234932	0,39857146	0	0,4272002	0,907951		0,643924
GSM2261637	0	0,65133	0,051823	0,552545	0	1,316171	0,20766824	0	0,511416	0,858591		0,703153
GSM2261638	0	1,553379	0	0,985672	0	2,311917	0,42657581	0	0,3925381	0,922823		1,553379
GSM2261639	0	0,84341	0,390353	0,719839	0	2,60952	0,00313412	0	0,6538752	0,7682		1,233763
GSM2261640	0	0,744217	0	0,426546	0	1,454992	0,17677976	0	0,5580011	0,831716		0,744217
GSM2261651	0	0,654346	0,02423	0,32066	0	0,762749	0,2555805	0	0,3968833	0,919872		0,678576
GSM2261652	0	1,622227	0	0,803377	0	5,202212	0	0	0,7052547	0,722208		1,622227
GSM2261653	0	0,988988	0,072601	0,647989	0	1,58561	0,26049951	0	0,5057664	0,862532		1,061589
GSM2261654	0	1,383109	0,29817	1,252694	0	4,74318	0	0	0,6413231	0,77349		1,681279
GSM2261656	0	1,404887	0	1,084376	0	3,107201	0,33855909	0	0,5801119	0,817396		1,404887
GSM2261659	0	1,436977	0	0,893967	0	1,583108	0,14768249	0	0,42779	0,904638		1,436977
GSM2261660	0	0,998833	0	0,614013	0	1,279459	0,20317114	0	0,4048685	0,916221		0,998833
GSM2261662	0	1,085295	0	0,915342	0,008422	2,786422	0,25636623	0	0,7135303	0,727436		1,085295
GSM2261663	0	0,785171	0	0,722711	0	1,534594	0,31947367	0	0,4987043	0,865737		0,785171
GSM2261664	0	1,215712	0	1,029042	0	4,37113	0,46848641	0	0,6738141	0,748159		1,215712
GSM2261665	0	0,672991	0,001696	0,596758	0	1,378647	0,15053387	0	0,4691196	0,882519		0,674687
GSM2261666	0	1,035429	0	0,670034	0	1,056736	0,38473979	0	0,3595558	0,938431		1,035429
GSM2261669	0	1,249035	0	0,442922	0	2,568389	0,03752389	0	0,5511342	0,835262		1,249035
GSM2261670	0	0,875152	0	0,520077	0	1,090016	0,39838009	0	0,4537057	0,890812		0,875152
GSM2261671	0	0,665064	0,077654	0,838009	0	2,245868	0,10081272	0	0,5526488	0,832444		0,742718
GSM2261672	0	1,49065	0	0,878266	0	1,832471	0,58391245	0	0,4269737	0,904653		1,49065
GSM2261677	0	0,79135	0,150736	0,547813	0	1,876082	0,10961277	0	0,5355867	0,844321		0,942087
GSM2261679	0	1,256445	0	0,501491	0,020403	3,164441	0,00448003	0	0,6049073	0,798832		1,256445
GSM2261681	0	0,848171	0	0,695429	0	1,263328	0,20550657	0	0,3932821	0,922649		0,848171
GSM2261682	0	0,990756	0,11104	0,487155	0	2,214868	0,05626392	0	0,5997248	0,804975		1,101795
GSM2261683	0	1,360553	0	1,059936	0	5,733083	0	0	0,6962773	0,725957		1,360553
GSM2261684	0	1,245356	0,270279	1,079664	0	3,539981	0,01559074	0	0,6445191	0,774085		1,515636
GSM2261685	0	0,826423	0	0,660135	0	1,973333	0,14656305	0	0,5241072	0,850864		0,826423
GSM2261686	0	1,382597	0	1,051248	0	2,564184	0,24436231	0	0,5058077	0,861965		1,382597
GSM2261688	0	0,610191	0,348741	0,777167	0	3,022689	0,26793199	0	0,5893368	0,807998		0,958932
GSM2261690	0	0,933381	0,005978	0,506971	0	2,250884	0	0	0,5850128	0,812855		0,939358
GSM2261692	0	1,462673	0,018389	0,829756	0	5,522296	0	0	0,6991991	0,724041		1,481062
GSM2261693	0	0,704258	0,512164	1,052997	0	2,505876	0,19667536	0	0,5339336	0,844375		1,216421
GSM2261694	0	0,711163	0	0,495681	0	1,045574	0,10397477	0	0,4341332	0,901299		0,711163
GSM2261695	0	0,637272	0,01938	0,489519	0	1,343293	0,13241631	0	0,4891955	0,871338		0,656652
GSM2261696	0	0,722042	0,071168	0,416583	0,001403	1,106915	0,30333034	0	0,4059575	0,915687		0,79321
GSM2261697	0	0,795732	0	0,47288	0	1,054305	0,1948355	0	0,4349284	0,900711		0,795732
GSM2261698	0	0,913594	0	0,569324	0	1,347439	0,08495863	0	0,4241211	0,906782		0,913594
GSM2261699	0	0,956357	0	0,653539	0,012764	1,550131	0,2024246	0	0,5430439	0,841313		0,956357
GSM2261703	0	0,954428	0	0,745948	0	1,100299	0,34020677	0	0,4079494	0,914498		0,954428
GSM2261704	0	1,164233	0,09541	0,784476	0	1,722923	0,16692809	0	0,4794055	0,876952		1,259643
GSM2261705	0	0,938567	0,062212	0,6099	0	2,08371	0,38126488	0	0,6072588	0,800972		1,000779

GSM2261714	0	0,956597	0,111689	0,44642	0	1,62009	0,03618921	0	0,5729923	0,824176		1,068286
GSM2261721	0	1,188984	0,071592	0,705763	0	2,28881	0,1927191	0	0,5950066	0,809842		1,260576
GSM2261723	0	0,822407	0,02376	0,396178	0	3,377912	0,10553007	0	0,6421687	0,767491		0,846167
GSM2261724	0	0,754453	0	0,375095	0	0,820176	0,16917731	0	0,5032948	0,865261		0,754453
GSM2261727	0	0,86947	0,000157	0,739297	0	0,813156	0,35210826	0	0,4267358	0,905036		0,869627
GSM2261728	0	0,886498	0	0,62018	0	1,973243	0,15670444	0	0,5378819	0,842901		0,886498
GSM2261730	0	0,975358	0	0,533829	0	1,143828	0,38238616	0	0,4678298	0,883597		0,975358
GSM2261731	0	0,710287	0	0,601762	0	1,350206	1,06196951	0	0,5493692	0,835455		0,710287
GSM2261733	0	0,539116	0,010832	0,561286	0	1,244183	0,53524498	0	0,5461101	0,837619		0,549947
GSM2261736	0	0,782602	0	0,619788	0	1,472651	0,15516873	0	0,4419507	0,897488		0,782602
GSM2261740	0	0,703655	0	0,576057	0,013777	1,374723	0,24994155	0	0,4830352	0,874637		0,703655
GSM2261744	0	0,823726	0,037222	0,82626	0	1,614581	0,43221829	0	0,4934046	0,868633		0,860947
GSM2261749	0	0,716183	0	1,008625	0	1,991718	0,2212506	0	0,5577714	0,829592		0,716183
GSM2261751	0	1,036691	0	0,766802	0	2,714651	0,3229368	0	0,5897116	0,809881		1,036691
GSM2261754	0	0,929729	0	0,606503	0	1,096054	0,42071517	0	0,5313333	0,849214		0,929729
GSM2261755	0	0,877843	0	0,541032	0	1,404835	0,24259664	0	0,4600442	0,887565		0,877843
GSM2261759	0	1,799296	0	0,51819	0	3,76654	0	0	0,5628665	0,827744		1,799296
GSM2261761	0	1,393163	0,031521	1,064075	0	1,59715	0,74502943	0	0,3553483	0,940864		1,424684
GSM2261764	0	1,209939	0	1,109323	0	1,572261	0,60269523	0	0,483694	0,874215		1,209939
GSM2261767	0	1,888293	0	1,325339	0	4,269636	0,73372971	0	0,6105078	0,798304		1,888293
GSM2261769	0	1,118174	0,011288	1,331906	0	1,93405	0,72861464	0	0,5389635	0,842323		1,129462
GSM2261770	0	1,087045	0	1,044993	0	1,648115	0,49285145	0	0,5190406	0,854416		1,087045
GSM2261772	0	0,870871	0	0,497607	0	1,093835	0,38025994	0	0,4421181	0,896892		0,870871
GSM2261773	0	1,067698	0	0,668575	0	1,978191	0,33530254	0	0,5601046	0,830906		1,067698
GSM2261774	0	1,061541	0	0,70286	0	1,486103	0,39710574	0	0,5008167	0,865473		1,061541
GSM2261777	0	1,360532	0,061408	0,680954	0,021447	4,095716	0,26850224	0	0,6777107	0,74644		1,42194
GSM2261778	0	1,428505	0	1,091514	0	1,553037	0,60705319	0	0,4872521	0,872683		1,428505
GSM2261779	0	1,022638	0	0,556212	0	1,710361	0,17206864	0	0,4701671	0,882343		1,022638
GSM2261781	0	1,446289	0,027112	0,876303	0	1,707906	0,31187169	0	0,396338	0,920384		1,473401
GSM2261783	0	1,232568	0	1,160756	0	5,496128	0	0	0,6726576	0,744748		1,232568
GSM2261784	0	1,23352	0	0,664052	0	0,871835	0,55690105	0	0,3690813	0,933556		1,23352
GSM2261785	0	1,585562	0,486096	0,841734	0	2,94737	0,13704368	0	0,5882136	0,815089		2,071658
GSM2261786	0	0,795009	0	0,707016	0	2,361391	0,83482962	0	0,591802	0,807488		0,795009
GSM2261787	0	0,731784	0,602036	0,646718	0	2,943485	0,12147076	0	0,6046154	0,799569		1,333821
GSM2261788	0	0,666208	0,262651	0,765017	0	1,85132	0,72841648	0	0,5807227	0,816847		0,928859
GSM2261789	0	0,980915	0,285488	0,853806	0	2,11322	0,64468081	0	0,4291186	0,903895		1,266403
GSM2261790	0	0,938383	0,178491	0,66425	0	1,698112	0,43030422	0	0,499551	0,865706		1,116874
GSM2261792	0	1,013187	0	0,450079	0,006935	1,493876	0,31189654	0	0,3692487	0,935104		1,013187
GSM2261795	0	0,624147	0	0,790281	0	1,874505	0,55482687	0	0,5952146	0,805929		0,624147
GSM2261796	0	1,159373	0	0,744452	0	1,64413	0,17513222	0	0,4505615	0,892581		1,159373
GSM2261799	0	0,802469	0	0,789158	0	1,719011	0,28525474	0	0,5656993	0,826299		0,802469
GSM2261803	0	1,084783	0	0,867092	0	2,674469	0,10349239	0	0,5282532	0,848289		1,084783
GSM2261804	0	0,68776	0,069572	1,28162	0	1,808665	0,58738012	0	0,6182628	0,791454		0,757333
GSM2261806	0	0,993857	0,00479	0,466515	0	2,13482	0,16302104	0	0,5520775	0,834553		0,998647
GSM2261808	0	0,885413	0	0,682802	0	0,842425	0,15213522	0	0,3963517	0,921545		0,885413
GSM2261809	0	0,901648	0	0,362207	0,01859	3,056572	0	0	0,6142862	0,789484		0,901648
GSM2261810	0	0,904454	0,166751	0,666392	0	1,364954	0,55556328	0	0,4766003	0,878267		1,071206
GSM2261811	0	0,592747	0,189355	0,502553	0	1,441858	0,28821123	0	0,5296509	0,847947		0,782102
GSM2261813	0	1,027599	0	0,512491	0	1,792435	0,7854468	0	0,3513063	0,946729		1,027599
GSM2261814	0	0,652696	0,045357	0,723302	0	1,488072	0,76521457	0	0,5663551	0,825365		0,698053
GSM2261819	0	1,021012	0	0,517241	0	1,228205	0,222234	0	0,3695659	0,933831		1,021012
GSM2261825	0	0,968673	0	0,579133	0,015652	1,589058	0,07631701	0	0,504717	0,863006		0,968673
GSM2261827	0	0,474065	0,258336	0,497641	0	1,949842	1,47507408	0	0,615351	0,78935		0,732401
GSM2261829	0	1,166357	0,001287	0,653208	0	2,853716	0,00659731	0	0,5873989	0,811199		1,167644
GSM2261831	0	1,348745	0	0,509348	0	2,609963	0,28339474	0	0,5001605	0,865971		1,348745
GSM2261833	0	0,740838	0	0,676343	0	2,263092	0,05704009	0	0,6095571	0,795336		0,740838
GSM2261835	0	0,907218	0	0,512595	0	0,763331	0,3273741	0	0,4044414	0,916024		0,907218
GSM2261836	0	1,040414	0,031708	0,427848	0	1,945086	0	0	0,5172621	0,855992		1,072121
GSM2261838	0	0,490442	0	0,705252	0	0,779716	0,28584523	0	0,4601186	0,887492		0,490442
GSM2261846	0	0,892727	0,044522	0,665395	0	1,156069	0,10766994	0	0,5405976	0,842796		0,937248
GSM2261847	0	0,982226	0,069725	0,936865	0	2,366067	0,00513153	0,02	0,2070456	1,023101		1,051951
GSM2261848	0	0,479711	0	0,42732	0	1,391436	0,08601664	0	0,5425085	0,839174		0,479711
GSM2261852	0	0,922141	0,090897	0,916282	0	2,554124	0,50673746	0	0,3172743	0,966598		1,013038
GSM2261854	0	1,425317	0	0,848398	0	2,849185	0	0	0,5745233	0,821133		1,425317
GSM2261857	0	1,622493	0	0,535723	0	2,046492	0,16935621	0	0,4356592	0,901352		1,622493
GSM2261864	0	0,756955	0	0,704938	0	1,520701	0,46097389	0	0,4304391	0,90366		0,756955
GSM2261865	0	0,819474	0,046235	0,474986	0	1,896463	0,2019316	0	0,5624251	0,828012		0,865708
GSM2261868	0	0,723974	0	0,451045	0	2,270527	0	0	0,6524915	0,763674		0,723974
GSM2261870	0	1,114556	0	0,537834	0	2,020518	0,38229382	0	0,4699791	0,882658		1,114556
GSM2261871	0	0,811533	0	0,878937	0	1,803111	0,25309158	0	0,4814587	0,875448		0,811533
GSM2261872	0	0,821395	0,078663	0,629448	0	1,63189	0,34076448	0	0,5136798	0,857482		0,900057
GSM2261873	0	0,799093	0	0,330526	0	1,736508	0,01856096	0	0,48286	0,87638		0,799093
GSM2261879	0	0,779783	0	0,289815	0	0,93112	0,32074511	0	0,4673105	0,884427		0,779783
GSM2261880	0,001842	0,775632	0	0,563289	0	1,095932	0,0749932	0	0,330597	0,954901		0,775632
GSM2261884	0	1,011861	0	0,311047	0	2,490664	0	0	0,530529	0,847949		1,011861
GSM2261886	0	1,419208	0,214501	1,015447	0	3,038787	0,48720368	0	0,5152799	0,856498		1,63371
GSM2261887	0	1,130602	0	0,555602	0	1,930872	0,23862873	0	0,4315346	0,903496		1,130602
GSM2261892	0	0,905053	0	1,047041	0	2,773298	0,11343137	0	0,5826326	0,813359		0,905053
GSM2261895	0	0,655694	0	0,811647	0	1,220552	0,30711261	0	0,5276288	0,848669		0,655694
GSM2261897	0	1,276972	0	0,560453	0	1,330096	0,19659745	0	0,4217677	0,907771		1,276972
GSM2261898	0	1,021611	0	0,664871	0	2,063666	0,28023076	0	0,5271453	0,849692		1,021611
GSM2261899	0,004456	1,247001	0	0,794507	0	1,138212	0,43270817	0	0,4587644	0,8882		1,247001
GSM2261901	0	2,316034	0,304823	0,308564	0	3,897522	0	0	0,7196436	0,728777		2,620857

GSM2261902	0	1,084188	0,346441	0,877449	0	2,936804	0	0	0,6840242	0,74898		1,430629
GSM2261903	0	1,544813	0,006758	1,798645	0	5,431828	0,09812484	0	0,5790735	0,814911		1,551571
GSM2261904	0	0,77029	0,138003	0,484468	0	1,566017	0,24246181	0	0,4815911	0,87569		0,908293
GSM2261906	0	1,258689	0	0,731254	0	1,699025	0,33071415	0	0,4696933	0,882469		1,258689
GSM2261908	0	0,83413	0,084315	0,605088	0	1,039055	0,45750117	0	0,441654	0,896779		0,918444
GSM2261909	0	0,989088	0	0,665509	0	1,326014	0,26402308	0	0,4270284	0,904727		0,989088
GSM2261910	0	0,477237	0,16731	0,621774	0	1,325781	0,54814678	0	0,5534735	0,833148		0,644547
GSM2261912	0	1,006144	0,027949	0,83319	0	1,857474	0,25986759	0	0,4619535	0,886329		1,034093
GSM2261913	0	1,196698	0	0,774374	0	1,534395	0,56775014	0	0,4884523	0,872292		1,196698
GSM2261916	0	0,679217	0,001404	0,759889	0	2,08819	0,21797778	0	0,5911319	0,808104		0,680621
GSM2261917	0	0,944026	0	0,681922	0	1,457985	0,26135023	0	0,4566155	0,889185		0,944026
GSM2261918	0	0,819083	0	0,86938	0	2,19127	0,16841883	0	0,625186	0,786979		0,819083
GSM2261920	0	1,181169	0	0,505217	0	2,3204	0,18838617	0	0,5645381	0,827573		1,181169
GSM2261921	0	0,642343	0	0,652193	0	1,330461	0,22352897	0	0,4701074	0,88181		0,642343
GSM2261922	0	0,888919	0	0,926142	0	1,563562	0,21566285	0,27	0,0982178	1,069497		0,888919
GSM2261923	0	0,727003	0	0,478588	0	1,402492	0,13395364	0	0,5005883	0,865071		0,727003
GSM2261924	0	0,846174	0,539701	0,501103	0	2,724716	0	0	0,5948551	0,806592		1,385875
GSM2261925	0	0,809248	0,01167	0,504473	0	1,413886	0,25226829	0	0,530595	0,848364		0,820918
GSM2261928	0	0,960463	0	1,487887	0	2,654151	1,66861423	0	0,6368631	0,778209		0,960463
GSM2261935	0	0,965051	0,140334	0,48699	0	2,385694	0,04483987	0	0,5993144	0,80421		1,105385
GSM2261939	0	0,822275	0	0,641335	0	2,45101	0,59685311	0	0,6501716	0,768159		0,822275
GSM2261945	0	1,132871	0,08989	0,697772	0	2,362711	0,23277428	0	0,5158019	0,856299		1,222761
GSM2261951	0	0,759835	0	0,425621	0	1,264041	0,30906824	0	0,4501185	0,89308		0,759835
GSM2261953	0	1,476843	0	0,686491	0	1,678512	0,2271987	0	0,4583452	0,888939		1,476843
GSM2261955	0	0,897569	0	0,61749	0	1,749637	0	0	0,5246564	0,851012		0,897569
GSM2261957	0	0,768126	0	0,309946	0	1,114386	0,23997832	0	0,4695108	0,883046		0,768126
GSM2261962	0	0,994896	0,146666	0,772693	0	3,151204	0,41848077	0	0,535473	0,843627		1,141562
GSM2261963	0	0,950046	0	0,770058	0	1,117025	0,31894724	0	0,4444857	0,895461		0,950046
GSM2261964	0	0,912079	0,242453	1,084314	0	2,796534	0,09255368	0	0,5097905	0,858975		1,154531
GSM2261966	0	1,113108	0	0,780292	0	1,134848	0,46436588	0	0,3826069	0,927091		1,113108
GSM2261967	0	0,725047	0	0,892022	0	1,784642	0,25931823	0	0,4510051	0,892944		0,725047
GSM2261976	0	1,06205	0	0,385143	0,020712	1,218181	0,23572282	0	0,4969954	0,868953		1,06205
GSM2261977	0	1,468317	0	1,08309	0	3,201934	0,27803523	0	0,5332475	0,845713		1,468317
GSM2261985	0	1,030853	0	0,720145	0	4,23273	0	0	0,725332	0,702014		1,030853
GSM2261988	0	1,122696	0	1,007256	0	1,356064	0,79437342	0	0,4155771	0,910635		1,122696
GSM2261989	0	0,8714	0	0,652034	0	1,195855	0,17599759	0	0,3942484	0,92196		0,8714
GSM2261991	0	0,732335	0	0,510898	0	1,024329	0,36265317	0	0,397588	0,919879		0,732335
GSM2261992	0	1,19863	0	0,637161	0	1,262903	0,31409884	0	0,3866439	0,924998		1,19863
GSM2261996	0	0,661072	0,409666	0,460017	0	1,56458	0,67336413	0	0,4878526	0,871913		1,070738
GSM2261998	0	1,19384	0	1,050792	0	1,200389	0,34492663	0,32	0,0869392	1,071926		1,19384
GSM2262001	0	0,988677	0	0,513125	0	1,167649	0,21339416	0	0,4645449	0,885459		0,988677
GSM2262003	0	1,154311	0,124608	0,687246	0	1,557786	1,26336332	0	0,5558548	0,833846		1,278919
GSM2262004	0	0,854783	0	0,556237	0	0,869571	0,44685177	0	0,421385	0,907345		0,854783
GSM2262007	0	0,874289	0,004594	0,648578	0	2,281474	0,09873271	0	0,5794066	0,816283		0,878884
GSM2262013	0	0,955026	0,096953	0,666804	0	1,788337	0,2049772	0	0,4700908	0,881977		1,051979
GSM2262017	0	0,75196	0,031199	0,503901	0	1,735243	0,25882795	0	0,4450849	0,896743		0,78316
GSM2262020	0	0,75915	0	0,626022	0	1,080384	0,37566128	0	0,4629899	0,885545		0,75915
GSM2262023	0	1,044303	0	0,71441	0	1,131131	0,36551717	0	0,3747954	0,931054		1,044303
GSM2262025	0	0,997389	0	0,688168	0	2,25841	0,20790942	0	0,6199034	0,792073		0,997389
GSM2262026	0	0,748002	0	0,70797	0	0,828847	0,45421099	0	0,4048642	0,916389		0,748002
GSM2262028	0	1,211892	0	0,79185	0	1,537028	0,41121897	0	0,451529	0,891864		1,211892
GSM2262030	0	0,697469	0	0,470002	0	0,899182	0,12749642	0	0,4604197	0,887231		0,697469
GSM2262031	0	1,404471	0	1,084138	0	1,506572	0,21109153	0	0,3336406	0,953516		1,404471
GSM2262033	0	0,668875	0	0,503218	0	1,251471	0,05155271	0	0,4415626	0,897947		0,668875
GSM2262034	0	0,992888	0,007238	0,577963	0	1,121575	0,16594617	0	0,3632424	0,937107		1,000126
GSM2262035	0	0,94901	0	0,844395	0	1,495759	0,33720228	0	0,47578	0,878573		0,94901
GSM2262041	0	0,733097	0	0,883498	0	1,079077	0,37248603	0	0,5056593	0,861458		0,733097
GSM2262042	0	1,115726	0,202221	0,577477	0	3,458421	0,40242242	0	0,5178231	0,855071		1,317946
GSM2262044	0	0,96761	0,051913	0,595162	0	1,159649	0,21838261	0	0,4761532	0,878985		1,019523
GSM2262046	0	0,78126	0	0,528244	0	0,810816	0,20255851	0	0,3509421	0,943222		0,78126
GSM2262049	0	1,997731	0,09569	0,637218	0	10,41359	0	0	0,6882746	0,726453		2,093421
GSM2262051	0	0,891794	0	0,366411	0,022916	0,992199	0,39197547	0	0,3803882	0,928085		0,891794
GSM2262053	0	0,918094	0	0,790105	0	0,755574	0,55599042	0	0,3749055	0,932024		0,918094
GSM2262059	0	1,279507	0,012219	1,03561	0	3,202523	0,07999983	0	0,5463267	0,837194		1,291727
GSM2262060	0	0,740619	0	0,579549	0	0,874912	0,1554756	0	0,3567773	0,941196		0,740619
GSM2262061	0	0,854742	0	0,95183	0	1,434858	0,30856822	0	0,4351719	0,900894		0,854742
GSM2262063	0	1,238529	0	0,568641	0	1,411401	0,08188231	0	0,3871657	0,925586		1,238529
GSM2262064	0	0,5772	0,021868	0,535378	0	1,367819	0,13885603	0	0,4257767	0,90712		0,599068
GSM2262067	0	0,638993	0	0,304798	0,00733	1,313501	0,13443456	0	0,5007263	0,865277		0,638993
GSM2262068	0	0,868868	0	0,429095	0	1,46519	0,25597316	0	0,4565132	0,889888		0,868868
GSM2262077	0	0,878206	0	0,413007	0,010621	0,884433	0,31025454	0	0,3212869	0,95636		0,878206
GSM2262078	0	0,915356	0	0,599699	0	0,943881	0,41025768	0	0,4866052	0,87344		0,915356
GSM2262080	0	1,082445	0,007507	0,706337	0	1,384505	0,31152702	0	0,3586326	0,939376		1,089952
GSM2262082	0	0,786648	0,034523	0,620722	0	1,330271	0,13141001	0	0,4364645	0,900131		0,821171
GSM2262083	0	0,5536	0	0,625849	0	1,341952	0,28198768	0	0,5079103	0,860149		0,5536
GSM2262086	0	1,251756	0	0,665837	0,013681	2,562245	0,26586461	0	0,5383725	0,843068		1,251756
GSM2262091	0	0,837325	0	0,454944	0	2,154776	0	0	0,4982298	0,867255		0,837325
GSM2262094	0	0,830983	0,236813	0,526107	0	1,92497	0,19863178	0	0,5159647	0,856076		1,067796
GSM2262098	0	0,917855	0	0,322578	0	1,347055	0,07497628	0	0,4216614	0,909028		0,917855
GSM2262102	0	1,892167	0,244372	1,195565	0	6,190982	0	0	0,5750324	0,817855		2,136539
GSM2262103	0	0,556983	0	0,463146	0	0,77447	0,39813433	0	0,4592268	0,887574		0,556983
GSM2262104	0	1,003895	0,074255	0,736957	0	2,899033	0,86376635	0	0,5562343	0,830695		1,07815
GSM2262105	0	1,1104	0	0,574988	0	1,98755	0,29232245	0	0,5068106	0,861925		1,1104

GSM2262107	0	0,768525	0,188627	0,567904	0	1,10875	0,33022187	0	0,5167811	0,856551		0,957153
GSM2262116	0,071006	0,503331	0,104396	0,508838	0,029971	3,229301	0,37066845	0	0,6550247	0,757527		0,607727
GSM2262117	0	1,347192	0	0,809178	0	1,866825	0,53564069	0	0,47924	0,877283		1,347192
GSM2262119	0	1,482336	0,070913	0,462039	0	3,628647	0	0	0,661068	0,760206		1,553249
GSM2262120	0	1,014328	0,002695	0,676499	0	1,113333	0,24111748	0	0,470707	0,881799		1,017022
GSM2262122	0	1,205933	0	0,436243	0	1,033776	0,39749843	0	0,4097048	0,91372		1,205933
GSM2262123	0	1,214183	0,013542	0,80705	0	2,152357	0,30007557	0	0,5400997	0,84275		1,227725
GSM2262124	0	1,49866	0	0,481628	0	2,407643	0	0	0,4419429	0,898983		1,49866
GSM2262126	0	0,666995	0,612787	0,684053	0	3,347268	0	0	0,6633705	0,756865		1,279782
GSM2262127	0	1,044737	0	0,655605	0	1,599047	0,06828787	0	0,4311099	0,903232		1,044737
GSM2262131	0	1,010467	0	0,760769	0	1,490848	0,33485372	0	0,4406624	0,897578		1,010467
GSM2262138	0	0,906831	0	0,467936	0	1,457231	0,07908132	0	0,3899282	0,925325		0,906831
GSM2262144	0	1,531685	0	0,723081	0	2,038137	0,17801687	0	0,4193203	0,909286		1,531685
GSM2262146	0	1,192792	0	0,471769	9,15E-05	2,469551	0,05780982	0	0,5212913	0,853405		1,192792
GSM2262148	0,0125	0,858128	0	0,539764	0,044621	1,584324	0,15694091	0	0,5410698	0,841946		0,858128
GSM2262149	0	0,632833	0,220026	0,477891	0	1,95762	0,31902512	0	0,5743293	0,819805		0,852858
GSM2262152	0	1,392357	0	0,97428	0	1,881563	0,24865476	0	0,4589056	0,888003		1,392357
GSM2262159	0	0,939856	0	0,776889	0	1,462169	0,1617382	0	0,3755406	0,932241		0,939856
GSM2262161	0	1,22301	0	1,030175	0,009888	2,709068	0,1213651	0	0,5607044	0,828933		1,22301
GSM2262162	0	0,733395	0,031396	0,745858	0	2,000286	0,18389606	0	0,5826182	0,814449		0,764791
GSM2262163	0	0,967567	0	0,794056	0	1,654689	0,06356263	0	0,6166922	0,796243		0,967567
GSM2262164	0	1,071235	0	0,731609	0	1,849707	0,01993916	0	0,4566785	0,889652		1,071235
GSM2262166	0	1,187301	0,10715	0,815592	0	1,569603	0,24793154	0	0,50214	0,864665		1,294451
GSM2262168	0	0,646912	0	0,541411	0	2,363261	0,05305185	0	0,5470404	0,836218		0,646912
GSM2262173	0	0,941787	0	0,519986	0,006524	1,046827	0,11989811	0	0,3596405	0,938995		0,941787
GSM2262175	0	1,60675	0,082762	0,566384	0	4,05011	0	0	0,5932091	0,806795		1,689511
GSM2262176	0	0,57794	0	0,281887	0	1,262975	0,30704015	0	0,4821169	0,876197		0,57794
GSM2262177	0	0,667487	0	0,389047	0	1,443823	0,15583942	0	0,5015412	0,864605		0,667487
GSM2262179	0	1,318548	0	1,314434	0	2,218726	0,3232047	0	0,5280591	0,848883		1,318548
GSM2262180	0	1,01288	0,000414	0,741382	0	1,737297	0,23638062	0	0,503316	0,863555		1,013294
GSM2262183	0	1,076304	0	0,421185	0	0,7052	0,20649657	0	0,3437489	0,946435		1,076304
GSM2262185	0	1,265058	0	0,635848	0	1,441003	0,11151311	0	0,384678	0,926699		1,265058
GSM2262186	0	1,134208	0	0,634909	0	2,054652	0,1954571	0	0,4440619	0,89675		1,134208
GSM2262187	0	1,668276	0,405238	1,12844	0	3,629716	0	0	0,5882715	0,812983		2,073513
GSM2262191	0	1,329915	0	0,791147	0	1,681889	0,24841034	0	0,3040853	0,966383		1,329915
GSM2262193	0	0,876506	0,051225	0,914807	0	2,090565	0,06071233	0	0,5098974	0,859023		0,927731
GSM2262194	0	0,840302	0	0,540308	0	1,357506	0,25992104	0	0,4476145	0,894178		0,840302
GSM2262196	0	1,336333	0,530078	0,419921	0	2,29299	0,11741735	0	0,5938764	0,812888		1,866411
GSM2262200	0	1,293634	0	1,316874	0	2,698003	0	0	0,4214932	0,909665		1,293634
GSM2262201	0	0,942295	0	0,729397	0	1,881473	0,07116336	0	0,4604516	0,887635		0,942295
GSM2262203	0	1,199868	0,111221	0,843393	0	2,35552	0,43464218	0	0,4199396	0,909243		1,311088
GSM2262211	0	1,111797	0	0,575002	0	2,136058	0	0	0,4619132	0,887522		1,111797
GSM2262213	0	1,164335	0	0,836071	0	0,884448	0,28234096	0	0,3136758	0,963696		1,164335
GSM2262215	0	0,992825	0	0,701233	0	1,615962	0,03709813	0	0,3859745	0,927401		0,992825
GSM2262216	0	0,696042	0	0,520955	0	1,360147	0,08864406	0	0,401476	0,919817		0,696042
GSM2262219	0	0,929801	0	0,947784	0	4,6358	0	0	0,5214516	0,854933		0,929801
GSM2262221	0	1,37351	0,076271	0,859874	0	4,970959	1,09744861	0	0,6937203	0,732027		1,449781
GSM2262224	0	0,717845	0,022138	0,420624	0	2,096364	0,19898349	0	0,4893891	0,872758		0,739983
GSM2262227	0	0,750154	0,032509	1,527802	0,148816	3,387942	2,46535159	0	0,6385374	0,77279		0,782663
GSM2262228	0	1,045973	0	0,505603	0	2,133714	0,22085948	0	0,5490932	0,836705		1,045973
GSM2262231	0	1,168408	0,109973	0,802239	0	6,55247	2,34352053	0	0,6988897	0,720178		1,278381
GSM2262235	0	1,212078	0,005082	0,66673	0	2,305428	0,25445623	0	0,4779209	0,878122		1,21716
GSM2262237	0	1,738872	0,145395	0,572887	0	6,78856	0	0	0,6451119	0,765157		1,884268
GSM2262238	0	0,821748	0	1,018054	0	1,417356	0,65649893	0	0,40348	0,918029		0,821748
GSM2262239	0	0,936137	0	0,725308	0	1,614085	0,17524089	0	0,4315011	0,90292		0,936137
GSM2262241	0	0,999033	0	1,02416	0	2,890568	0,84362642	0	0,4644185	0,886191		0,999033
GSM2262242	0	1,19374	0	0,684329	0	1,219231	0,18858824	0	0,3867926	0,925352		1,19374
GSM2262243	0	1,264235	0	0,951588	0	1,316518	0,69980172	0	0,4067348	0,914912		1,264235
GSM2262246	0	1,279523	0	0,351899	0,035073	2,877814	0	0	0,5904901	0,809188		1,279523
GSM2262251	0	0,9681	0	0,668438	0	1,127431	0,39926092	0	0,3741888	0,931335		0,9681
GSM2262253	0	0,576111	0	0,35261	0	0,424801	0,12834332	0	0,3323123	0,953218		0,576111
GSM2262256	0	1,445917	0	0,851706	0	1,416875	0,25477772	0	0,3754936	0,931012		1,445917
GSM2262258	0	0,915204	0	0,733283	0	1,460124	0,65723919	0	0,4600763	0,88724		0,915204
GSM2262264	0	1,105952	0	0,736074	0,027525	1,254984	0,04205874	0	0,4431298	0,896665		1,105952
GSM2262265	0	1,255857	0	0,668201	0	1,321122	0,4982664	0	0,4040753	0,916165		1,255857
GSM2262266	0	1,164688	0	1,03678	0	1,532612	0,46068021	0	0,4296568	0,903319		1,164688
GSM2262268	0	0,883083	0	0,627582	0	1,028859	0,38824034	0	0,4538456	0,890527		0,883083
GSM2262270	0	0,8513	0	0,480793	0	0,933116	0,16291981	0	0,3166383	0,959711		0,8513
GSM2262274	0	1,261371	0	0,668132	0	2,172941	0	0	0,4404346	0,898985		1,261371
GSM2262276	0	0,785829	0	0,516969	0	1,270146	0,25911483	0	0,4626012	0,88609		0,785829
GSM2262282	0	0,899365	0	0,672457	0	1,708943	0	0	0,4254132	0,907108		0,899365
GSM2262290	0	0,755577	0	0,600102	0	1,788019	0,28925185	0	0,5509595	0,834812		0,755577
GSM2262293	0	1,097832	0	1,698028	0	1,665833	0,42648811	0	0,4097772	0,917322		1,097832
GSM2262297	0	1,216532	0	0,748998	0	2,307905	0,0224005	0	0,4349881	0,902066		1,216532
GSM2262303	0	0,586272	0,067519	0,592911	0	1,591799	0,33674886	0	0,5506549	0,834665		0,65379
GSM2262305	0	1,24077	0	0,969494	0	2,130675	0	0	0,4668311	0,883927		1,24077
GSM2262307	0	1,30512	0	0,812009	0	1,83351	0,11917472	0	0,5112922	0,859671		1,30512
GSM2262311	0	0,990851	0	0,743717	0,043452	1,082928	0,1556595	0	0,3985158	0,919988		0,990851
GSM2262312	0	0,95132	0	0,576254	0	0,957285	0,16043575	0	0,4177022	0,909678		0,95132
GSM2262313	0	0,890586	0	0,6426	0	1,26193	0,26313395	0	0,4714277	0,8812		0,890586
average	0,000275	1,020955	0,041167	0,711592	0,001856	2,005664	0,28087843					
