# Supplementary material for: EphA2 and phosphoantigen-mediated selective killing of medulloblastoma by γδT cells preserves neuronal and stem cell integrity
Source: Oncoimmunology. 2025 Apr 7;14(1):2485535. doi: 10.1080/2162402X.2025.2485535 (PMC11980450; doi:10.1080/2162402X.2025.2485535)
Supplement: Boutin et al_figS2_v2.pdf [file KONI_A_2485535_SM7962.pdf]

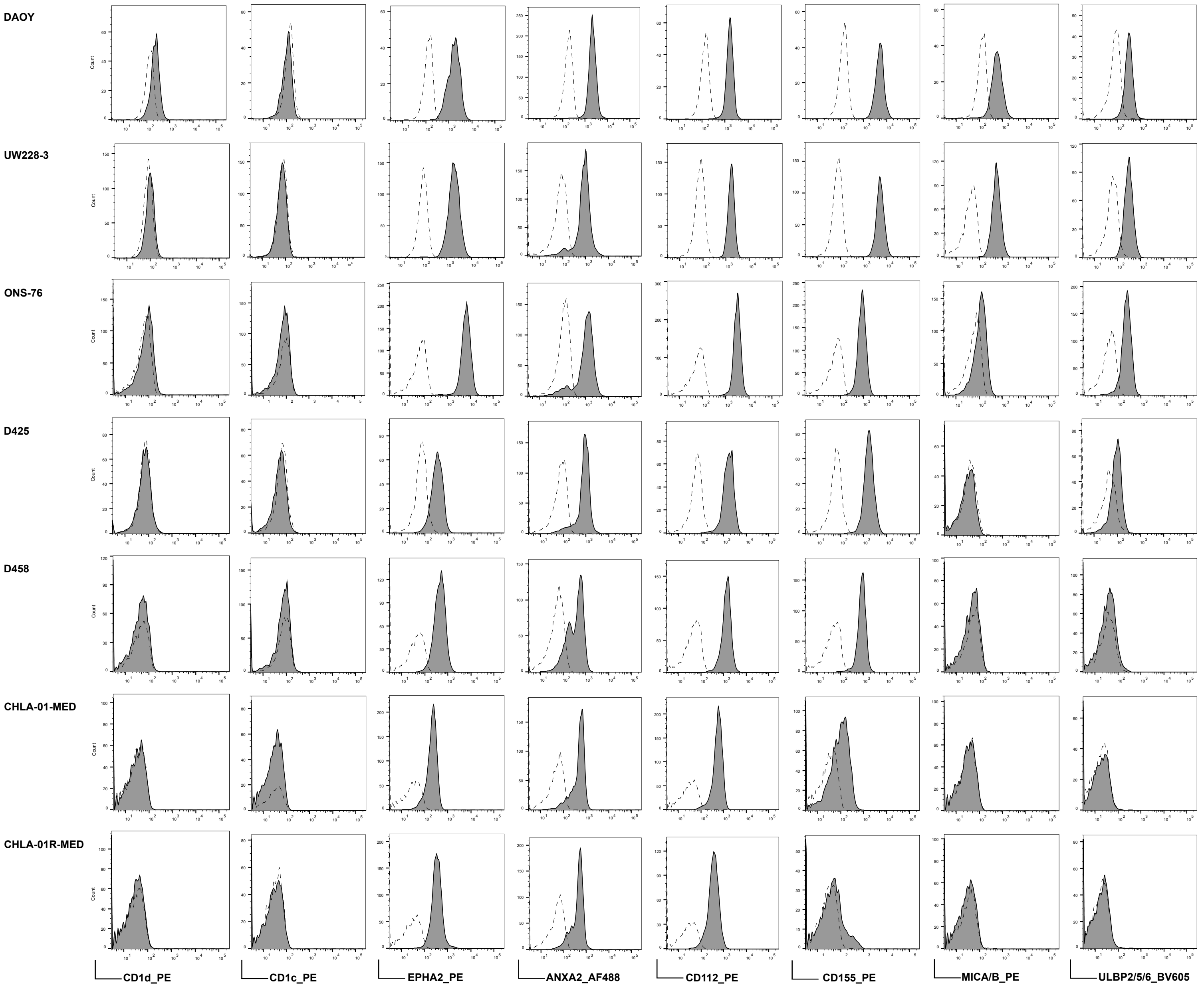

Figure S2: Representative histograms of the expression of CD1d, CD1c, Ephrin Type-A receptor 2 (EphA2), annexin A2 (ANXA2), CD112, CD155, MICA/B and ULBP2,5,6 on 7 MB cells lines. Isotype control (dashed line), antibody (full line).
