# Supplementary material for: EphA2 and phosphoantigen-mediated selective killing of medulloblastoma by γδT cells preserves neuronal and stem cell integrity
Source: Oncoimmunology. 2025 Apr 7;14(1):2485535. doi: 10.1080/2162402X.2025.2485535 (PMC11980450; doi:10.1080/2162402X.2025.2485535)
Supplement: Boutin et al_figS3.pdf [file KONI_A_2485535_SM7961.pdf]

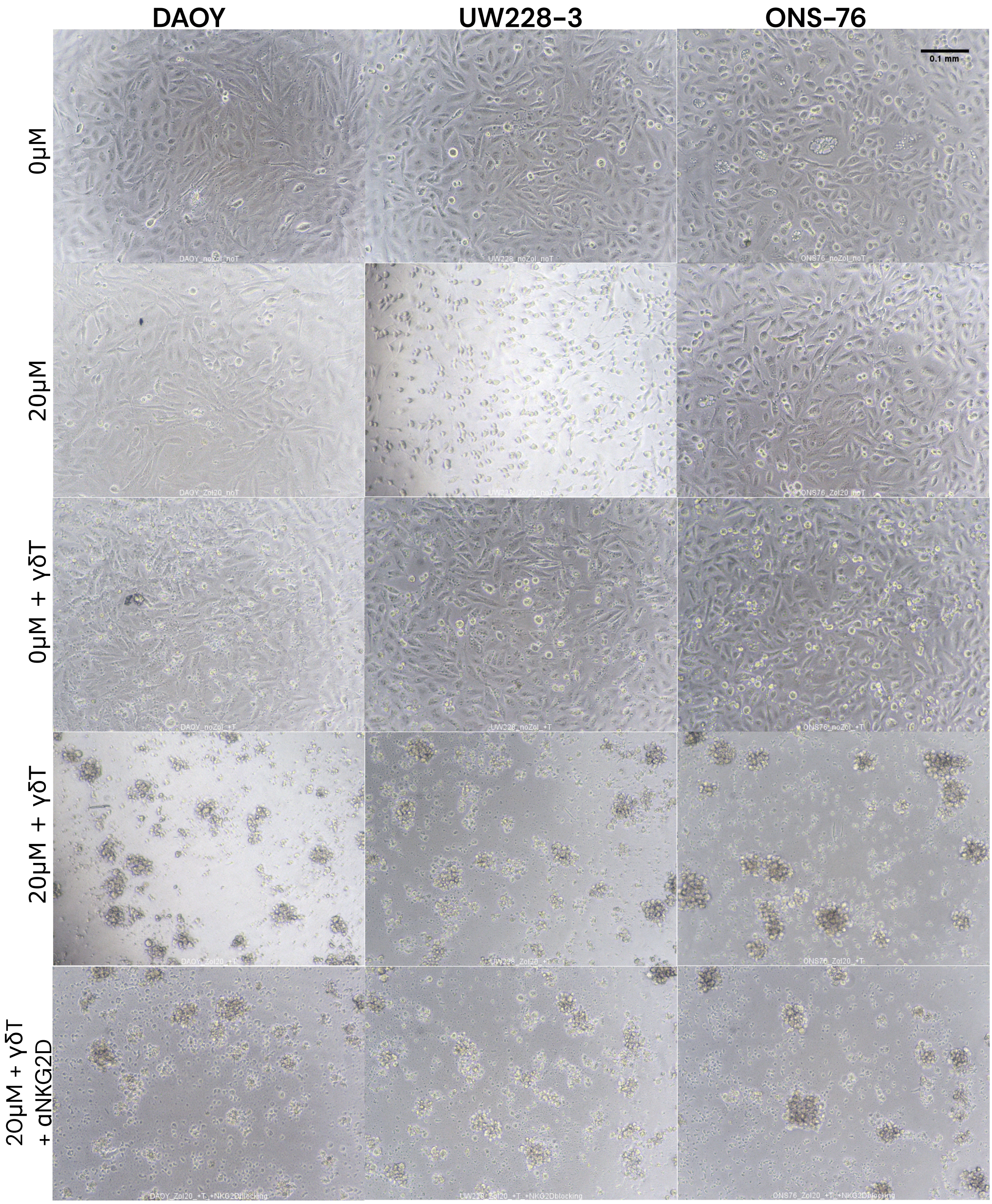

Figure S3: Brightfield images of DAOY, UW228 -3 and ONS -76 after 8 hours co-culture. MB treated with or without Zol treatment, anti-NKG2D antibody, and/or ex vivo expanded γδT cells. Scale bar = 0,1 mm [Nikon Eclipse Ts2, objective 10x, N.A 0.25].
