# Supplementary material for: EphA2 and phosphoantigen-mediated selective killing of medulloblastoma by γδT cells preserves neuronal and stem cell integrity
Source: Oncoimmunology. 2025 Apr 7;14(1):2485535. doi: 10.1080/2162402X.2025.2485535 (PMC11980450; doi:10.1080/2162402X.2025.2485535)
Supplement: Boutin et al_figS1_v2.pdf [file KONI_A_2485535_SM7960.pdf]

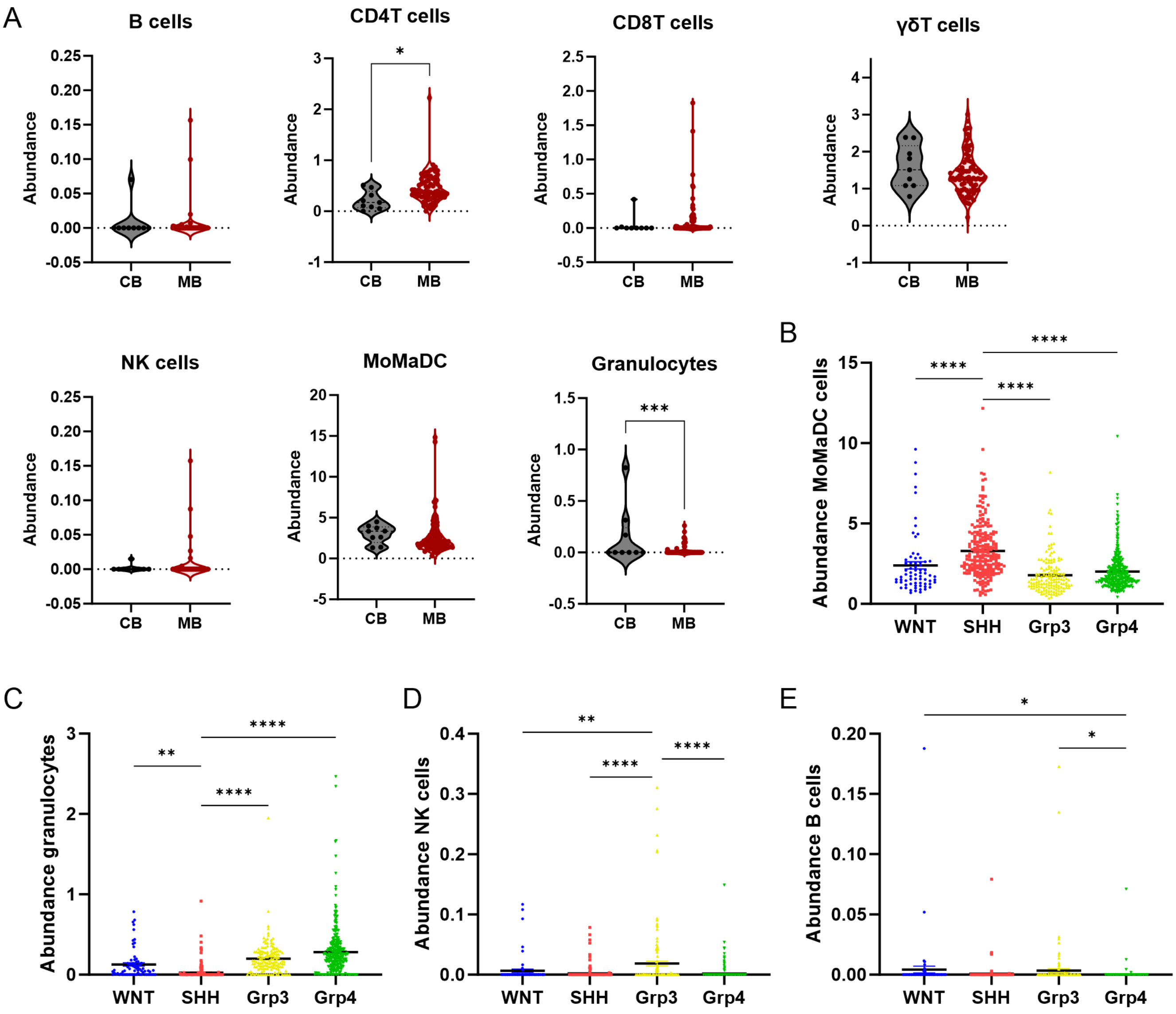

Figure S1: Immune cell abundance in healthy cerebellum and Medulloblastoma. **(A)** Abundance calculated after LM-7 CIBERSORT deconvolution of normal cerebellum (CB; n=9) versus MB patients (n=76). Statistical analysis was performed using Unpaired t-test (*$P < 0.05$) – nonsignificant differences are not displayed in the figure. **(B)** myeloid cell, **(C)** granulocyte, **(D)** NK cell and **(E)** B cell abundance across MB subgroups. **(B)**- **(E)** Statistical analysis was performed using two-way ANOVA followed by Tukey test to correct for multiplicity (*$P < 0.05$; **$P < 0.005$; ****$P < 0.0001$).
