# Supplementary material for: EphA2 and phosphoantigen-mediated selective killing of medulloblastoma by γδT cells preserves neuronal and stem cell integrity
Source: Oncoimmunology. 2025 Apr 7;14(1):2485535. doi: 10.1080/2162402X.2025.2485535 (PMC11980450; doi:10.1080/2162402X.2025.2485535)
Supplement: Boutin et al_Sup Mat_Met.pdf [file KONI_A_2485535_SM7959.pdf]

Cell culture

Neuroepithelial stem (NES) cells were obtained as previously described[20]. NES cells were cultured in flasks coated with 20 µg/ml poly-L-ornithine (Sigma, P3655) and 1 µg/ml laminin (Sigma, L2020) in complete neural stem cell medium (DMEM/F12+Glutamax (ThermoFisher, 31331093) supplemented with 1% N2 supplement (ThermoFisher, 17502001), 0.1% B27 supplement (ThermoFisher, 17504044), 10 ng/ml FGF2 (Qkine, Qk053), 10 ng/ml EGF (PeproTech, AF100-15) and 1% penicillin-streptomycin (Sigma, P4333)). For neuron differentiation, NES cells were seeded in flat bottom 96 well-plates coated with 20 µg/ml poly-L-ornithine and 2 µg/ml laminin at a concentration of $15x10^3$ cells/well in complete NES media. After 24 hours, NES media was removed and replaced with neuron differentiation media (DMEM/F12+Glutamax with 1% N2 supplement, 0,1% B27 supplement and 1 µg/ml laminin). NES cells were maintained in neuron differentiation media for a minimum of 3 weeks to ensure complete neuronal differentiation.

CHLA-01-MED and CHLA-01R-MED cells were cultured in DMEM/F12+Glutamax with 20 ng/ml, 20 ng/ml EGF, 2% B27. DAOY and ONS-76 cells were maintained in DMEM (ThermoFisher, 41966052) supplemented with 10% FBS (Hyclone, SV30160.03HI). Additionally, medium for DAOY cells contains 1% MEM non-essential amino acids, 1% HEPES, and 1% Glutamax (ThermoFisher, cat. 11140050, SH30237.01, and 35050061, respectively). UW228-3 cells were cultured in RPMI-1640+Glutamax (ThermoFisher, 61870044) supplemented with 10% FBS. D425 and D458 cells were cultured in DMEM/F12+Glutamax supplemented with 10% FBS. All cell lines tested negative for mycoplasma and were authenticated by STR analysis at Eurofins (DAOY, D425, D458 CHLA-01-MED, CHLA-01R-MED), or Multiplexion (ONS-76, UW228-3). Grp3-MB Patient-Derived Xenograft (PDX) MB-LU-181 was established and cultured as described[21].

Jurkat JRT3 expressing Vγ9Vδ1-MAU TCR were obtained as described and cultured in RPMI-1640+Glutamax supplemented with 10% FBS[22].

Sorting and *ex-vivo* expansion of γδT cells

Human peripheral blood mononuclear cells (PBMCs) were isolated from anonymized buffy coat from healthy doners obtained from the department of Klinisk Immunologi och transfusionsmedicin at Karolinska University Hospital (Stockholm, Sweden). Local regulations states that working with blood from anonymous healthy human doners requires no ethical permit. PBMCs were collected by Ficoll gradient centrifugation (Ficoll-Paque® PREMIUM, Cytiva, 17-5442-02) and resuspended in RPMI-1640 medium supplemented with 10% heat-inactivated FBS. γδT cells were magnetically sorted using EasySep™ Human Gamma/Delta T Cell Isolation Kit (Stemcell Technologies, 19255) according to the manufacturer's protocol. Sorted fraction was expanded by activation with tetramer CD2/CD3/CD28 (Immunocult®, Stemcell Technologies, 10970) in ImmunoCult-XF T Cell Exp Medium (Stemcell Technologies, 10981) supplemented with 300IU/ml recombinant human IL-2 (Stemcell Technologies, 78036) for 14 days. Expanded γδT cell purity was assessed by flow cytometry by staining with anti-

pan γδ TCR mAb and the expanded populations were kept for further experiments if γδT > 85%. After expansion, γδT cells were maintained in ImmunoCult-XF T Cell Exp Medium supplemented with 300IU/ml recombinant human IL-2 for up to three weeks.

Antibodies

TARGET	CONJUGATED	CLONE	SUPPLIER
CD107a	PerCP-Cy5.5	#H4A3	Biolegend
pan γδ TCR	FITC	#IMM510	Beckman Coulter
Vδ2	BV421	#B6	Biolegend
Vδ1	APC	#REA173	Miltenyi Biotec
CD69	PE	#FN50	Biolegend
CD1d	PE	#51.1	Biolegend
CD1c	PE	#L161	Biolegend
EphA2	PE	#SHM16	Biolegend
CD112	PE	#TX31	Biolegend
CD155	PE	#SKII.4	Biolegend
MICA-B	PE	#6D4	BD Biosciences
ULBP2,5,6	BV605	#165903	BD Biosciences
Mouse IgG2b isotype	PE	#MPC-11	Biolegend
Mouse IgG1 isotype	PE	#MOPC-21	Biolegend
Mouse IgG2a isotype	BV605	#G155-178	BD Biosciences
Mouse IgG2a isotype	PE	#G155-178	BD Biosciences
NKG2D	Purified	#1D11	Nordic BioSite
Annexin A2	Purified	# 1C1E12	Proteintech
Mouse IgG2a isotype	Purified	# 11A1B2	Proteintech
Donkey anti-mouse	AF488	polyclonal	Life Technologies

Statistical analysis

Samples collected from anonymized healthy human donors have been used in this study. Data were analyzed with GraphPad Prism software v.10.1.2 and are presented as mean ± SD; or survival curve. Statistical analysis data was performed by unpaired nonparametric t-student test; log-rank test; or by two- and one-way ANOVA followed by Tukey, Sidák, or two-tailed Dunnett's tests to correct for multiplicity. *P*-values below 0.05 were considered statistically significant.
